# Mini-dCas13X–mediated RNA editing restores dystrophin expression in a humanized mouse model of Duchenne muscular dystrophy

**DOI:** 10.1172/JCI162809

**Published:** 2023-02-01

**Authors:** Guoling Li, Ming Jin, Zhifang Li, Qingquan Xiao, Jiajia Lin, Dong Yang, Yuanhua Liu, Xing Wang, Long Xie, Wenqin Ying, Haoqiang Wang, Erwei Zuo, Linyu Shi, Ning Wang, Wanjin Chen, Chunlong Xu, Hui Yang

**Affiliations:** 1 HuiGene Therapeutics, Shanghai, China.; 2 Institute of Neuroscience, State Key Laboratory of Neuroscience, Key Laboratory of Primate Neurobiology, CAS Center for Excellence in Brain Science and Intelligence Technology, Chinese Academy of Sciences, Shanghai, China.; 3 Department of Neurology, First Affiliated Hospital, Fujian Medical University, Fuzhou, China.; 4 Lingang Laboratory, Shanghai, China.; 5 Shenzhen Branch, Guangdong Laboratory for Lingnan Modern Agriculture, Genome Analysis Laboratory of the Ministry of Agriculture, Agricultural Genomics Institute at Shenzhen, Chinese Academy of Agricultural Sciences, Shenzhen, China.; 6 Shanghai Research Center for Brain Science and Brain-Inspired Intelligence, Shanghai, China.

**Keywords:** Therapeutics, Gene therapy, Monogenic diseases, Muscle

## Abstract

Approximately 10% of monogenic diseases are caused by nonsense point mutations that generate premature termination codons (PTCs), resulting in a truncated protein and nonsense-mediated decay of the mutant mRNAs. Here, we demonstrate a mini-dCas13X–mediated RNA adenine base editing (mxABE) strategy to treat nonsense mutation–related monogenic diseases via A-to-G editing in a genetically humanized mouse model of Duchenne muscular dystrophy (DMD). Initially, we identified a nonsense point mutation (c.4174C>T, p.Gln1392*) in the *DMD* gene of a patient and validated its pathogenicity in humanized mice. In this model, mxABE packaged in a single adeno-associated virus (AAV) reached A-to-G editing rates up to 84% in vivo, at least 20-fold greater than rates reported in previous studies using other RNA editing modalities. Furthermore, mxABE restored robust expression of dystrophin protein to over 50% of WT levels by enabling PTC read-through in multiple muscle tissues. Importantly, systemic delivery of mxABE by AAV also rescued dystrophin expression to averages of 37%, 6%, and 54% of WT levels in the diaphragm, tibialis anterior, and heart muscle, respectively, as well as rescued muscle function. Our data strongly suggest that mxABE-based strategies may be a viable new treatment modality for DMD and other monogenic diseases.

## Introduction

The majority of monogenic diseases are caused by point mutations (1), among which approximately 50% can be reversed by A-to-G conversion with adenine base editors (ABEs). Specifically, A-to-G conversion can restore protein expression by converting premature termination codons (PTCs) to non-termination codons, thus enabling PTC read-through. Nonsense mutations that introduce PTCs, UAG, UGA, and UAA, account for approximately 10% of monogenic diseases (2). Although base editors derived from the nickase Cas9 have been demonstrated to correct some monogenic mutations, these proteins are generally too large for efficient in vivo delivery by single adeno-associated virus (AAV) vectors (3, 4), which have a maximum cargo size of only 4.7 kb. Given the relatively small size and reversible nature of RNA editing activity, flexible RNA base editing tools have recently been the focus of intensive development efforts, resulting in REPAIR (5), RESTORE (6), LEAPER (7), mxABE (8), and other systems. While these tools are currently used exclusively as effective research tools, potential clinical applications of RNA base editing have been proposed as personalized gene therapies for monogenic diseases.

Among monogenic degenerative muscular diseases, Duchenne muscular dystrophy (DMD) represents the second most common hereditary muscular disease, affecting an estimated 1 in 3,500–5,000 male newborns (9). DMD still lacks any effective treatment and is often fatal. Statistically, 60%–70% of human DMD diseases are caused by exon deletions in the *DMD* gene (10), whereas approximately 10% of cases result from nonsense point mutations that introduce PTCs in *DMD*, of which 40% are UAG, 39% are UGA, and 21% are UAA PTCs (11). A recent study showed that RNA base editors can indeed restore dystrophin expression in mice with nonsense mutation–induced X-linked muscular dystrophy (mdx) (12). However, in that study, they achieved only limited dystrophin expression, and moreover, no alleviation of damaged muscle function in the treated mice was observed. Nonetheless, these results validated RNA editing modalities for targeting nonsense mutation–induced PTCs and encourage the development of more effective approaches to potentially treat monogenic diseases such as DMD.

In this study, we identified an uncharacterized c.4174C>T mutation that introduced a PTC in the *DMD* gene of a patient with progressive muscle weakness. We validated that this nonsense mutation caused loss of dystrophin in multiple muscle tissues in a genetically humanized mouse model of DMD. We then applied the mini-dCas13X–based RNA base editor, mxABE, to convert the TAG codon to a TGG codon in *DMD* mRNA, which efficiently restored dystrophin expression in the skeletal, heart, and diaphragm muscular tissue of humanized DMD mice. In addition, systemic or local administration of mxABE in an AAV vector significantly improved muscular growth and function in humanized DMD mice. Notably, in cultured cells, mxABE also exhibited high editing activity of other pathogenic nonsense mutations that have been documented in patients with DMD, suggesting its versatility for treating different DMD subtypes. Cumulatively, these findings demonstrate that mxABE is an efficient RNA base editor of known PTCs in DMD in vitro and in vivo and prompt further studies toward developing mxABE technology for personalized gene therapy to treat monogenic diseases.

## Results

### Validation of c.4174C>T as the causative nonsense DMD mutation in a humanized DMD mouse model.

An 8-year-old male patient with progressive limb weakness came to our clinic for the first diagnosis. His medical history indicated onset of abnormal walking posture at age 4. Other symptoms included tiptoe walking, inability to climb stairs, and difficulty standing up after squatting, all of which presented with progressive exacerbation. To exclude neurological causes, we performed physical and psychiatric examinations. The patient was sober without obvious mental illness. His proximal and distal muscle strength levels were IV and V, respectively. No abnormalities were found upon cranial nerve examination, and tendon reflexes were normal. No abnormalities that suggested sensory ataxia were observed. Overall, no evident pathological signs were elicited during these examinations; however, the patient was positive for Gowers’ sign. Bilateral calf muscles displayed hypertrophy and stiffness, and mild contractures in both Achilles tendons were also observed. His walking speed was 45.5 m/min, and his North Star Ambulatory Assessment score was 18 points. Electromyography examination revealed muscle-induced damage and low compound muscle action potential volatility of upper- and lower-limb movements.

**Figure 1:**
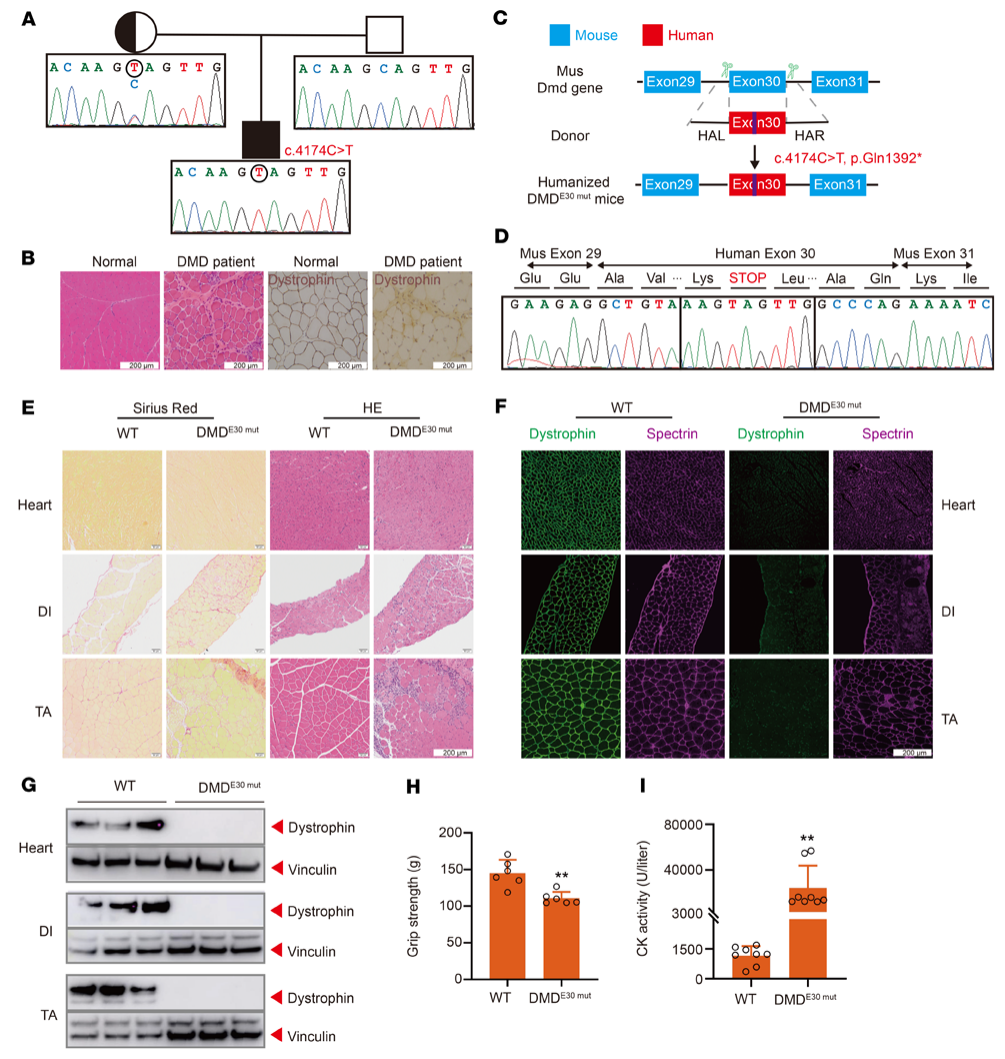
Establishment and characterization of a humanized DMD mouse model. (**A**) Pedigree of patient with DMD (proband) with a nonsense mutation p.Gln1392*. Squares represent males; circles represent females. (**B**) Histological analysis of left biceps muscle from normal and proband. Dystrophin (MilliporeSigma, D8168) is shown in brown. (**C**) Strategy for generating humanized DMD mouse model. CRISPR/Cas9 editing using 2 sgRNAs flanking an exon was used to delete mouse *Dmd* exon 30 and replace it with human *DMD* exon 30 carrying the nonsense mutation. (**D**) RT-PCR products from muscle of DMD^E30mut^ mice were sequenced to validate the exon 30 mutation. (**E**) Sirius red staining and H&E staining of TA, DI, and heart muscle of WT and DMD^E30mut^ mice. (**F**) Dystrophin immunohistochemistry from indicated muscles of WT and DMD^E30mut^ mice. Dystrophin (Abcam, ab15277) and spectrin (Millipore, MAB1622) are shown in green and magenta, respectively. (**G**) Western blot confirming the absence of dystrophin in indicated muscle tissues. (**H**) WT and DMD^E30mut^ mice were subjected to forelimb grip strength testing to measure muscle performance (*n* = 6). (**I**) Serum CK, a marker of muscle damage and membrane leakage, was measured in WT and DMD^E30mut^ mice (*n* = 8). All mice were 8 weeks old at the time of the experiment. Data are represented as mean ± SEM. Each dot represents an individual mouse. **P < 0.01 using unpaired 2-tailed Student’s *t* test. Scale bars: 200 μm.

To investigate the etiology of his muscle-related dysfunction, we measured blood lactate dehydrogenase and creatinine kinase (CK) levels and found that both were extremely elevated (851 U/L and 13,342 U/L, respectively), consistent with DMD-like symptoms. We next performed multiplex ligation-dependent probe amplification, which detected no copy number variation in the *DMD* gene. Therefore, whole-exome deep sequencing was used to screen for mutations in the protein-coding sequence that could be potentially responsible for muscle weakness in the patient. This analysis identified a nonsense mutation, c.4174C>T, p.Gln1392*, in exon 30 of the *DMD* gene (Figure 1A). DMD follows a Mendelian hereditary pattern, and the *DMD* gene is located on the X chromosome. The patient’s mother was heterozygous for the null allele resulting from this nonsense mutation and also carried a WT allele, whereas only the WT allele was present in the paternal genome (Figure 1A). We examined dystrophin expression by histological immunostaining of tissue from a biopsy of the left biceps muscle of the patient. This showed complete loss of dystrophin expression (Figure 1B), supporting a causative role of c.4174C>T in the pathological symptoms observed in the patient.

To further confirm the causal relationship between c.4174C>T and DMD pathology, we designed a genetic humanization strategy to generate a personalized mouse model of the effects of DMD^E30mut^ by replacing mouse *Dmd* exon 30 with the corresponding human exon from the patient with the c.4174C>T mutation (Figure 1C). A CRISPR-assisted knockin method was used to generate several founder mice with the correctly humanized genotype. To ensure that the human exon was correctly spliced with the flanking mouse exons, we performed reverse transcription PCR (RT-PCR) to check the junction sequence around exon 30, which confirmed that the expected splicing was seamless in the humanized DMD^E30mut^ mice (Figure 1D).

Because DMD-related mutations affect males more often than females, histological analysis with Sirius red and H&E staining was conducted in male DMD^E30mut^ mice. This showed that humanized DMD^E30mut^ mice had severe muscle wasting in the heart, diaphragm (DI), and tibialis anterior (TA) (Figure 1E). Also, the muscle fibers of DMD^E30mut^ mice exhibited significant variation in size with widened inter-fiber space, inward movement of cell nuclei, and abnormal infiltration of inflammatory cells compared with normal muscle, which are all characteristics of DMD. Immunostaining for dystrophin and spectrin expression in the heart, DI, and TA muscle of WT and DMD^E30mut^ mice showed that DMD^E30mut^ mice exhibited complete loss of dystrophin expression but maintained normal spectrin expression compared with WT mice (Figure 1F). Western analysis confirmed the absence of dystrophin in DMD^E30mut^ mice (Figure 1G). To examine muscle function in DMD^E30mut^ mice, we measured the grip strength of 8-week-old mice. These tests indicated that grip strength was significantly reduced in DMD^E30mut^ mice compared with WT mice (Figure 1H), consistent with the progressive limb weakness that is characteristic of human patients with DMD. Moreover, CK levels were strikingly higher in DMD^E30mut^ mice compared with WT mice (Figure 1I), implying severe muscle damage.

Overall, the humanized DMD^E30mut^ mice exhibited molecular and functional phenotypes that were highly similar to the phenotypes observed in patients with DMD, strongly supporting a causative role for the c.4174C>T mutation in the DMD symptoms of the patient. These findings indicate that DMD^E30mut^ mice accurately recapitulate the symptoms of DMD observed in human patients and validate them as a suitable model for subsequent evaluation of potential intervention strategies.

### mxABE can mediate RNA-level correction of c.4174C>T and other common DMD mutations.

**Figure 2:**
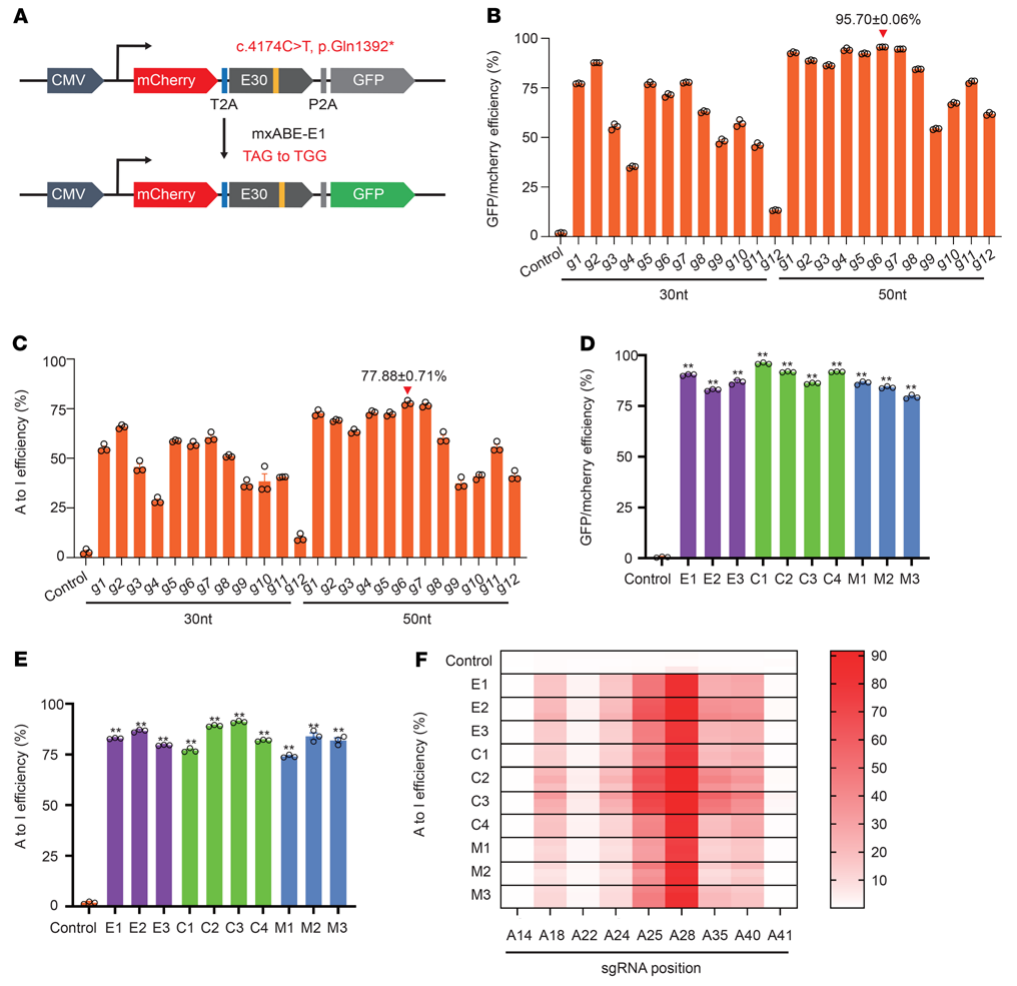
mxABE-mediated correction of mutant *DMD* RNA. (**A**) The reporter construct containing the mCherry cassette fused with a 2A peptide, mutant human exon 30 (c.4174C>T), and ATG-removed GFP. Correction of the stop codon within the target sequence allows GFP expression. (**B**) Flow cytometry analysis of GFP expression in HEK293T cells transfected with 24 gRNAs. (**C**) Deep sequencing of the reporter RNA transcribed from the reporter vector after GFP rescue experiment. (**D** and **E**) Comparison of the editing efficiencies of different mxABE vectors by flow cytometry (**D**) and deep sequencing (**E**). (**F**) Measurement of bystander A-to-I editing rate for multiple adenosines within a 50 nt region of the DMD^E30mut^ target sequence. gRNA g6 was used in the analysis. Adenosines (A) with position number are indicated from the 5′ to the 3′ end in the 50 nt target sequence. Data are represented as mean ± SEM (*n* = 3). **P < 0.01 using unpaired 2-tailed Student’s *t* test.

In our previous work, we developed the hypercompact RNA base editor mxABE, which can be packaged in an AAV vector for in vivo delivery of genetic therapies (8). Importantly, the c.4174C>T mutation fit the criteria for potential correction by mxABE-mediated A-to-G conversion. To identify the most effective guide RNA (gRNA) for targeting the c.4174C>T site, we engineered a dual fluorescence reporter for gRNA screening. Specifically, we integrated the genomic sequence from the patient containing the c.4174C>T mutation between *mCherry* and *GFP* reporters and expressed the dual reporter with mxABE in HEK293T cells (Figure 2A). The stop codon resulting from c.4174C>T induced premature translational termination, thus blocking GFP expression, which could be rescued by mxABE editing of the c.4174C>T mutation (Figure 2A). A total of 24 gRNAs, all either 30 or 50 nt long, were tested in our GFP rescue screen, and all of them resulted in detectable GFP fluorescence compared with an untargeted control gRNA. Among these, the 50 nt g6 gRNA exhibited the highest GFP rescue efficiency, restoring 95.70% of signal intensity on average (Figure 2B and Supplemental Figure 1, A and B; supplemental material available online with this article; https://doi.org/10.1172/JCI162809DS1). Deep sequencing of the reporter mRNAs after GFP rescue confirmed that g6 provided the most efficient editing, with a 77.88% A-to-G editing rate (Figure 2C and Supplemental Figure 1C).

To identify the optimal AAV-compatible expression construct for efficient RNA base editing, we assembled 10 mxABE expression cassettes (Supplemental Figure 2) with different promoters (i.e., EFS, CBh, MHCK7), single or dual direct repeats, different nuclear localization signals, and with or without a translational regulatory element (WPRE), and we tested them in GFP rescue assays in HEK293T cells. All 10 mxABE expression cassettes provided at least 79.5% GFP rescue and 79.08% average A-to-G conversion efficiency (Figure 2, D and E). Targeted sequencing of mutant *DMD* transcripts edited by the different mxABEs showed low, variable efficiency for A-to-G editing of adjacent A sites (Figure 2F and Supplemental Figure 3), which was consistent with previous studies of bystander editing activity by a truncated RNA base deaminase, ADAR2dd (5–7). Analysis of the bystander A-to-G editing events indicated that no premature stop codons were introduced at other sites in the *DMD* transcript by mxABE, suggesting that this approach should enable PTC read-through. To evaluate the transcriptome-wide off-target effects of mxABE targeting c.4174C>T, we conducted RNA sequencing (RNA-Seq) analysis of HEK29T cells expressing different mxABE cassettes driven by an EFS (E1), CBh (C3, C4), or MHCK7 (M2) promoter, and we evaluated a nontargeting mxABE as a control. Our results showed no significant differences in A-to-G/C-to-U single-nucleotide variant (SNV) counts generated by any of the different reporter constructs (851.67 ± 36.62) compared with the control (829.17 ± 28.09) (Supplemental Figures 4 and 5), suggesting high editing specificity for mxABE constructs to target c.4174C>T at the transcriptome scale.

To investigate the general efficacy of mxABE to rescue other types of DMD mutations, we designed mxABE gRNAs that targeted c.2977C>T (p.Gln993*, exon 23) and c.8009C>T (p.Trp2670*, exon 54), which are among the most common causative DMD mutations, and we tested these gRNAs using our RNA editing GFP rescue assay workflow. Among 62 different gRNAs targeting either c.2977C>T or c.8009C>T, two potent gRNAs were identified that provided 77.98% and 78.90% editing rates, respectively (Supplemental Figure 6). These results suggest that mxABE-based RNA editing could be employed for correction of different mutations that introduce PTCs in DMD.

### AAV delivery of mxABEs rescues dystrophin expression in mice.

**Figure 3:**
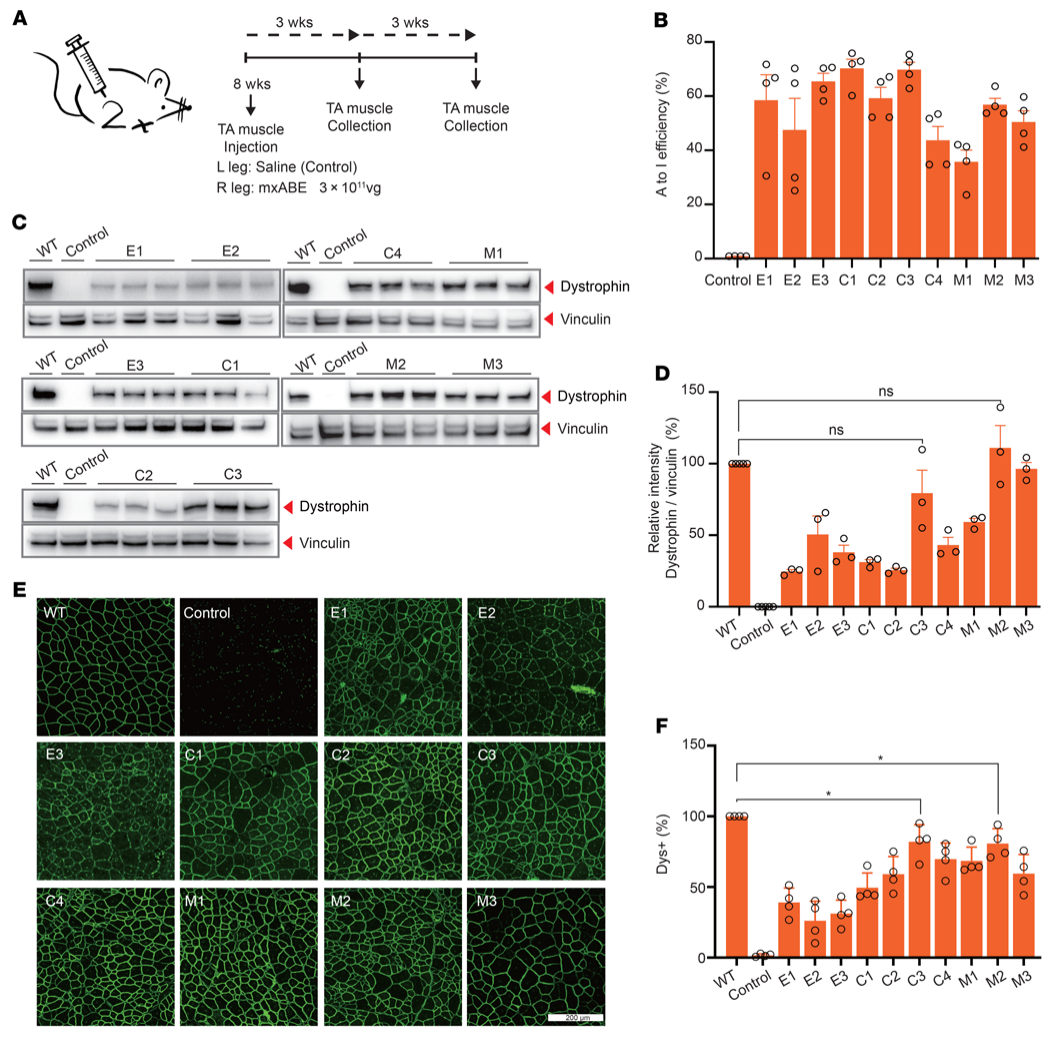
mxABE robustly rescues dystrophin expression in TA 3 weeks after AAV injection. (**A**) Overview of in vivo intramuscular (i.m.) injection of the AAV9-mxABE construct into the TA muscle of the right leg of 8-week-old DMD^E30mut^ mice. Left leg was injected with saline as a control. Arrows indicate time points for tissue collection after injection. (**B**) The A-to-I efficiency of different AAV9 vectors was measured (*n* = 4). A cDNA amplicon spanning exon 30 was generated from the TA muscle and analyzed by deep sequencing. (**C**) Western blot analysis of dystrophin (MilliporeSigma, D8168) and vinculin (Cell Signaling Technology, 13901S) expression in TA muscles 3 weeks after injection with AAV9-mxABEs or saline. (**D**) Quantification of dystrophin expression from Western blots after normalization to vinculin expression (*n* = 3). Age-matched WT and saline-treated DMD^E30mut^ mice were included as control. (**E**) Comparison of dystrophin expression among the 10 AAV9-mxABE systems by immunofluorescence. Dystrophin (Abcam, ab15277) is shown in green. Scale bar: 200 μm. (**F**) Quantification of Dys^+^ fibers in cross sections of TA muscles (*n* = 4). Data are represented as mean ± SEM. Each dot represents an individual mouse. Significance is indicated by an asterisk (*P* < 0.05). NS, not statistically significant using unpaired 2-tailed Student’s *t* test.

Given the high efficiency of c.4174C>T correction by mxABEs in vitro, we next tested the effectiveness of mxABEs in vivo in our humanized DMD^E30mut^ model. We generated 10 vector constructs (AAV9-mxABE-T) with their respective mxABEs driven by different expression cassettes, designated as E1-, E2-, E3-, C1-, C2-, C3-, C4-, M1-, M2-, or M3-mxABE (Supplemental Figure 2), and packaged each of them into AAV9 for in vivo transfection into muscle tissue. Specifically, the right TA of 8-week-old male DMD^E30mut^ mice was injected with 3 × 10^11^ vg of AAV9-mxABE viral particles targeting c.4174C>T, while the left TA of the same mouse was injected with saline as a control (Figure 3A). All mice were kept under identical conditions, and the cohort was divided in half and euthanized after 3 weeks or 6 weeks for molecular and histological analyses of muscle tissue (Figure 3A), including editing efficiency, Western analysis, and histological staining for dystrophin expression.

We first evaluated the short-term effects of mxABE treatment after 3 weeks. Deep sequencing of DNA extracted from mxABE-treated TA tissue from DMD^E30mut^ mice revealed an average editing rate of about 35% for all 10 constructs, and editing efficiency reached 70% in TA of mice treated with C1-mxABE or C3-mxABE (Figure 3B and Supplemental Figure 7, A and B). In general, EFS promoter–driven mxABEs (E1-, E2-, E3-mxABE) and CBh promoter–driven mxABEs (C1-, C2-, C3-, C4-mxABE) exhibited slightly higher rates of RNA correction compared with those driven by a muscle tissue–specific MHCK7 promoter (M1-, M2-, M3-mxABE) (Figure 3B and Supplemental Figure 7, A and B). Also, Western analysis of TA tissue indicated that dystrophin protein levels in DMD^E30mut^ mice treated with CBh- or MHCK7-mxABEs were similar to levels in WT mice; however, dystrophin protein expression was lower in EFS-mxABE–treated DMD^E30mut^ mice (Figure 3, C and D). We also found that base editor expression was higher in CBh- and MHCK7-mxABE–treated compared with EFS-mxABE–treated DMD^E30mut^ mice (Supplemental Figure 7C). Histological examination of TA tissue showed that all mxABE constructs potently rescued dystrophin expression (43.57% ± 21.31% of WT level) (Figure 3, E and F, and Supplemental Figure 8). In agreement with our Western analysis, among the mxABE constructs, the muscle texture of dystrophin-positive (Dys^+^) TA tissue treated with C3-mxABE (74.57% ± 16.04%) or M2-mxABE (68.35% ± 20.01%) was much more similar to that of WT TA tissue compared with the other mxABE constructs (Figure 3, E and F, and Supplemental Figure 8).

**Figure 4:**
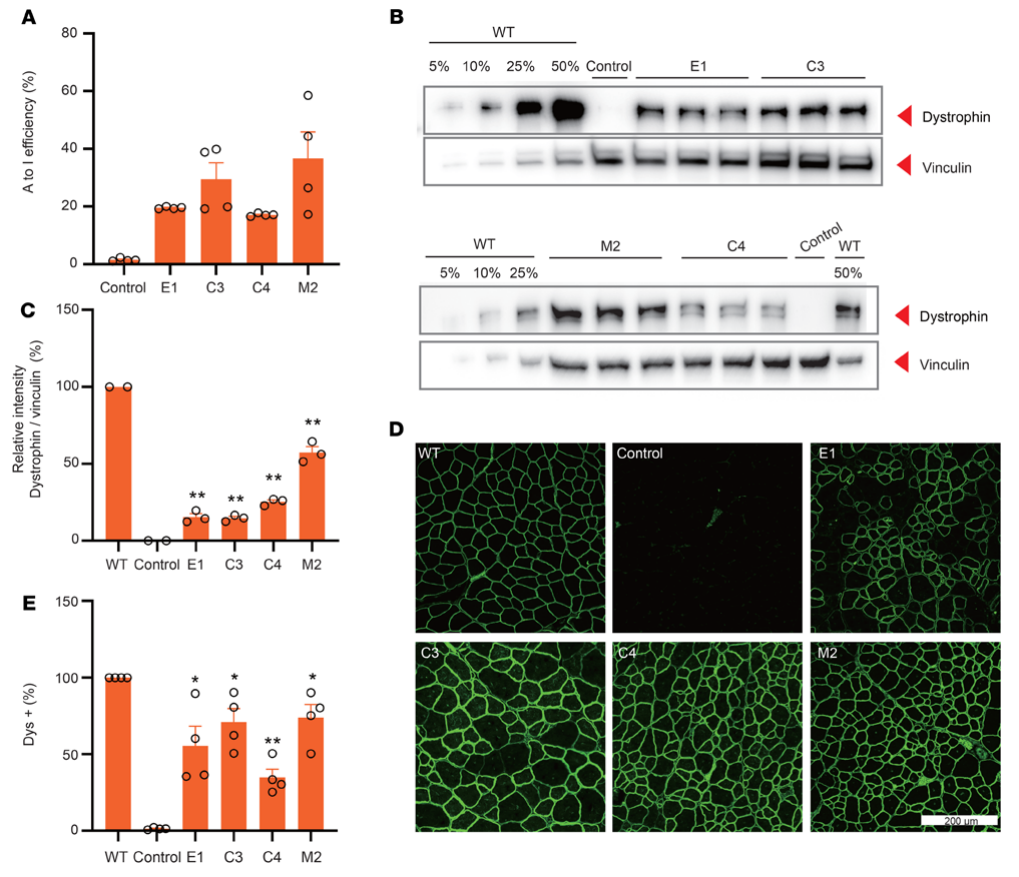
AAV-mxABE robustly rescues dystrophin expression in TA 6 weeks after injection. (**A**) Deep sequencing of in vivo RNA editing 6 weeks after i.m. injection with AAV9-E1, -C3, -C4, and -M2 constructs (*n* = 4). (**B**) Western blot analysis of dystrophin (MilliporeSigma, D8168) protein expression in TA muscles of WT and DMD^E30mut^ mice. Intramuscular injection of saline in the DMD^E30mut^ mice was the control. Vinculin was used as the loading control. (**C**) Quantification of dystrophin expression from Western blots after normalization to vinculin (*n* = 3). (**D**) Immunohistochemistry of dystrophin in TA muscles 6 weeks after i.m. injection with different AAV9 constructs. Dystrophin (Abcam, ab15277) is indicated in green. Scale bar: 200 μm. (**E**) Quantification of Dys^+^ fibers in cross sections of TA muscles (*n* = 4). Dots and bars represent biological replicates and are mean ± SEM. Unpaired 2-tailed Student’s *t* test. *P < 0.05, **P < 0.01 vs. control.

To evaluate the long-term effects of EFS-, CBh-, and MHCK7-mxABEs in DMD^E30mut^ mice, RNA editing rates, protein expression, and histological analyses were conducted in TA tissue 6 weeks after treatment. Editing rates were approximately 40% lower (25.69% ± 3.65%) 6 weeks after treatment compared with the 3-week values (Figure 4A and Supplemental Figure 9, A and B). Also, we found that base editor mRNA levels were reduced in the mxABE-treated muscles after 6 weeks compared with after 3 weeks (Supplemental Figure 9C), potentially owing to dilution of the AAV titer in rapidly growing muscle, which aligns well with results from another study (13). Intriguingly, dystrophin staining showed that the Dys^+^ area was similar 6 weeks compared with 3 weeks after mxABE treatment (Figure 4, B and C, and Supplemental Figure 10), indicating a sustained therapeutic effect. In addition, quantification of Western blot bands from TA lysates of mxABE-injected DMD^E30mut^ and WT mice indicated that the M2 vector expressing MHCK7-mxABE induced the highest level of dystrophin protein expression, which was around 50% of WT levels (Figure 4, D and E). These results suggest that the mxABE vectors can provide sustained and proliferation-tolerant therapeutic effects, despite the rapid growth of muscle fiber and the reversibility of RNA editing.

In addition, to investigate off-target effects of mxABE in vivo, we collected RNA from CBh-mxABE–, MHCK7-mxABE–, and saline-injected TA tissue for RNA-Seq and SNV analysis. CBh- and MHCK7-mxABE treatment induced a similar number of SNVs compared with the saline control (Supplemental Figures 11 and 12), indicating that these reagents are highly specific for the target sequence and the delivery vector is highly efficient.

### Systemic delivery of AAV-mxABE restores dystrophin expression and muscle function in multiple tissues.

**Figure 5:**
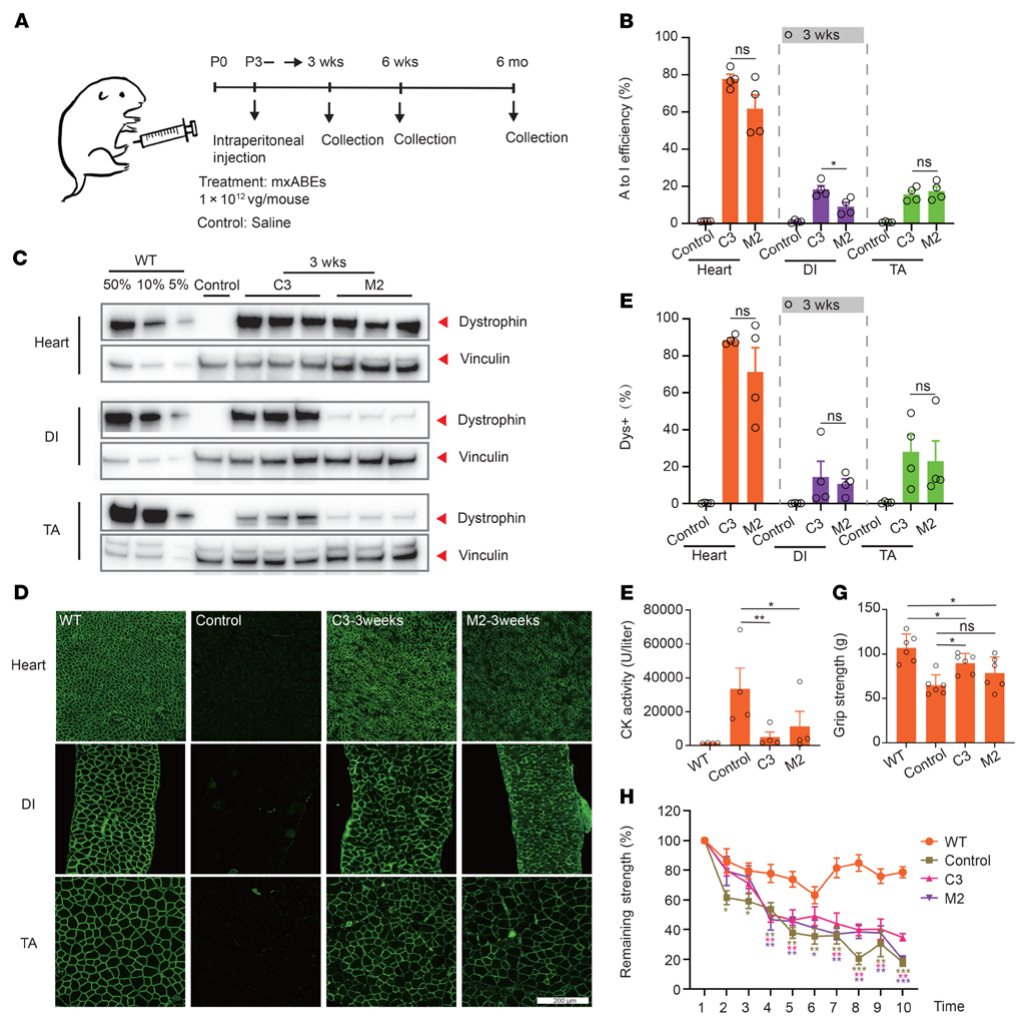
Systemic delivery of AAV-mxABE rescues dystrophin expression and muscle function in multiple organs after 3 weeks. (**A**) Schematic of systemic administration of AAV particles. AAV9-C3 and AAV9-M2 particles were injected intraperitoneally (i.p.) into postnatal day 3 (P3) DMD^E30mut^ mice. Some DMD^E30mut^ mice were injected with saline as mock-treated controls. Arrows indicate time points for tissue collection after i.p. injection. (**B**) Measurement by deep sequencing of dystrophin transcripts of the targeted A-to-I editing efficiency in TA, DI, and heart after systemic delivery (*n* = 4). (**C**) Western blot analysis shows restoration of dystrophin expression in the TA, DI, and heart of DMD^E30mut^ mice 3 weeks after injection. Dilutions of protein extract from WT mice were used to standardize dystrophin expression (5%, 10%, and 50%). Vinculin (Cell Signaling Technology, 13901S) was used as the loading control. (**D**) Immunohistochemistry for dystrophin in TA, DI, and heart of DMD^E30mut^ mice was performed 3 weeks after systemic injection. Dystrophin (Abcam, ab15277) is shown in green. Scale bar: 200 μm. (**E**) Quantification of Dys^+^ in cross sections of TA, DI, and heart muscles (*n* = 4). (**F**) CK levels were measured in WT, DMD^E30mut^ mock-treated, and DMD^E30mut^ AAV9-mxABE–treated mice 3 weeks after injection (*n* = 4). (**G**) Forelimb grip strength was measured in WT mice, DMD^E30mut^ mice, and DMD^E30mut^ mice treated with AAV9-C3/M2 particles (*n* = 6). (**H**) The remaining strength was also measured during 10 repetitions at 10-second intervals (*n* = 6). Dots and bars represent biological replicates and are mean ± SEM. Different asterisks represent statistical significance (*P* < 0.01) in multiple-comparison test using ANOVA.

Having demonstrated that local injection of mxABE robustly restored dystrophin expression in TA muscle, we next examined the effects of systemic delivery of AAV-mxABE in DMD^E30mut^ mice. For systemic delivery, we intraperitoneally injected 1 × 10^12^ vg viral vehicles of all 10 mxABE constructs or control saline into neonatal DMD^E30mut^ mice 3 days after birth (P3) (Figure 5A). We collected TA, DI, and heart tissue for analysis at different time points (Figure 5A). Three weeks after injection, deep-sequencing analysis indicated that C3-mxABE and M2-mxABE were remarkably efficient RNA base editors in heart tissue (77% and 61% on average, respectively, after 3 weeks), but their RNA base editing efficiencies were lower in DI and TA (Figure 5B). Between the two, C3-mxABE had generally higher or at least comparable base editing efficiency relative to M2-mxABE in all 3 tissues (Figure 5B and Supplemental Figure 13).

To investigate possible reasons underlying the observed differences in correction efficiency among heart, DI, and TA tissues, we performed quantitative PCR (qPCR) to quantify mxABE expression in each tissue. We found that, regardless of whether constitutive or tissue-specific promoters were used, expression was highest in heart tissue (Supplemental Figure 14A), which suggested that the higher mxABE activity in heart was due to either stronger mxABE expression or more efficient vehicle delivery to heart tissue compared with DI and TA tissues. Subsequent quantitative Western analysis showed that dystrophin protein was most abundant in heart tissue compared with DI and TA (Figure 5C and Supplemental Figure 16B), which was consistent with the respective base editing rates in these tissues. Immunohistochemical analysis uncovered an extensive region of Dys^+^ muscle fibers in the heart, DI, and TA of mxABE-treated DMD^E30mut^ mice, whereas Dys^+^ tissue was completely absent in the saline-treated controls (Figure 5, D and E, and Supplemental Figure 15). Furthermore, H&E and Sirius red staining showed that histopathological hallmarks of muscular dystrophy, such as fibrosis, necrotic myofibers, and regenerated fibers with central nuclei, were substantially alleviated in the TA, DI, and heart muscles 3 weeks after AAV9-mxABE delivery compared with saline-treated control tissues (Supplemental Figure 16). Compared with untreated DMD^E30mut^ mice, serum CK levels in CBh(C3)-mxABE– and MHCK7(M2)-mxABE–treated DMD^E30mut^ mice were significantly reduced by 87.29% and 71.25%, respectively (Figure 5F).

Finally, we examined whether mxABE could restore muscle function by measuring forelimb grip strength in mxABE-treated and untreated DMD^E30mut^ mice as well as WT mice. Untreated DMD^E30mut^ mice exhibited approximately 40% less forelimb grip strength 3 weeks after birth compared with WT mice (Figure 5G). Notably, 3 weeks after treatment, forelimb grip strength was significantly restored in DMD^E30mut^ mice treated with CBh(C3)-mxABE compared with saline-treated DMD^E30mut^ mice, whereas the improvements in grip strength in DMD^E30mut^ mice treated with MHCK7(M2)-mxABE were not statistically significant (Figure 5G). Repetitive forelimb grip tests showed that grip strength declined rapidly with increasing grip time in mxABE-treated DMD^E30mut^ mice compared with WT mice (Figure 5H), suggesting that muscle dysfunction was only partially alleviated in mxABE-treated DMD^E30mut^ mice; thus, further optimization of mxABE expression or delivery is necessary to enhance therapeutic effects.

**Figure 6:**
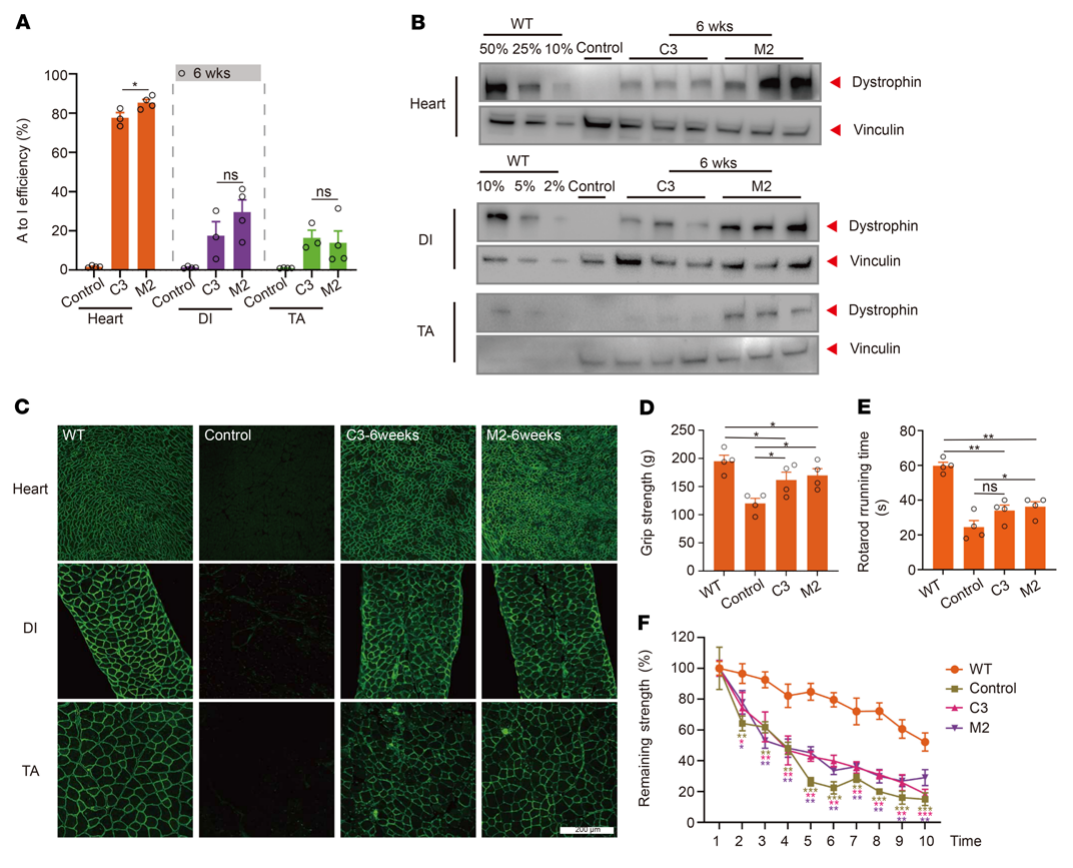
Restored dystrophin expression and muscle function 6 weeks after systemic mxABE treatment. (**A**) Base editing efficiency in heart, DI, and TA muscle 6 weeks after systemic treatment with mxABE. Unpaired Student’s *t* test (*n* = 4). (**B**) Western blot analysis of dystrophin (MilliporeSigma, D8168) restoration in heart, DI, and TA. (**C**) Histological immunostaining analysis of Dys^+^ (Abcam, ab15277) muscle area in heart, DI, and TA after treatment. Scale bar: 200 μm. (**D**) Forelimb grip strength test results for WT, untreated, and M3- and C1-treated DMD^E30mut^ mice. Unpaired Student’s *t* test (*n* = 4). (**E**) Exhausted strength test showed partial rescue of forelimb muscle function in mxABE-treated DMD^E30mut^ mice. Different asterisks represent statistical significance (*P* < 0.01) in multiple-comparison test using ANOVA (*n* = 4). (**F**) Rotarod running test indicated functional muscle improvement in mxABE-treated DMD^E30mut^ mice (*n* = 4). Dots and bars represent biological replicates and are mean ± SEM. Significance is indicated by asterisks.

At 6 weeks, we also checked A-to-I base editing efficiency, dystrophin restoration level, and grip strength in mxABE-treated and untreated DMD^E30mut^ mice (Figure 5A). The RNA editing rate 6 weeks after systemic mxABE administration was still as high as 85% in the heart, but only 29% in TA and 16% in DI (Figure 6A and Supplemental Figure 17), which might be because of different mxABE expression levels among the 3 tissues (Supplemental Figure 18A). Both Western analysis and immunostaining confirmed that dystrophin expression was sustained in all 3 muscle tissues after 6 weeks (Figure 6, B and C; Supplemental Figure 18, B and C; and Supplemental Figure 19). C3-mxABE–treated mice had decreased dystrophin levels compared with M2-mxABE–treated mice after 6 weeks, which is consistent with what we observed after 3 weeks (Figures 5 and 6). Forelimb grip strength of both C3- and M2-mxABE–treated DMD^E30mut^ mice was significantly improved (Figure 6D), and both groups of mxABE-treated DMD^E30mut^ mice also had significantly increased running time in the rotarod test compared with untreated mice (Figure 6E), suggesting improved muscle function. Finally, similar to results 3 weeks after treatment, 6 weeks after treatment, mxABE only partially restored performance in a repetitive grip test (Figure 6F).

**Figure 7:**
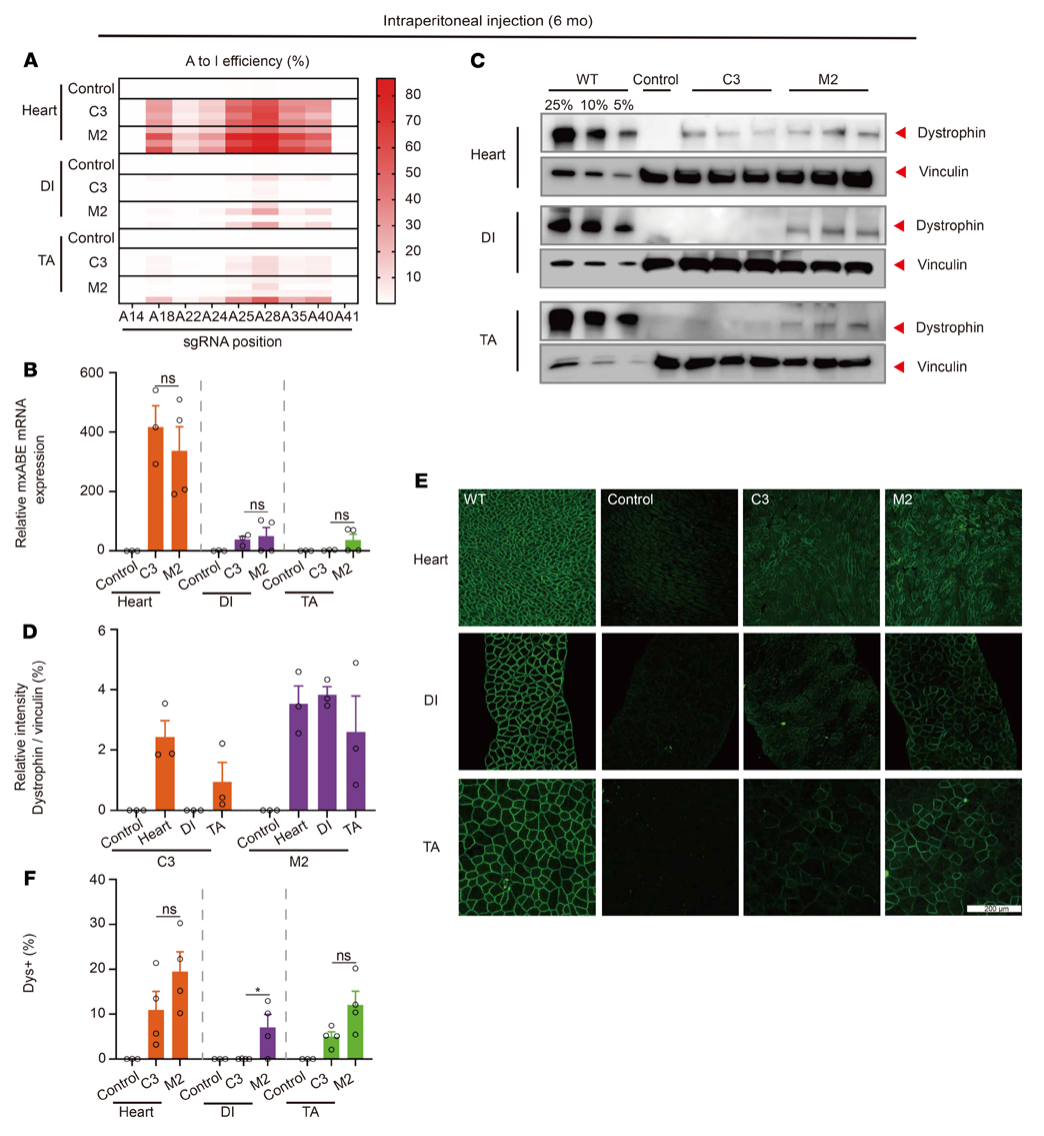
Systemic AAV-mxABE delivery sustains dystrophin restoration after 6 months. (**A**) Heatmap of base editing rate in heart, DI, and TA muscle 6 months after treatment with AAV-mxABE (*n* = 4). (**B**) mxABE expression level in heart, DI, and TA 6 months after systemic injection (*n* = 4). (**C**) Western blot analysis of dystrophin (MilliporeSigma, D8168) restoration in different muscle tissues 6 months after treatment. (**D**) Quantification of Western blot results in **C**. (**E**) Histological immunostaining analysis of Dys^+^ (Abcam, ab15277) muscle area in heart, DI, and TA 6 months after treatment (*n* = 3). Scale bar: 200 μm. (**F**) Quantification of immunostaining results in **E** (*n* = 4). Dots and bars represent biological replicates and are mean ± SEM. Unpaired Student’s *t* test. Significance is indicated by an asterisk.

To examine the long-term therapeutic effect of mxABE treatment, DMD^E30mut^ mice with or without mxABE administration were monitored for 6 months and then euthanized to analyze muscle tissue (Figure 5A). RNA base editing rate was up to 80% in heart muscle 6 months after treatment, but it was only 10% in DI and TA muscle (Figure 7A), which is in agreement with our findings in shorter-term studies (Figure 7B). Western analysis confirmed detectable dystrophin expression in heart, TA, and DI 6 months after treatment, but protein levels were dramatically decreased in comparison with earlier time points (Figure 7, C and D). We also quantified Dys^+^ muscle using immunostaining, which showed that only about 20% of muscle area maintained dystrophin expression in heart muscle, and even less Dys^+^ muscle was observed in DI and TA (Figure 7, E and F, and Supplemental Figure 20). In addition, collagen staining of heart tissue from treated and untreated DMD^E30mut^ mice as well as WT mice was conducted to detect cardiac fibrosis, and this showed no differences among the 3 cohorts (Supplemental Figure 21), which is consistent with previous findings showing that most mdx mice have less severe and slower cardiac progression compared with human patients (14–16). To measure the host immune response to AAV-mxABE treatment, IL-2, IL-15, and IL-18 activation was examined by qPCR at baseline and 3 weeks, 6 weeks, and 6 months after treatment. Expression of all 3 cytokines was substantially increased 6 weeks after treatment but returned to basal levels by 6 months after treatment (Supplemental Figure 22, A–C). Furthermore, we examined potential toxicity of mxABE administration by analyzing alanine transaminase, total protein, and urea. All 3 serum biomarkers were normal before and after AAV-mxABE treatment (Supplemental Figure 22, D–F), indicating that AAV-mxABE administration is relatively safe. Overall, mxABE was effective at restoring dystrophin expression in DMD^E30mut^ mice for up to 6 months after systemic administration of mxABE via AAV.

DMD is usually diagnosed in patients when they are around 5 years old. To closely mimic the clinical scenario, we performed systemic injection of AAV-mxABE in 8-week old mice and investigated treatment outcomes. Both intravenous (AIV group) and intraperitoneal (AIP group) injection delivering 1 × 10^12^ vg of AAV particles with mxABE-M2 per mouse was tested (Supplemental Figure 23A). We collected heart, DI, and TA muscle 6 weeks after injection for analysis (Supplemental Figure 23A). In heart muscle, the RNA base editing rate was up to 49%, whereas it was only 6% in DI and TA muscle (Supplemental Figure 23, B and C). Western analysis only detected dystrophin expression in heart tissues, not in DI or TA (Supplemental Figure 23D). However, immunostaining showed that mxABE treatment of adult DMD^E30mut^ mice restored Dys^+^ muscle in heart, DI, and TA to up to 38% of WT levels (Supplemental Figure 23, E and F, and Supplemental Figure 24). In the forelimb grip strength test, no significant improvement in muscle function was observed 6 weeks after mxABE injection in adult DMD^E30mut^ mice (Supplemental Figure 23G), implying that an optimized approach is required to potentially functionally rescue adult DMD^E30mut^ models.

Overall, our findings demonstrate that systemic delivery of AAV-mxABE can rescue *DMD* gene expression, and support further exploration of this approach as a potential gene therapy to treat patients with DMD and other monogenic diseases.

## Discussion

PTCs resulting from point mutations account for 10% of the lesions leading to DMD (17); thus, strategies that induce PTC read-through for all 3 stop codons (UAG, UAA, and UGA) hold great potential as a therapeutic strategy to treat DMD caused by nonsense mutations. Here, we demonstrated that the compact mxABE RNA base editor can mediate the correction of several pathogenic nonsense mutations in *DMD* transcripts with sufficient efficiency to stably restore dystrophin expression in skeletal, DI, and heart muscle in humanized DMD^E30mut^ mice harboring the causative genetic lesion detected in an 8-year-old patient with DMD. In particular, our mxABE approach rescued dystrophin expression in DMD^E30mut^ mice to up to 50% of WT levels, which reflects a 16-fold improvement compared with the 3% of dystrophin restoration recently achieved using an MS2-derived RNA base editor delivered by local muscle injection (12). In addition, systemic administration of mxABE in the present study also induced robust restoration of dystrophin expression in multiple tissues, which, so far, has not been demonstrated using other RNA base editors. Therefore, our findings serve as a proof of concept for RNA base editing as a strategy for DMD treatment.

Nonsense point mutations have been identified as causative factors in about 10% of all monogenic diseases (2). Our results suggest that mxABE-mediated PTC read-through may be used to effectively treat other hereditary diseases induced by nonsense point mutations. Moreover, we found that systemic administration of mxABE resulted in greater and more durable rescue of dystrophin expression in heart tissue compared with skeletal muscle in mice, suggesting that this approach may be particularly effective to reduce the likelihood of poor outcomes or mortality in patients with DMD or other monogenic diseases that put them at high risk of cardiac failure.

mxABE driven by the muscle-specific MHCK7 promoter produced a slightly lower base editing rate and level of dystrophin restoration compared with expression under the control of a ubiquitous CBh promoter, which might be due to slightly weaker activity of MHCK7 compared with CBh. Intriguingly, dystrophin restoration in skeletal muscle after mxABE treatment progressively decreased approximately 6 weeks after treatment, but this decrease was less pronounced in heart tissue. Because mxABE is correcting RNA rather than DNA, sustainable RNA editing activity and therapeutic effect would rely on the long-lasting expression and editing activity of mxABE in vivo. Previous studies have shown low tropism of AAV for satellite cells (13, 18) and preexisting immunity against CRISPR reagents (19, 20), possibly leading to progressive loss of mxABE and decreased dystrophin restoration in skeletal muscles over time. These results suggest that mxABE may be more suitable as a therapeutic intervention for nondividing tissue such as the eye, neurons, and cardiomyocytes than for rapidly dividing tissue like skeletal muscle.

RNA base editors induce A-to-G or C-to-U conversion in transcripts through deaminase activity of ADAR proteins acting on adenine or cytosine nucleobases near the targeted sequence. This activity may also result in off-target, unintended bystander base editing, and therefore monitoring and controlling for bystander effects is an essential step in the development of effective base editors. Notably, bystander effects have also proven to be a significant limitation of the most efficient previously described RNA base editors (5–7). In this work, the mxABE editor showed A-to-G editing rates of up to 84% in vivo, converting nonsense mutant UAG stop codons to UGG in *DMD* mRNA transcripts. However, bystander A-to-G conversions in sequences adjacent to the disease-causing mutation were consistently detected. Although we did not detect introduction of any new PTCs through bystander A-to-G editing, such aggressive editing activity could foreseeably cause other pathogenic amino acid substitutions. To address this long-standing issue in base editing, another recent study described a gRNA design strategy that employs U deletion to minimize bystander editing effects in ADAR proteins (21), which could enhance the specificity of PTC correction for RNA base editors, and hence increase the safety of candidate base editing gene therapies.

In addition to analyzing bystander effects, we also analyzed the transcriptome of mice treated with AAV-mxABE, which showed no statistically significant difference in the number of SNVs between treated and untreated mice, suggesting that the off-target effects were limited to sequences in the immediate proximity of the target sequence. As a strategy to potentially reduce off-target effects, especially in nontarget tissue, we evaluated the efficiency of tissue-specific promoters to drive mxABE expression. Our results showed that these promoters can rescue dystrophin expression in muscle tissues to levels comparable to those achieved by ubiquitous promoters.

We validated a pathogenic nonsense mutation identified in a patient with DMD admitted to our clinic and established a humanized mouse model of DMD to study the efficiency of RNA base editing to rescue DMD-related phenotypes. Using the compact mxABE base editor packaged in AAV, we demonstrated that dystrophin expression can be restored in multiple muscle tissues through local or systemic administration, indicating the potential of this system to treat monogenic diseases resulting from PTCs.

## Methods

### Plasmid construction.

The pU6-BpiI-EFS-Cas13X.1 plasmid encodes a human codon-optimized Cas13X.1 driven by the EFS promoter and a U6-driven CRISPR RNA with a BpiI cloning site. The sgRNAs (including missense mutations in exons 23, 30, 54) were designed using the CHOPCHOP tool (http://chopchop.cbu.uib.no), then synthesized as DNA oligonucleotides and cloned into pU6-BpiI-EFS-Cas13X.1 to form the CRISPR targeting plasmids (listed in Supplemental Table 1). A reporter vector was constructed with mCherry and ATG-removed GFP as well as the mutant sequence identified in the patient with DMD (c.4174C>T mutation). The high-efficiency EFS promoter in the sgRNA plasmid for *DMD* gene exon 30 was replaced with the CBh or the MHCK7 promoter using the Gibson assembly method (New England Biolabs), and SV40 NLS was also replaced with NP NLS or BP NLS, to construct different AAV9 vectors. All AAV9 vectors used in this study are listed in Supplemental Figure 2 and Supplemental Sequences.

### Cell culture, transfection, and flow cytometry analysis.

HEK293T cells from the ATCC were maintained in DMEM (Gibco, 11965092) supplemented with 10% FBS at 37°C and 5% CO_2_ in a humidified incubator. For sgRNA screening, CRISPR targeting plasmids and reporters were cotransfected using polyethylenimine transfection reagent. After transfected cells were cultured for 48 hours, we carefully resuspended the cell pellet and then analyzed or sorted cells using a FACSAria II (BD Biosciences). Flow cytometry results were analyzed with FlowJo X (v10.0.7).

### Generation of DMD^E30mut^ mice.

Mice were housed in a barrier facility with a 12-hour light/12-hour dark cycle and maintained in accordance with the Instructive Notions with Respect to Caring for Laboratory Animals issued by the Ministry of Science and Technology of China. DMDE^30mut^ mice were generated in the C57BL/6J background using the CRISPR/Cas9 system. In brief, 2 sgRNAs targeting mouse *Dmd* exon 30 were designed (Supplemental Table 1), and then the T7 promoter sequence was added to the sgRNA template. After PCR product was purified directly using an Omega gel extraction kit (Omega, D2500-02), templates were used for in vitro transcription using the MEGAshortscript T7 Kit (Invitrogen, AM1354). sgRNAs were purified using a MEGAclear Kit (Invitrogen, AM1908) and eluted with nuclease-free water. The concentration of target sgRNA was measured using a NanoDrop instrument (NanoPhotometers NP80, IMPLEN). For cytoplasmic injection, spCas9 mRNA (100 ng/μL), sgRNA (100 ng/μL), and HMEJ donor (100 ng/μL) were mixed and then injected into fertilized eggs using a FemtoJet microinjector (Eppendorf) with constant flow settings. The injected zygotes were cultured in KSOM medium (EMD Millipore Corporation) for 12 hours and surgically transferred to the oviduct of recipient mice 24 hours after estrus was observed. Genomic DNA from the tail tissue of founder (F_0_) mice was isolated according to the manufacturer’s instructions for the Omega kit (Omega, D3396-02) for PCR (Supplemental Table 1), followed by gel electrophoresis.

### AAV9 production and delivery to DMD^E30mut^ mice.

AAVs were produced using PackGene Biotech (Guangzhou, China) and purified by iodixanol density gradient centrifugation. For intramuscular injection, DMDE^30mut^ mice were anesthetized, and TA muscle was injected with 50 μL of AAV9 (3 × 10^11^ vg) preparations or with the same volume saline solution. For intraperitoneal injection, the P3 DMDE^30mut^ mice were injected using an ultrafine needle (31 gauge) with 50 μL of AAV9 (1 × 10^12^ vg) preparations or with saline solution.

### Targeted deep sequencing.

To analyze A-to-I base editing efficiency, the RNA of successfully transfected cells or of AAV9-mxABE–treated tissues was isolated with RNA Easy isolation reagent (Vazyme) according to the manufacturer’s protocol. cDNA was synthesized using a HiScript II One Step RT-PCR Kit (Vazyme, P611-01) following the manufacturer’s protocol and amplified with Phanta Max Super-Fidelity DNA polymerase (Vazyme, P505-d1) for Sanger or deep-sequencing methods. Deep-sequencing libraries were used to add Illumina flow cell binding sequences and specific barcodes on the 5′ and 3′ ends of primer sequences. The products were pooled and sequenced with 150 paired-end reads on an Illumina HiSeq instrument. FASTQ format data were analyzed using the Cutadapt (version v2.8) (22) according to assigned barcode sequences.

### qPCR and RNA sequencing.

Total mRNA was extracted from muscle tissue, and cDNA was synthesized using a HiScript II One Step RT-PCR Kit (Vazyme, P611-01) following the manufacturer’s protocol. Then each 20 μL PCR reaction contained approximately 2 μL cDNA, 0.25 μM of each forward and reverse primer, and 10 μL of AceQ Universal SYBR qPCR Master Mix (Vazyme, Q511-02). Amplification was performed on a Roche 480 II-A using the following program: initial hold for 5 minutes, then 45 cycles of 95°C for 10 seconds, 60°C for 10 seconds, and 72°C for 10 seconds. qPCR results were calculated by normalizing to GAPDH mRNA level.

To analyze the specificity of optimal AAV9 vectors, RNAs of cells or muscles were extracted with TRIzol reagent (Invitrogen, 15596026) and sent to Azenta Life Sciences (Suzhou, China) for transcriptome sequencing. Transcriptome sequencing results were analyzed as previously described and presented as the mean of all repeats.

### Western blot analysis.

Samples were homogenized with RIPA buffer supplemented with protease inhibitor cocktail. Lysate supernatants were quantified using a Pierce BCA protein assay kit (Thermo Fisher Scientific, 23225) and adjusted to an identical concentration using H_2_O. Equal amounts of sample were mixed with NuPAGE LDS sample buffer (Invitrogen, NP0007) and 10% β-mercaptoethanol and then boiled at 70°C for 10 minutes. Ten micrograms total protein per lane was loaded into 3%–8% Tris-acetate gels (Invitrogen, EA03752BOX) and electrophoresed for 1 hour at 200 V. Protein was transferred onto a PVDF membrane under wet conditions at 350 mA for 3.5 hours. The membrane was blocked in 5% nonfat milk in TBST buffer and then incubated with primary antibody to label the specific protein. After washing 3 times with TBST, the membrane was incubated with HRP-conjugated secondary antibody specific to the IgG of the species of primary antibody against dystrophin (MilliporeSigma, D8168) or vinculin (Cell Signaling Technology, 13901S). The target proteins were visualized using chemiluminescent substrates (Invitrogen, WP20005).

### Histology and immunofluorescence.

Tissues were collected and put into preconditioned 4% paraformaldehyde. The fixed tissues were dehydrated from low to high concentrations of alcohol. After xylene treatment, tissues were placed in melted paraffin wax, cut into 10 μm slices, and attached to slides. Xylene was used to wash the paraffin; then slides were passed through high-concentration to low-concentration alcohol, and finally put into distilled water. For H&E staining, slides were stained with hematoxylin for 3–8 minutes followed by color separation using acid water and ammonia water. After dehydration using 70% and 90% alcohol for 10 minutes each, tissues were stained in eosin staining solution for 1–3 minutes, and dehydrated in ascending alcohol solutions (50%, 70%, 80%, 95%, 100%). Coverslips were mounted onto the section on glass slides with neutral resin.

For Sirius red staining, slides were stained with Picrosirius red for one hour and washed in 2 changes of acidified water. Physical removal of most of the water from the slides was accomplished by vigorous shaking. Then, slides were dehydrated in 3 changes of 100% ethanol, cleared in xylene, and finally mounted in neutral resin.

For immunofluorescence, tissues were mounted in OCT compound and snap-frozen in liquid nitrogen. Serial frozen cryosections (10 μm) were fixed for 2 hours at 37°C followed by permeabilization with PBS plus 0.4% Triton X for 30 minutes. After washing with PBS, samples were blocked with 10% goat serum for 1 hour at room temperature. Next, the slides were incubated overnight at 4°C with primary antibodies against dystrophin (Abcam, ab15277) and spectrin (Millipore, MAB1622). The next day, samples were washed extensively with PBS and incubated with compatible secondary antibodies (Alexa Fluor 488 AffiniPure donkey anti-rabbit IgG, Jackson ImmunoResearch Laboratories, 711-545-152; or Alexa Fluor 647 AffiniPure donkey anti-mouse IgG, Jackson ImmunoResearch Laboratories, 715-605-151) and DAPI for 3 hours at room temperature. Samples were washed for 15 minutes with PBS, and slides were sealed with Fluoromount-G mounting medium (SouthernBiotech). All images were visualized using an Olympus FV3000 or Nikon C2 microscope. The number of Dys^+^ muscle fibers is represented as a percentage of total spectrin-positive muscle fibers.

### Forelimb grip strength test and rotarod test.

Muscle strength was assessed at various time points. Briefly, mice were removed from the cage, weighed, and held from the tip of the tail, causing the forelimbs to grasp the pull-bar assembly connected to the 47200 Grip Strength Meter (Ugo Basile). The mouse was drawn along a straight line leading away from the sensor until the mouse could no longer grasp the gridiron, and the peak amount of force in grams was recorded. The assessment was repeated 10 times with 10-second intervals between.

For the rotarod test, the week before the experiment, we performed daily 30-minute training trials. During the test, mice were trained on the rotarod (Ugo Basile Inc.) with accelerating speed from 4 to 40 rpm over 30 seconds. Four mice were tested simultaneously on the rotarod, and the test was repeated 5 times per animal. Latency was measured as the time from the beginning of the trial (start of the accelerated rod rotation) until the mouse fell off and onto the lever that stops the timer.

### Serum CK.

To measure CK levels, a blood sample was collected in an Eppendorf tube via cardiac puncture under ketamine anesthesia before euthanasia. Samples were centrifuged at 3,000*g* for 10 minutes, and then the serum was collected. CK activity was measured with creatine kinase (CK10) reagent (Pointe Scientific, 23-666-208) according to the manufacturer’s instructions.

### Data availability.

RNA-Seq data were deposited in the NCBI’s Sequence Read Archive (SRA PRJNA830930 and PRJNA830863).

### Statistics.

The data are presented as mean ± SEM. Differences were assessed using unpaired 2-tailed Student’s *t* test or 1-way ANOVA. Differences in means were considered statistically significant when they reached *P* less than 0.05. Significance levels are *P < 0.05, **P < 0.01, ***P < 0.001.

### Study approval.

The objectives of the present study were to generate a humanized DMD model and to obtain proof of concept for in vivo CRISPR-mediated RNA base editing in DMD. Male mice were used in all experiments, such as grip tests, CK analysis, and AAV9 injection. The numbers of independent biological replicates are given in the figure legends. All animal experiments were performed with the approval of the IACUC of the Institute of Neuroscience, Chinese Academy of Sciences, Shanghai, China. The patient study was approved by the ethics committee of First Affiliated Hospital of Fujian Medical University. Written informed consent was obtained from each subject to use their DNA for molecular analysis of the *DMD* gene and for further research. For the minor patient, parental consent for muscle biopsy and molecular analysis was obtained. The research was conducted according to the principles of the Declaration of Helsinki.

## Author contributions

HY, GL, and CX jointly conceived the project and designed experiments. HY, CX, WC, and NW supervised the project. GL, ZL, WY, LX, and EZ generated the humanized mouse model. GL and MJ designed vectors, performed in vitro experiments, and conducted confocal imaging. CX and XW assisted with construction of plasmids. GL and MJ performed in vivo virus injection, tissue dissection, histological immunostaining, and muscle function experiments. YL and QX analyzed RNA-Seq data. QX, JL, HW, and DY assisted with tissue dissection, immunostaining, and animal breeding. GL, MJ, ZL, QX, JL, DY, LS, and CX analyzed the data and organized figures. HY, CX, and WC wrote the manuscript with data contributed by all authors who participated in the project.

## Supplementary Material

Supplemental data

## Version 1 12/13/2022

In-Press Preview

## Version 2 02/01/2023

Electronic publication
