# Supplementary material for: Mini-dCas13X–mediated RNA editing restores dystrophin expression in a humanized mouse model of Duchenne muscular dystrophy
Source: J Clin Invest. 2023 Feb 1;133(3):e162809. doi: 10.1172/JCI162809 (PMC9888377; doi:10.1172/JCI162809)
Supplement: Supplemental data [file jci-133-162809-s211.pdf]

Supplementary materials for

# Mini-dCas13X-mediated RNA editing restores dystrophin expression in a humanized mouse model of Duchenne muscular dystrophy

Guoling Li[1,2†], Ming Jin[5†], Zhifang Li[3,6†], Qingquan Xiao[2†], Jiajia Lin[5†], Dong Yang[1,2†], Yuanhua Liu[2], Xing Wang[1], Long Xie[6], Wenqin Ying[2], Haoqiang Wang[1], Erwei Zuo[6], Linyu Shi[1], Ning Wang[5*], Wanjin Chen[5*], Chunlong Xu[3,4*], Hui Yang[1,2,4*]

**†These authors contributed equally to this work.**

***Corresponding author Email:**

ningwang@fjmu.edu.cn(N.W.), wanjinchen75@fjmu.edu.cn(W.C.), xucl@lglab.ac.cn(C.X.), huiyang@huidagene.com(H.Y.)

**This PDF file includes:**

Figure S1. In vitro studies of c.4174C>T mutation correction using the mxABE system.

Figure S2. Construction of 10 mxABE expression cassettes.

Figure S3. Representative Sanger sequencing trace of reporter transcripts.

Figure S4. Evaluation of SNV counts by transcriptome-wide off-target analysis *in vitro*.

Figure S5. Manhattan plots of transcriptome-wide off-target RNA editing analysis for different mxABE constructs.

Figure S6. mxABE-mediated A to I correction of other mutations in the *DMD* gene.

Figure S7. *In vivo DMD* gene editing by IM injection with mxABEs after 3 weeks.

Figure S8. Rescue of dystrophin expression following IM injection of mxABEs in DMD$^{E30mut}$ mice.

Figure S9. *In vivo DMD* gene editing by IM injection with mxABEs after 6 weeks.

Figure S10. Intramuscular AAV9 delivery of gene editing components rescues dystrophin expression.

Figure S11. Calculation of SNV counts by transcriptome-wide off-target analysis *in vivo*.

Figure S12. Manhattan plots of transcriptome-wide off-target RNA editing analysis *in vivo*.

Figure S13. Gene editing 3 weeks after systemic delivery of mxABEs in DMD$^{E30mut}$ mice.

Figure S14. AAV9-mxABE expression and dystrophin protein level after systemic delivery in DMD$^{E30mut}$ mice.

Figure S15. Histological analysis of dystrophin expression 3 weeks after systemic delivery of AAV9-mxABE in DMD$^{E30mut}$ mice.

Figure S16. Histological analysis 3 weeks after systemic delivery of AAV9-mxABE.

Figure S17. Base editing efficiency achieved by systemic delivery of mxABE after 6 weeks.
Figure S18. mxABE expression and dystrophin restoration level 6 weeks after systemic administration of AAV9-mxABE.
Figure S19. Immunostaining of dystrophin in heart, TA, and DI tissues 6 weeks after systemic administration of AAV-mxABE.
Figure S20. Immunostaining of dystrophin in heart, TA ,and DI 6 months after systemic administration of AAV-mxABE.
Figure S21. Collagen staining shows no obvious fibrosis in hearts of treated or untreated $DMD^{E30mut}$ mice 6 months after systemic administration of AAV-mxABE.
Figure S22. Host immune response to AAV-mxABE treatment indicates no overt toxicity after 6 months.
Figure S23. Systemic delivery of AAV-mxABE rescues dystrophin deficiency in adult $DMD^{E30mut}$ mice.
Figure S24. Immunostaining of dystrophin in adult $DMD^{E30mut}$ mice 6 weeks after systemic injection of AAV-mxABE.
Tables S1. Target sgRNA and primer sequences.
Supplemental Sequences

Figure S1

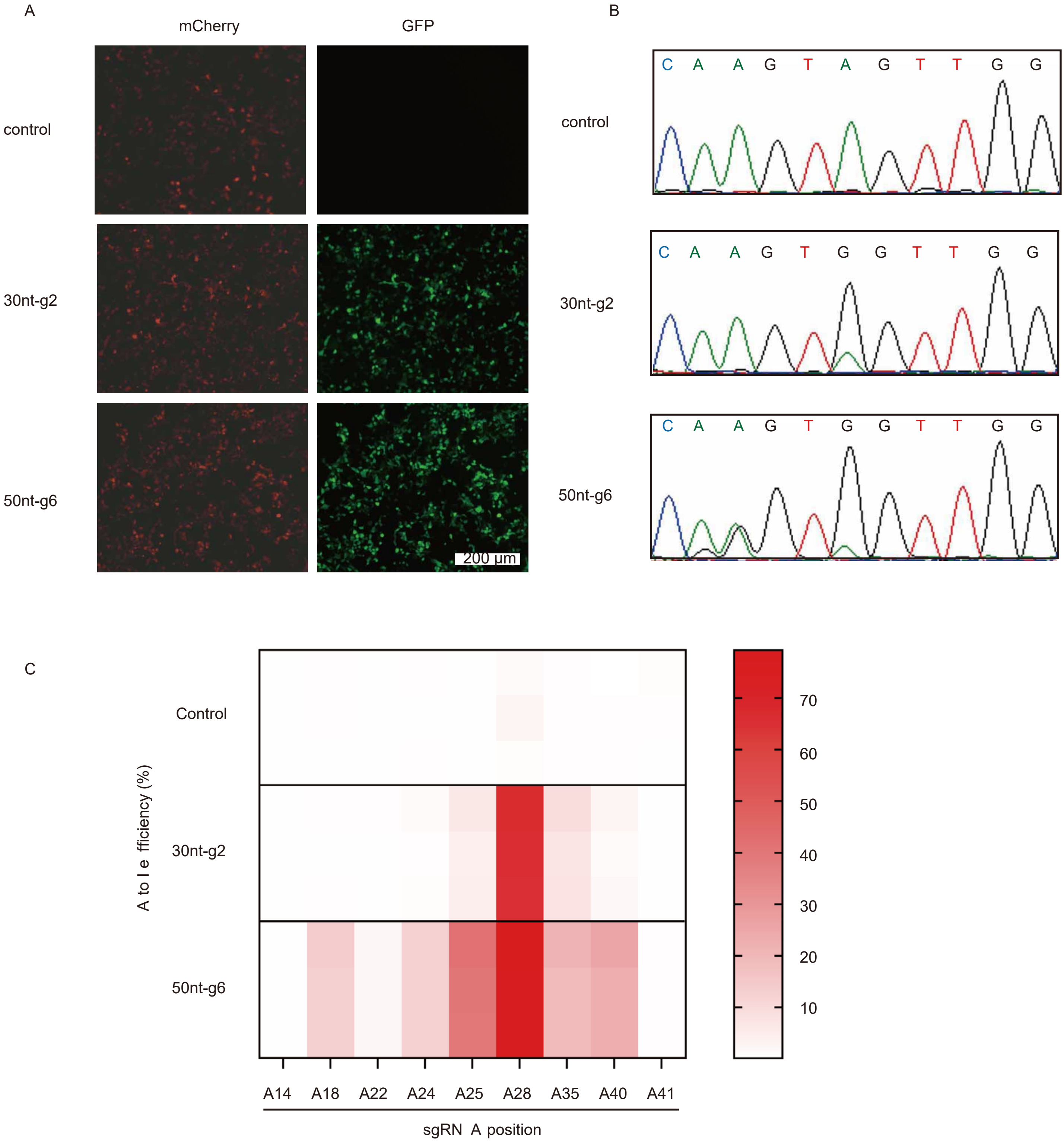
A
mCherry
GFP
control
30nt-g2
50nt-g6
200 μm
B
control
C A A G T A G T T G G
30nt-g2
C A A G T G G T T G G
50nt-g6
C A A G T G G T T G G
C
Control
30nt-g2
50nt-g6
A to I e fficiency (%)
70
60
50
40
30
20
10
A14 A18 A22 A24 A25 A28 A35 A40 A41
sgRN A position

## SUPPLEMENTAL FIGURES

**Figure S1. *In vitro* studies of c.4174C>T mutation correction using mxABE constructs.**

A. Fluorescence microscopy images of HEK293T cells transfected with reporter alone, or reporter and mxABE constructs. Scale bar, 200 µm. B. Representative Sanger sequencing trace of reporter transcripts. C. Measurements of the representative gRNA (30 nt-g2 and 50 nt-g6) effect on bystander A to I editing efficiency (n=3).

Figure S2

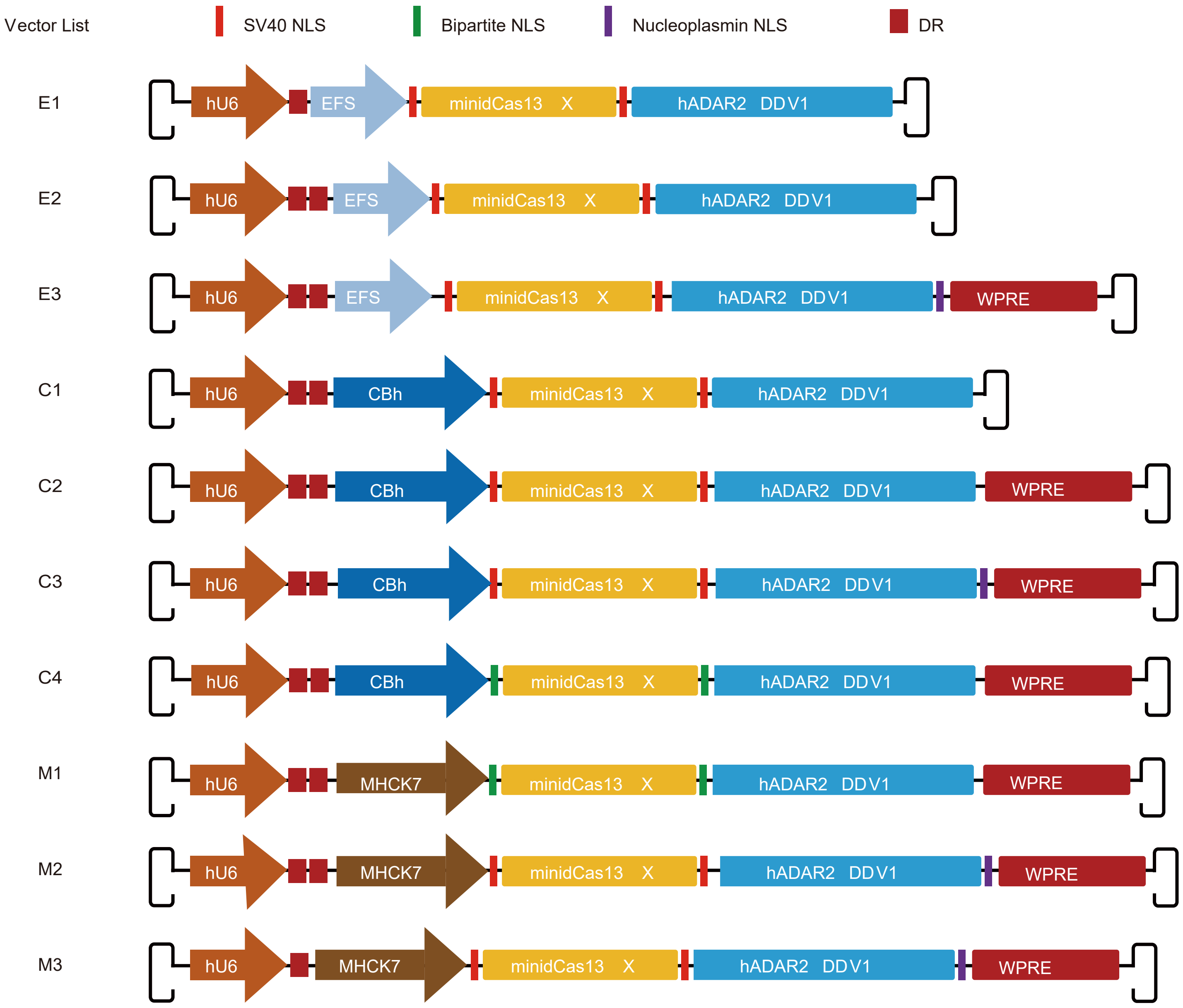
Vector List
SV40 NLS
Bipartite NLS
Nucleoplasmin NLS
DR
E1
hU6
EFS
minidCas13 X
hADAR2 DDV1
E2
hU6
EFS
minidCas13 X
hADAR2 DDV1
E3
hU6
EFS
minidCas13 X
hADAR2 DDV1
WPRE
C1
hU6
CBh
minidCas13 X
hADAR2 DDV1
C2
hU6
CBh
minidCas13 X
hADAR2 DDV1
WPRE
C3
hU6
CBh
minidCas13 X
hADAR2 DDV1
WPRE
C4
hU6
CBh
minidCas13 X
hADAR2 DDV1
WPRE
M1
hU6
MHCK7
minidCas13 X
hADAR2 DDV1
WPRE
M2
hU6
MHCK7
minidCas13 X
hADAR2 DDV1
WPRE
M3
hU6
MHCK7
minidCas13 X
hADAR2 DDV1
WPRE

**Figure S2. Construction of 10 mxABE expression cassettes.**

The mxABE expression cassettes contained different promoters (EFS, CBh, MHCK7), direct repeat (DR) numbers (single, dual DR), nuclear localization signals (NLS), and were constructed with or without a translational regulatory element (WPRE).

Figure S3

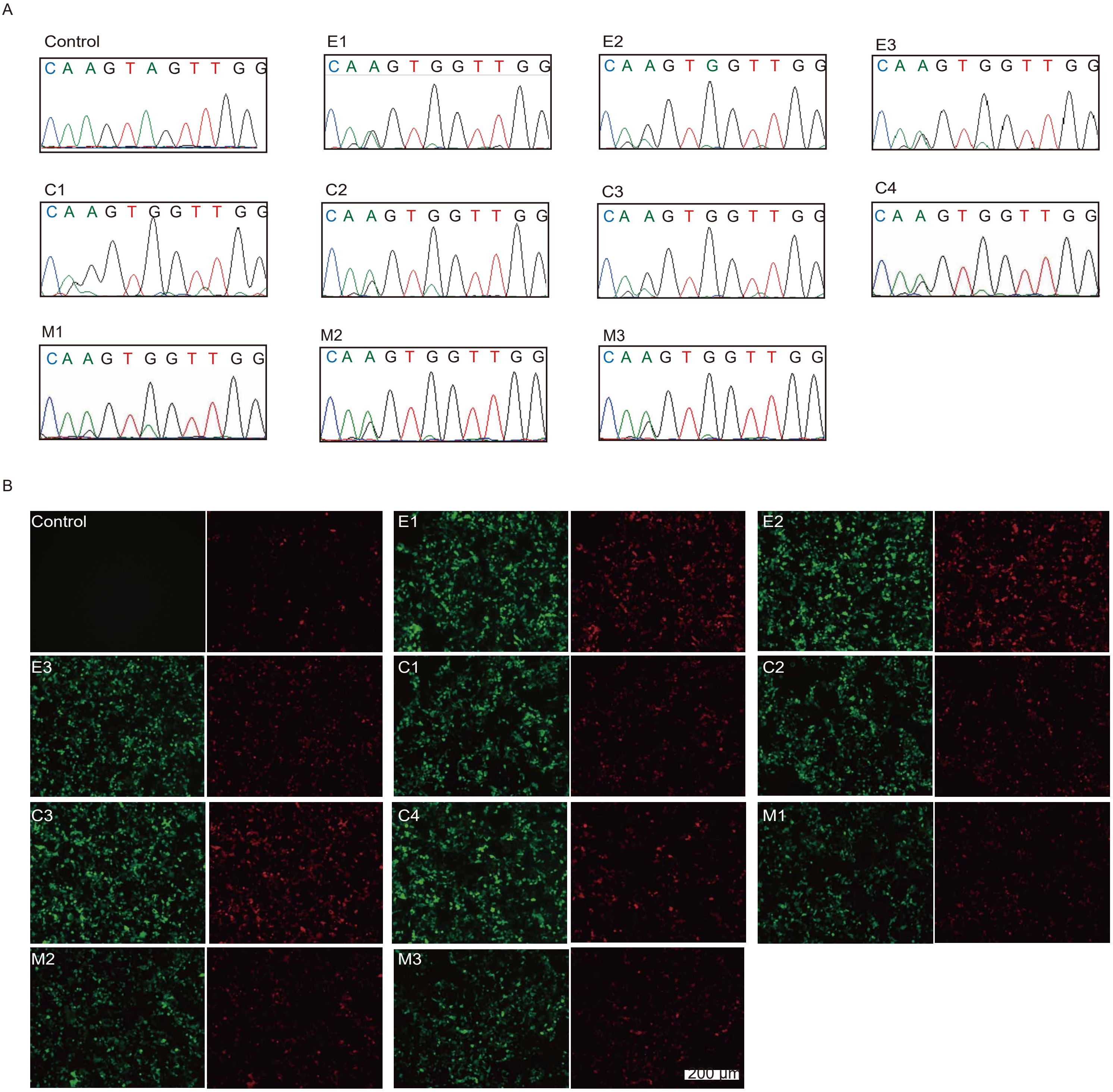
A
Control
C A A G T A G T T G G
E1
C A A G T G G T T G G
E2
C A A G T G G T T G G
E3
C A A G T G G T T G G
C1
C A A G T G G T T G G
C2
C A A G T G G T T G G
C3
C A A G T G G T T G G
C4
C A A G T G G T T G G
M1
C A A G T G G T T G G
M2
C A A G T G G T T G G
M3
C A A G T G G T T G G
B
Control
E1
E2
E3
C1
C2
C3
C4
M1
M2
M3
200 μm

**Figure S3. Representative Sanger sequencing trace of reporter transcripts.**

A. Sanger sequencing results showing representative A to I conversion on reporter transcripts by different mxABE editors. B. Fluorescence microscopy images of HEK293T cells transfected with reporter alone or with reporter and mxABE construct. Scale bar, 200 µm. Figure S3B and S1A showed similar results but from different experiments investigating the effect of gRNA length and construct expression elements on mutation correction efficiency respectively.

Figure S4

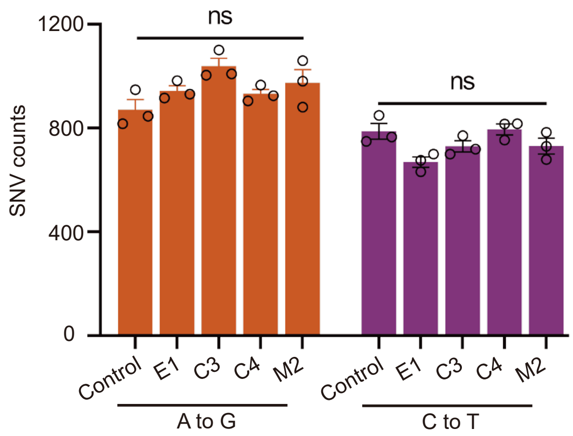

ns
ns
1200
800
400
0
SNV counts
Control
E1
C3
C4
M2
Control
E1
C3
C4
M2
A to G
C to T

**Figure S4. Evaluation of SNV counts *in vitro* using transcriptome-wide off-target analysis.**

The transcriptome-wide off-target effect of different mxABE constructs targeting the DMD c.4174C>T mutation were performed *in vitro*. The E1 mxABE construct was driven by an EFS promoter; C3 and C4 were driven by an CBh promoter; and M2 was driven by an MHCK7 promoter. Non-targeting mxABE construct was analyzed as a control. Values are shown as mean ± SEM (n=3). NS, not statistically significant in multiple comparison test using ANOVA (P< 0.01).

Figure S5

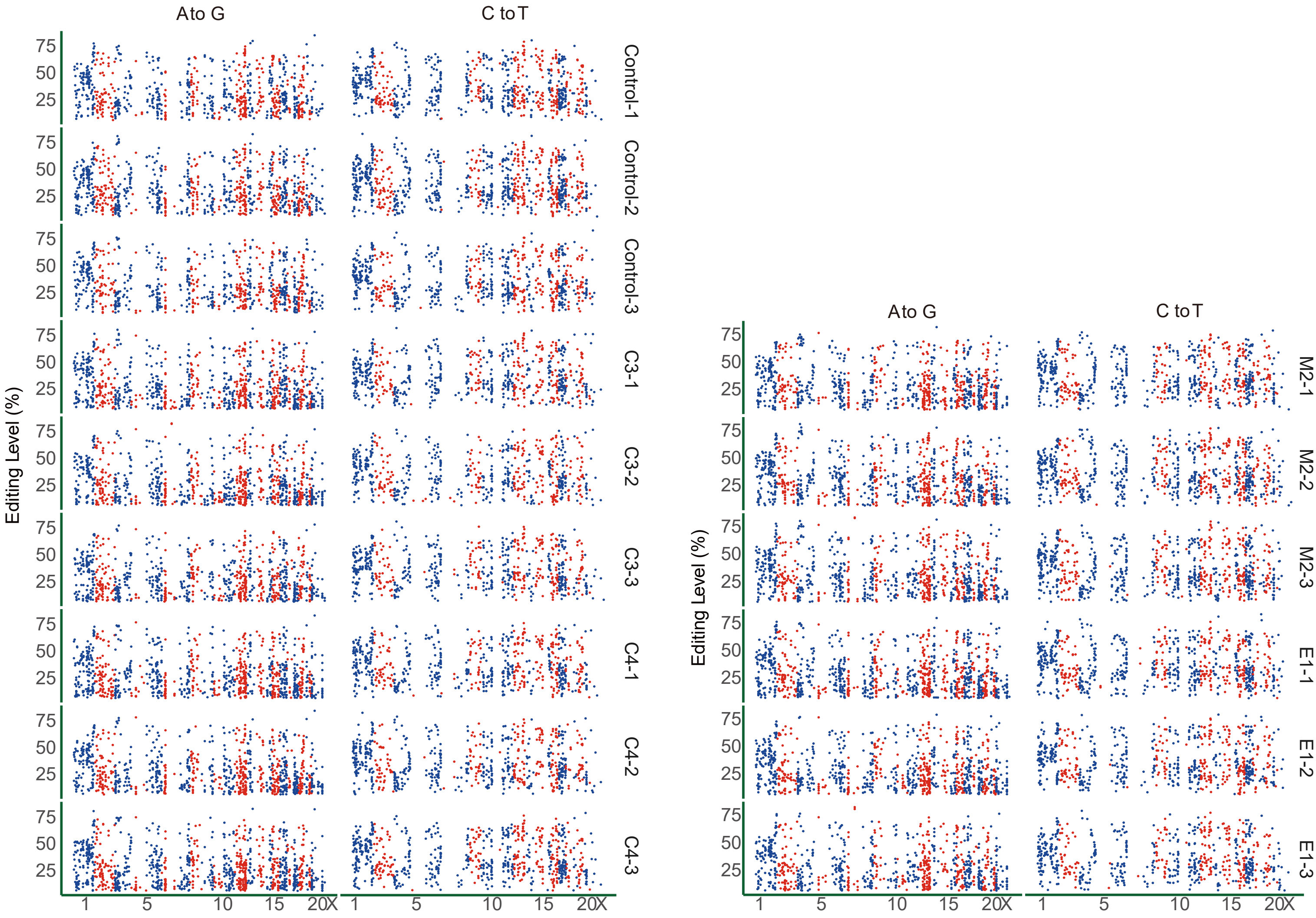

A to G
C to T
Control-1
Control-2
Control-3
C3-1
C3-2
C3-3
C4-1
C4-2
C4-3
M2-1
M2-2
M2-3
E1-1
E1-2
E1-3
Editing Level (%)
75
50
25
1
5
10
15
20X

**Figure S5. Manhattan plots of transcriptome-wide off-target RNA editing analysis for different mxABE constructs.**

Non-targeting mxABE construct (control) and E1, C3, C4, and M2 constructs were transfected into HEK293T cells. mCherry-positive cells were sorted and endogenous transcripts were analyzed by deep sequencing. The x and y axes are proportionally enlarged with each Manhattan plot to make the axis legend clear (n=3).

Figure S6

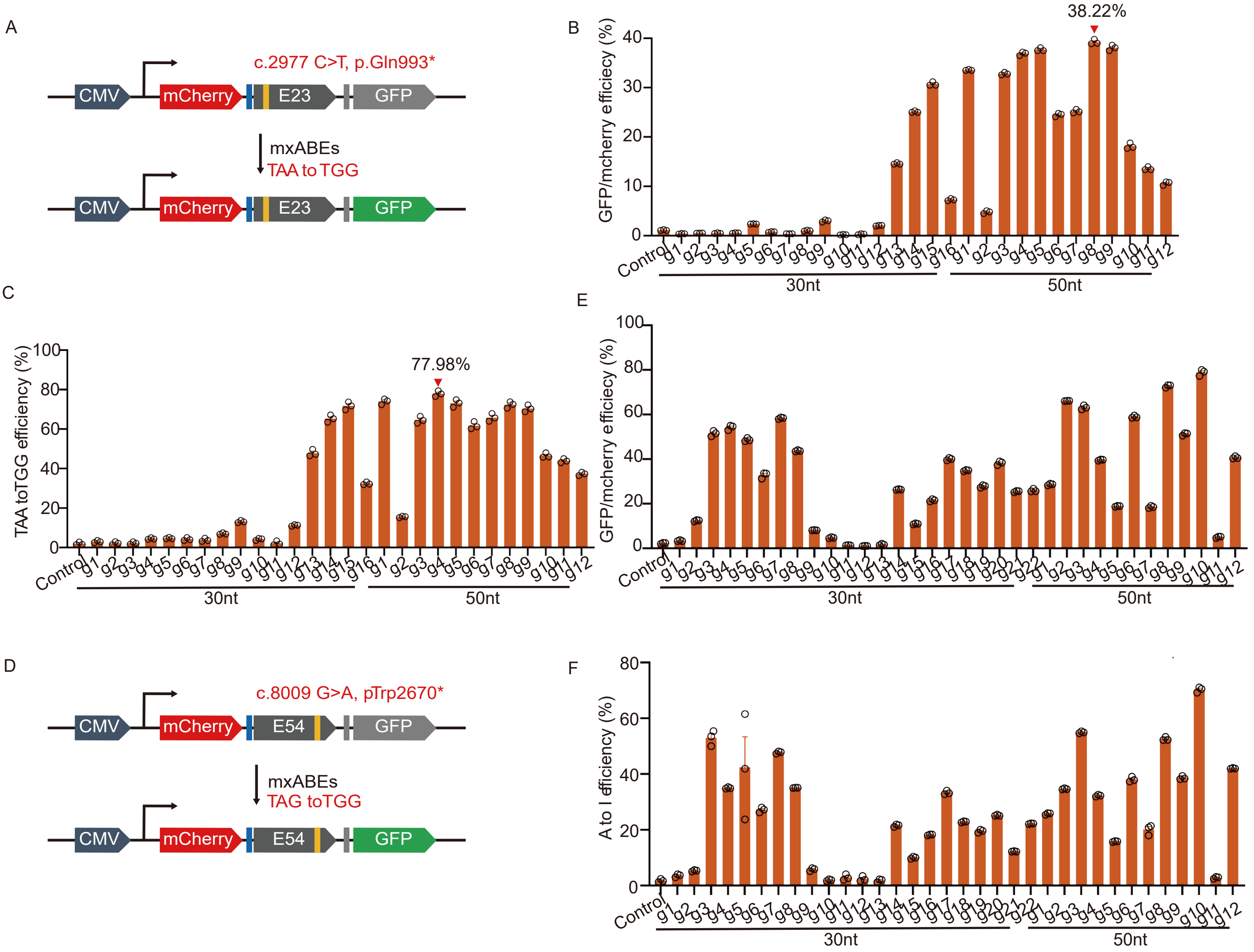

A
c.2977 C>T, p.Gln993*
CMV
mCherry
E23
GFP
mxABEs
TAA to TGG
B
GFP/mcherry efficiecy (%)
38.22%
30nt
50nt
C
TAA toTGG efficiency (%)
77.98%
30nt
50nt
D
c.8009 G>A, pTrp2670*
CMV
mCherry
E54
GFP
mxABEs
TAG toTGG
E
GFP/mchery efficiecy (%)
30nt
50nt
F
A to I efficiency (%)
30nt
50nt

**Figure S6. mxABE-mediated A to I correction of other mutations in the *DMD* gene.**

Schematic diagram of reporter cassettes containing an mCherry sequence fused with 2A peptide, mutant human *DMD* exon 23 (A) or exon 54 (D) and ATG-removed GFP. Correction of the stop codon within the target sequence would allow EGFP expression. Flow cytometry analysis of GFP expression in HEK293T cells transfected with 28 gRNAs targeting exon 23 (B) and exon 54 (E). Deep sequencing of the reporter RNA after GFP rescue experiment in exon 23 (C) and exon 54 (F). Data are represented as mean ± SEM (n=3).

Figure S7

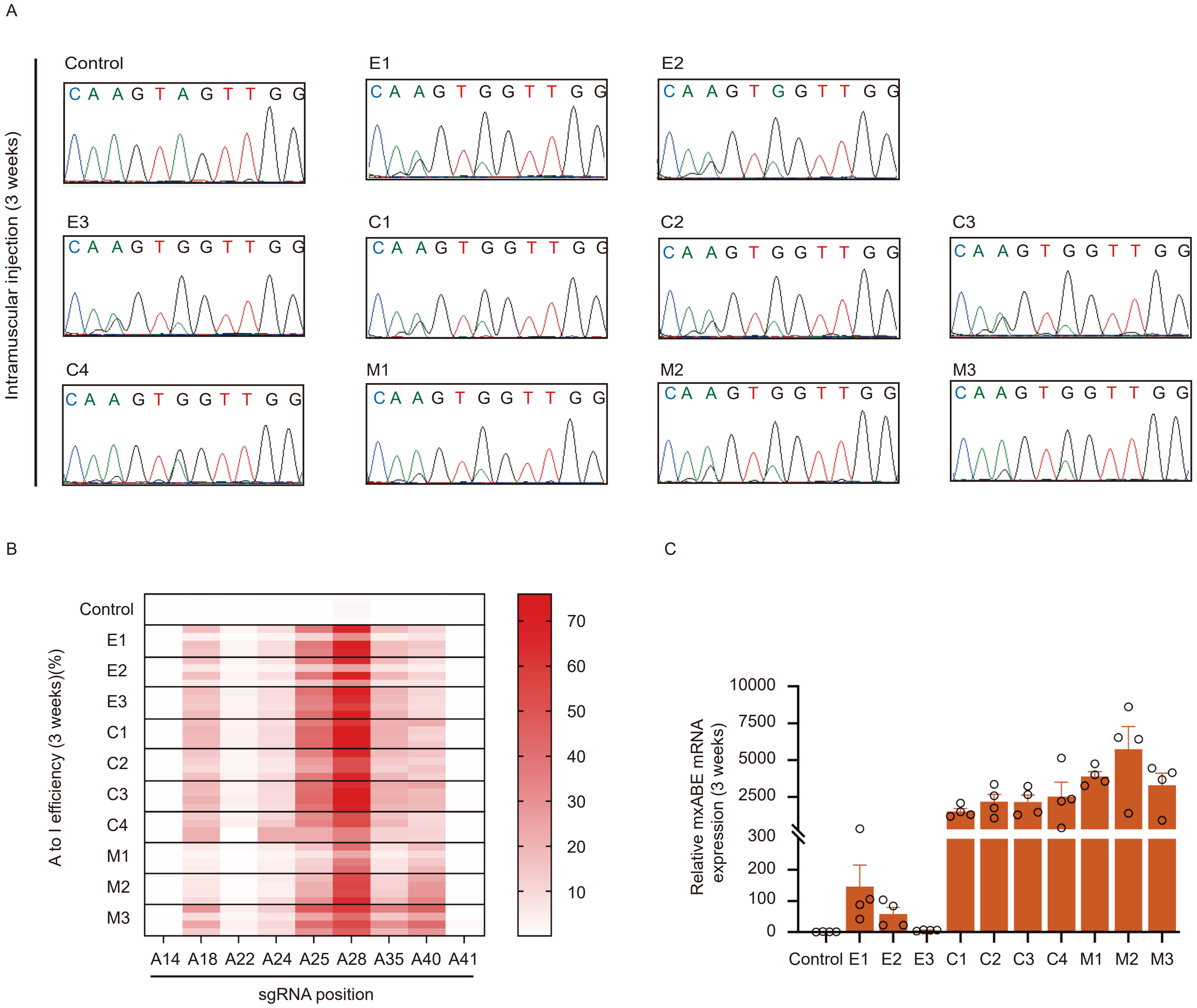
A
Intramuscular injection (3 weeks)
Control
C A A G T A G T T G G
E1
C A A G T G G T T G G
E2
C A A G T G G T T G G
E3
C A A G T G G T T G G
C1
C A A G T G G T T G G
C2
C A A G T G G T T G G
C3
C A A G T G G T T G G
C4
C A A G T G G T T G G
M1
C A A G T G G T T G G
M2
C A A G T G G T T G G
M3
C A A G T G G T T G G
B
A to I efficiency (3 weeks)(%)
Control
E1
E2
E3
C1
C2
C3
C4
M1
M2
M3
A14 A18 A22 A24 A25 A28 A35 A40 A41
sgRNA position
70
60
50
40
30
20
10
C
Relative mxABE mRNA expression (3 weeks)
10000
7500
5000
2500
300
200
100
0
Control E1 E2 E3 C1 C2 C3 C4 M1 M2 M3

**Figure S7. *In vivo DMD* gene editing by IM injection with mxABEs at 3 weeks.**

A. Representative Sanger sequencing trace of *DMD* transcripts 3 weeks after IM injection with ten AAV9-mxABE particles. B. Heat map indicates the A>G edits in the vicinity of the target. C. qPCR analysis of mxABE expression in TA muscles 3 weeks after intramuscular injection. Values are shown as mean ± SEM (n=4).

Figure S8

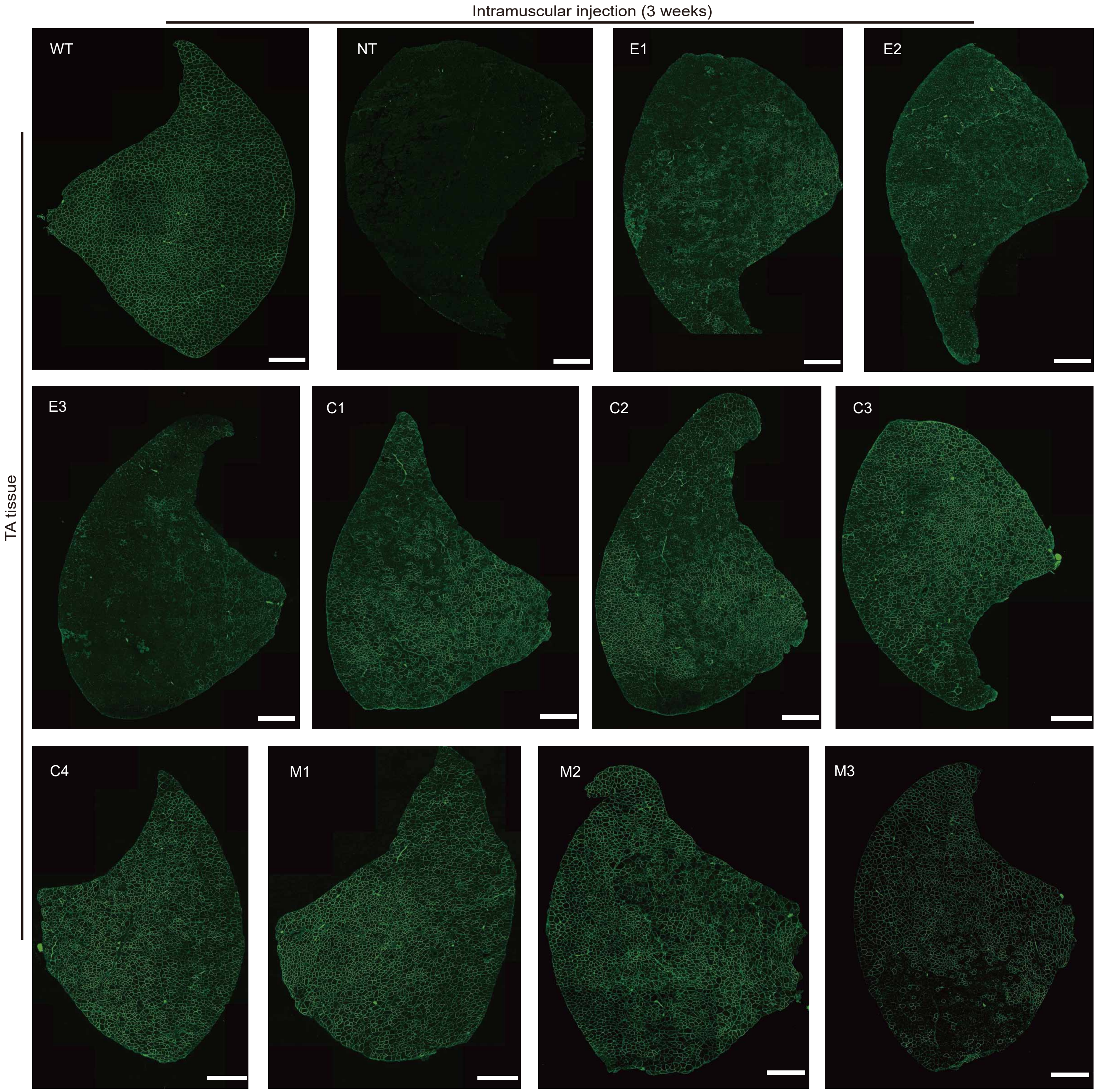
Intramuscular injection (3 weeks)
TA tissue
WT
NT
E1
E2
E3
C1
C2
C3
C4
M1
M2
M3

**Figure S8. Rescue of dystrophin expression following IM injection of mxABEs in DMD$^{E30mut}$ mice.**

Dystrophin immunohistochemistry of entire TA muscle. Control mice were injected with saline. Dystrophin (Abcam, ab15277) is shown in green. Scale bar, 500 µm.

Figure S9

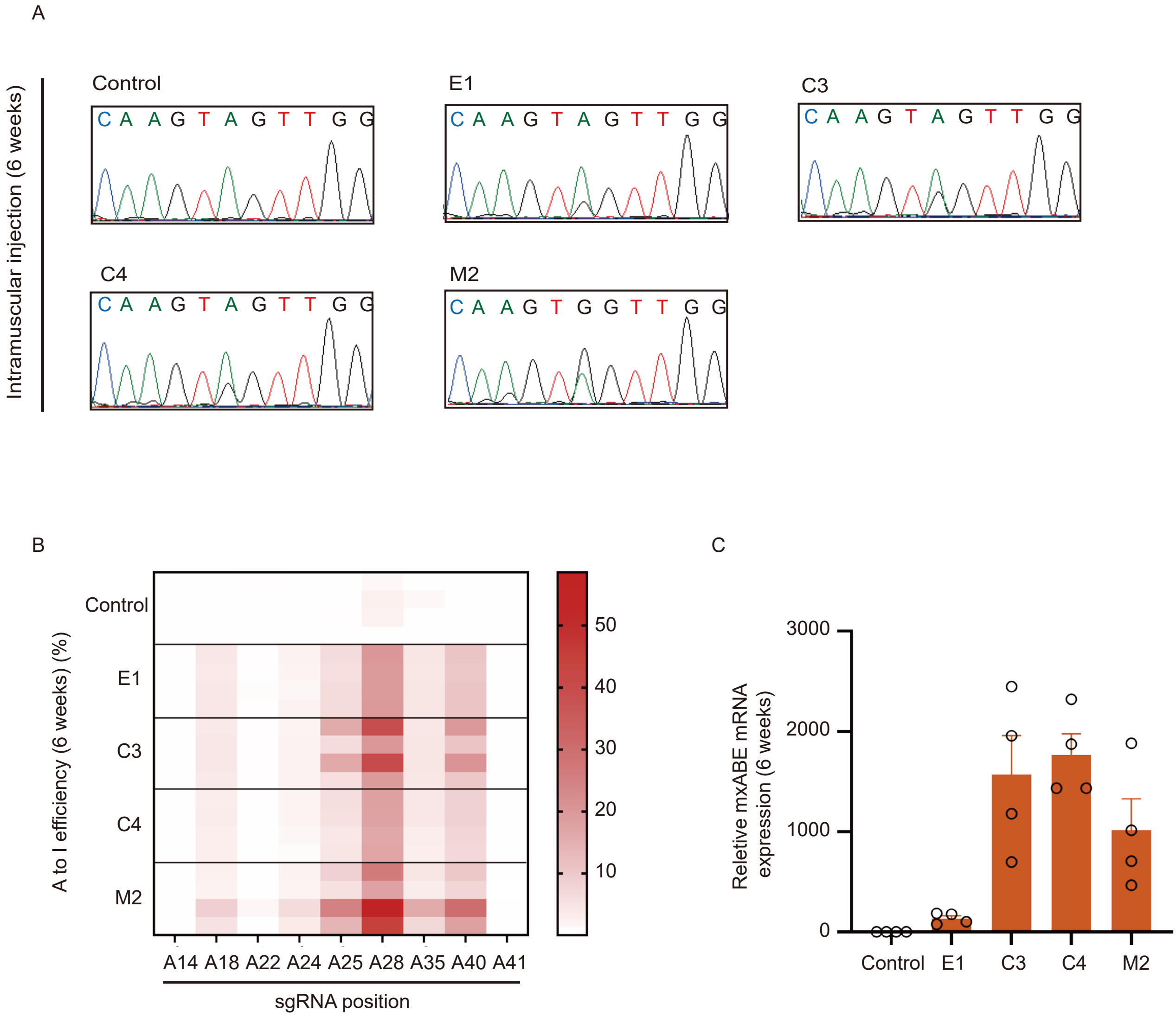
A
Intramuscular injection (6 weeks)
Control
C A A G T A G T T G G
E1
C A A G T A G T T G G
C3
C A A G T A G T T G G
C4
C A A G T A G T T G G
M2
C A A G T G G T T G G
B
A to I efficiency (6 weeks) (%)
Control
E1
C3
C4
M2
A14 A18 A22 A24 A25 A28 A35 A40 A41
sgRNA position
50
40
30
20
10
C
Reletive mxABE mRNA
expression (6 weeks)
3000
2000
1000
0
Control
E1
C3
C4
M2

**Figure S9. In vivo *DMD* gene editing by IM injection with mxABEs after 6 weeks.**

A. Representative Sanger sequencing trace of *DMD* transcripts 6 weeks after IM injection with four AAV9-mxABE particles. B. Heat map indicates the A>G edits in the vicinity of the target. C. qPCR analysis of mxABE expression in TA muscles 6 weeks after intramuscular injection. Values are shown as mean ± SEM (n=4).

Figure S10

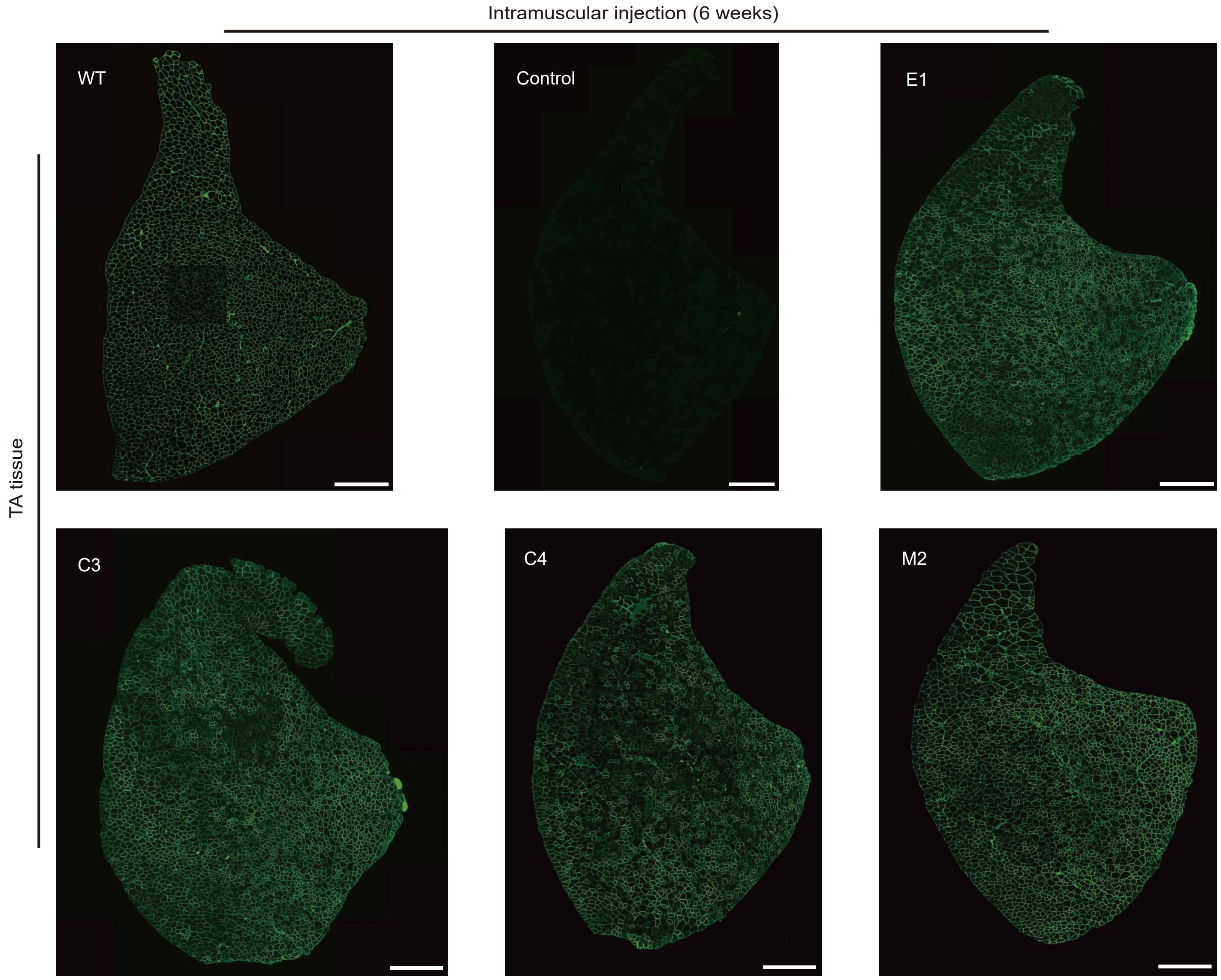

Intramuscular injection (6 weeks)
TA tissue
WT
Control
E1
C3
C4
M2

**Figure S10. Intramuscular AAV9 delivery of gene editing components rescues dystrophin expression.**

Immunohistochemistry of dystrophin in entire TA muscle 6 weeks after intramuscular AAV9 delivery. Control mice were injected with saline. Dystrophin (Abcam, ab15277) is showed in green. Scale bar, 500 µm.

Figure S11

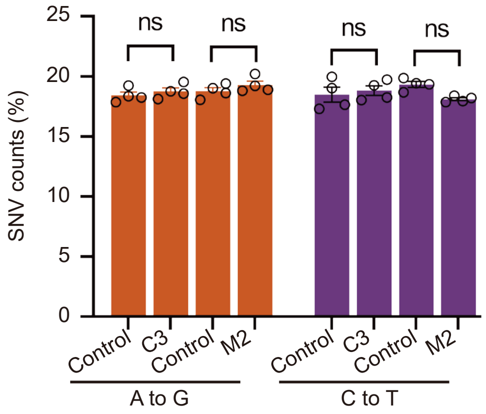

SNV counts (%)
25
20
15
10
5
0
ns
ns
ns
ns
Control
C3
Control
M2
Control
C3
Control
M2
A to G
C to T

**Figure S11. Calculation of SNV counts by transcriptome-wide off-target analysis *in vivo*.**

The transcriptome-wide off-target effect of two AAV9-mxABEs (C3 and M2) were analyzed *in vivo*. Control mice were injected with saline. Values are shown as mean ± SEM (n=4). NS, not statistically significant using unpaired two-tailed Student's t tests (P<0.01).

Figure S12

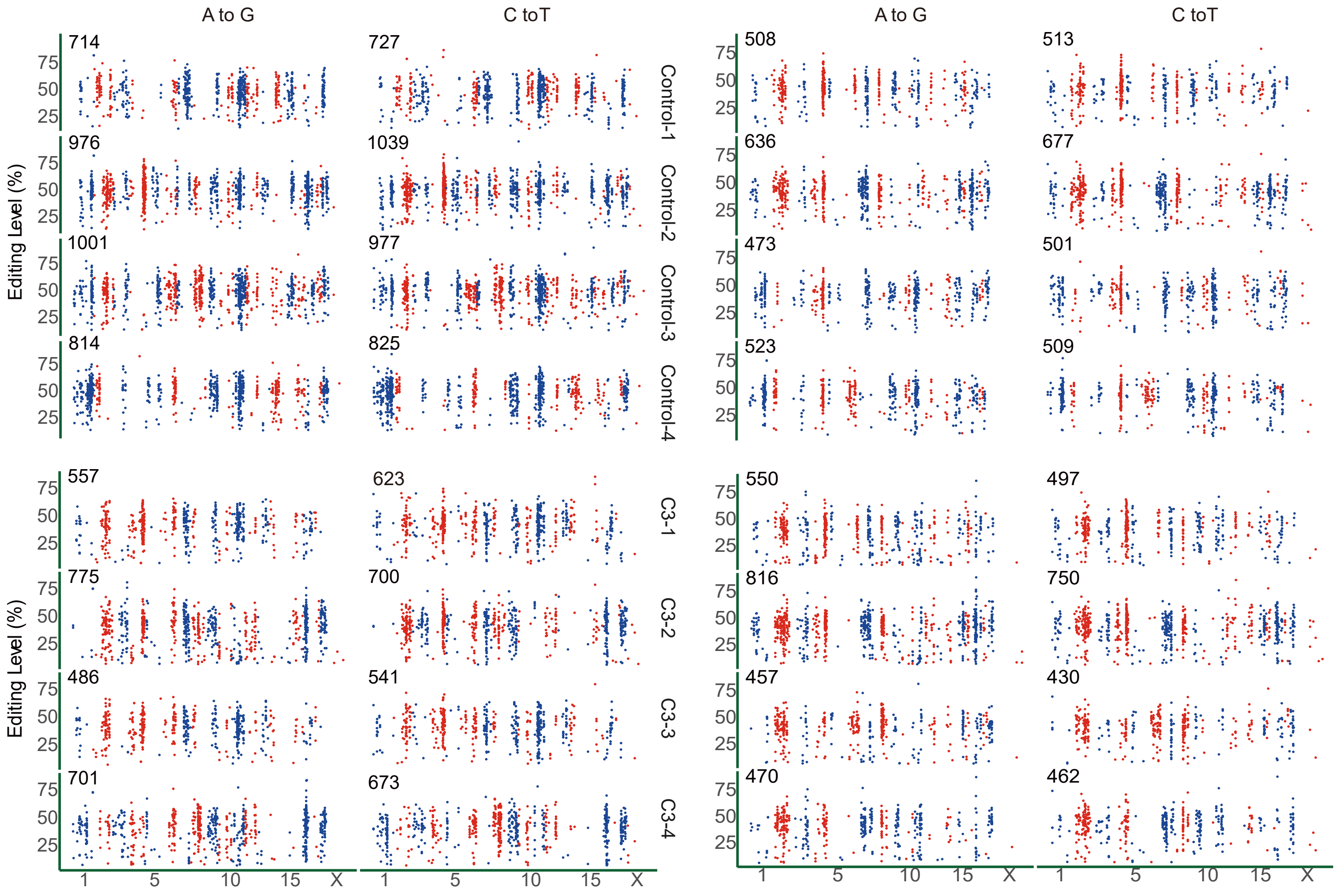

A to G
C toT
A to G
C toT
Editing Level (%)
Editing Level (%)
714
727
508
513
976
1039
636
677
1001
977
473
501
814
825
523
509
557
623
550
497
775
700
816
750
486
541
457
430
701
673
470
462
75
50
25
Control-1
Control-2
Control-3
Control-4
C3-1
C3-2
C3-3
C3-4
M2-1
M2-2
M2-3
M2-4
1
5
10
15
X

**Figure S12. Manhattan plots of transcriptome-wide off-target RNA editing analysis *in vivo*.**

Control mice were injected with saline. The x and y axes are proportionally enlarged with each Manhattan plot to make the axis legend clear. n=4.

Figure S13

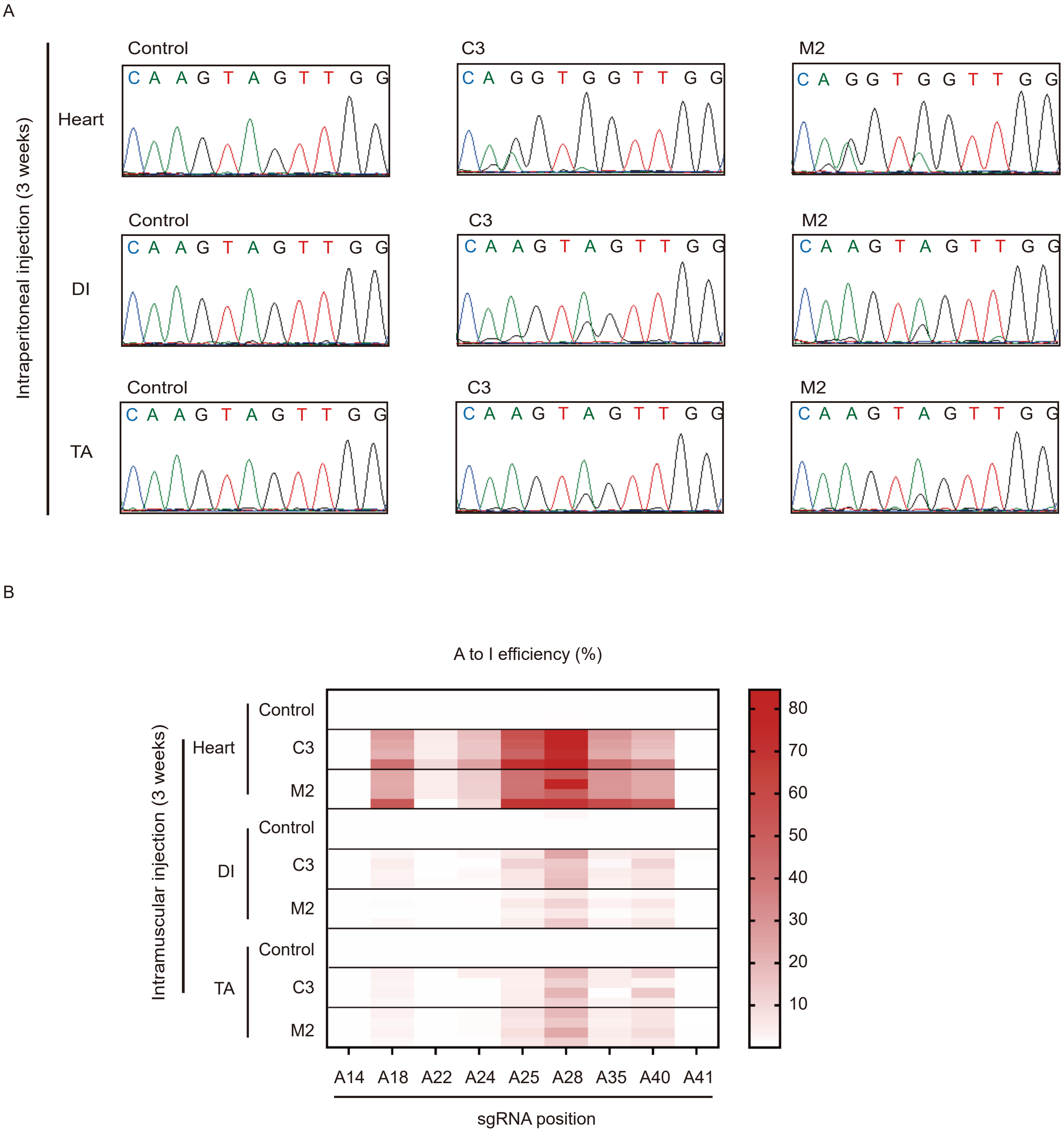
A
Intraperitoneal injection (3 weeks)
Heart
DI
TA
Control
C3
M2
C A A G T A G T T G G
C A G G T G G T T G G
C A A G T A G T T G G
B
A to I efficiency (%)
Intramuscular injection (3 weeks)
Heart
DI
TA
Control
C3
M2
80
70
60
50
40
30
20
10
A14 A18 A22 A24 A25 A28 A35 A40 A41
sgRNA position

**Figure S13. Gene editing 3 weeks after systemic delivery of mxABEs in DMD$^{E30mut}$ mice.**

A. Representative Sanger sequencing trace of *DMD* transcripts in TA, DI, and cardiac muscle 3 weeks after intraperitoneal (IP) injection with C3 and M2 particles. B. Heat map indicates the A>G edits in the vicinity of the target in indicated tissues. n=4.

Figure S14

A

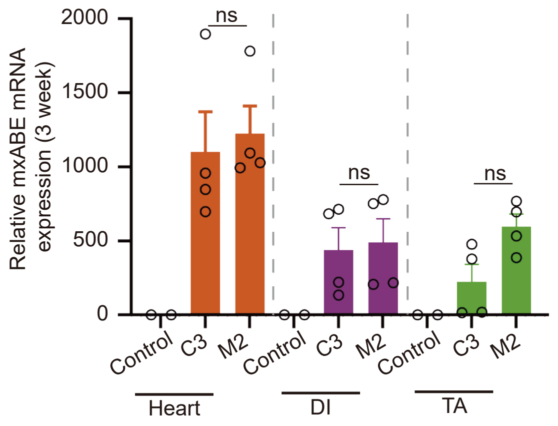


B

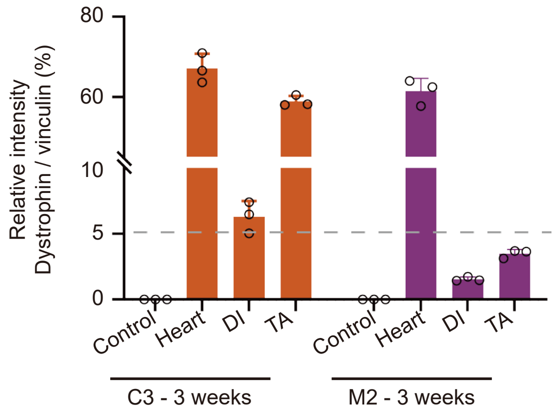

**Figure S14. AAV9-mxABE expression and dystrophin protein levels after systemic delivery in $DMD^{E30mut}$ mice.**

A. qPCR analysis of mxABE expression in TA, DI, and heart muscles 3 weeks after systemic delivery (n=4). B. Relative dystrophin (Sigma, D8168) intensity was calibrated against a vinculin (CST, 13901S) internal control before normalizing to the WT control. The results showed restoration of dystrophin in TA, DI, and heart muscles of $DMD^{E30mut}$ mice 3 weeks after systemic delivery of AAV9-packaged C3 and M2 (n=3). Values are shown as mean ± SEM. NS, not statistically significant using unpaired two-tailed Student's t tests (P<0.01).

Figure S15

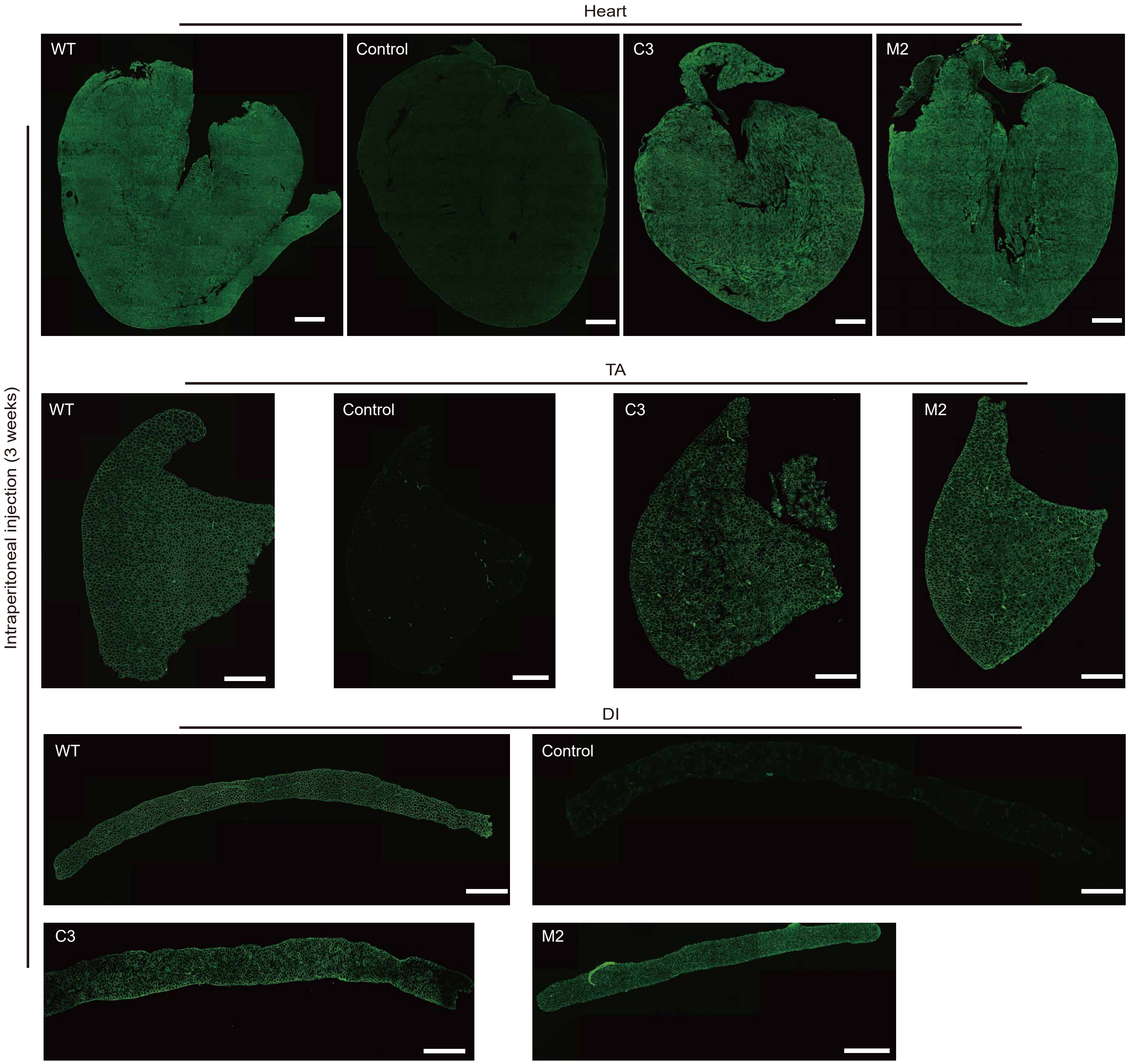
Heart
WT
Control
C3
M2
TA
WT
Control
C3
M2
DI
WT
Control
C3
M2
Intraperitoneal injection (3 weeks)

**Figure S15. Histological analysis of dystrophin expression 3 weeks after systemic delivery of AAV9-mxABE in DMD$^{E30mut}$ mice.**

Whole-muscle scanning of TA, DI, and heart of DMD$^{E30mut}$ mice 3 weeks after systemic delivery AAV9-C3 and M2 particles. Control mice were injected with saline. Dystrophin (Abcam, ab15277) is showed in green. Scale bar, 500 µm.

Figure S16

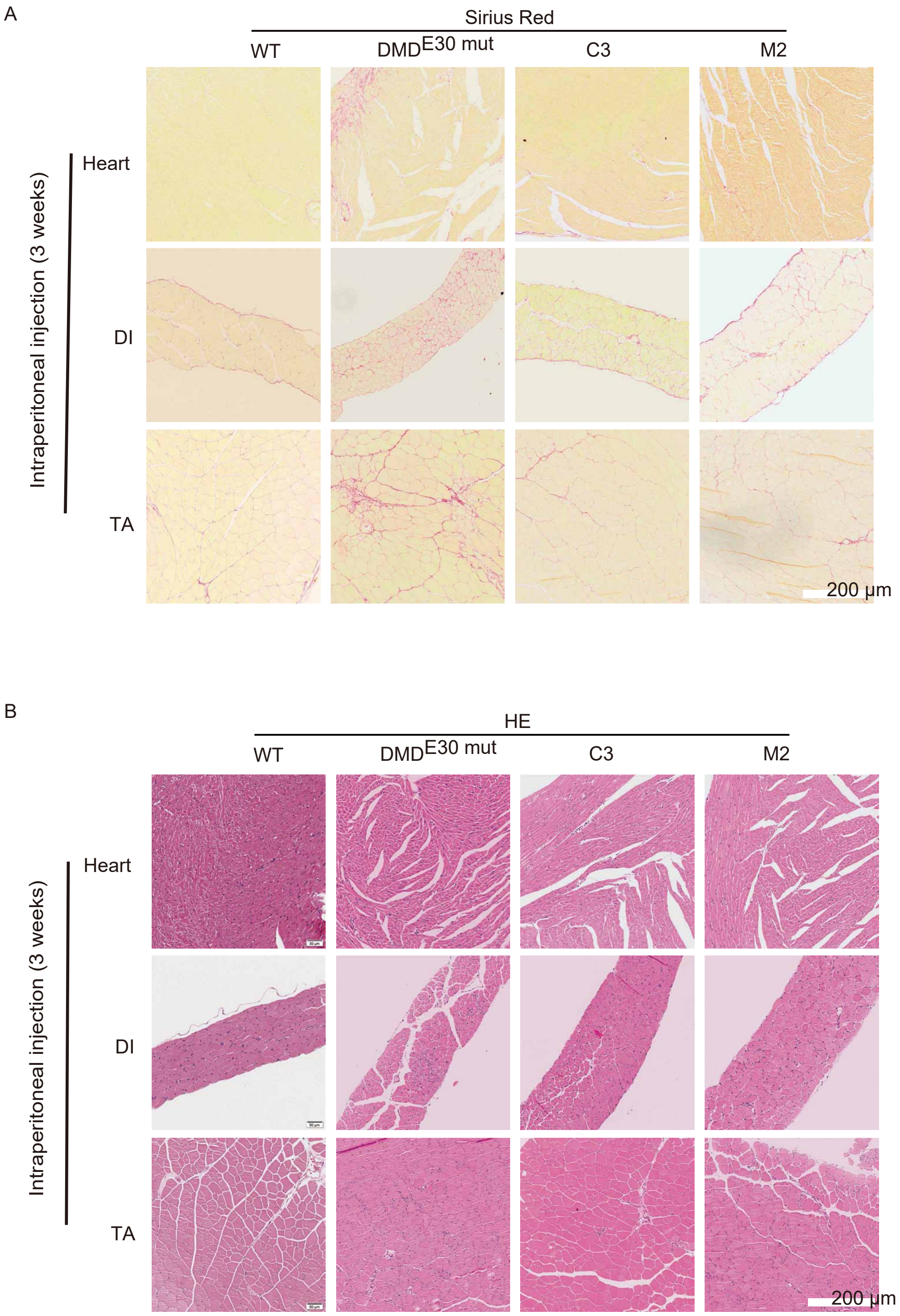
A
Sirius Red
WT
DMDE30 mut
C3
M2
Intraperitoneal injection (3 weeks)
Heart
DI
TA
200 μm
B
HE
WT
DMDE30 mut
C3
M2
Intraperitoneal injection (3 weeks)
Heart
DI
TA
200 μm

**Figure S16. Histological analysis 3 weeks after systemic delivery of AAV9-mxABE.**

Sirius red staining (A) and HE staining (A) of TA, DI, and heart of WT, untreated $DMD^{E30mut}$ mice and $DMD^{E30mut}$ mice treated with AAV9-mxABE 3 weeks after systemic injection. n=4 for each group. Scale bars, 200 μm.

Figure S17

A

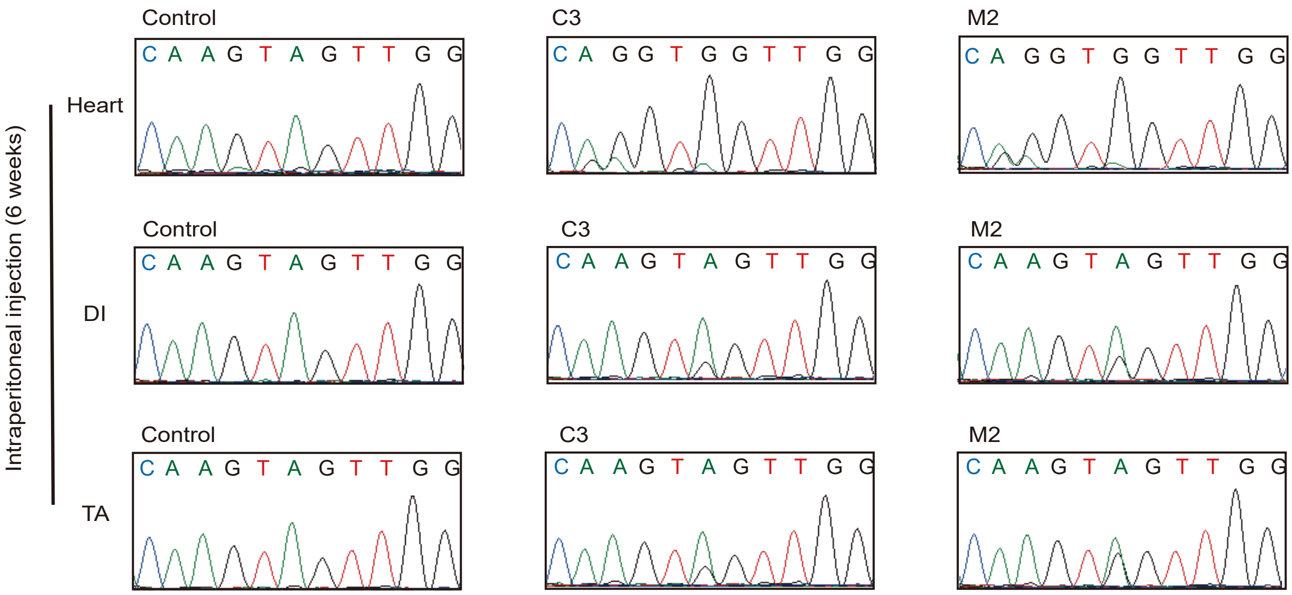


B

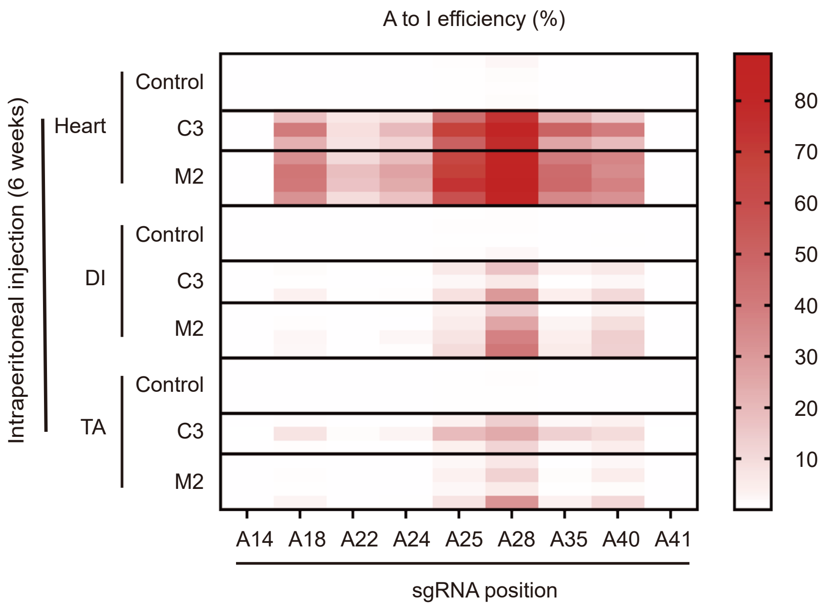

**Figure S17. Base editing efficiency achieved by systemic delivery of mxABE after 6 weeks.**

A. Representative Sanger sequencing trace of *DMD* transcripts in TA, DI, and heart tissues 6 weeks after intraperitoneal (IP) injection with C3 and M2 particles. B. Heat map shows base editing rates in tissues indicated after IP injection with C3 (n=3) and M2 (n=4).

Figure S18

A

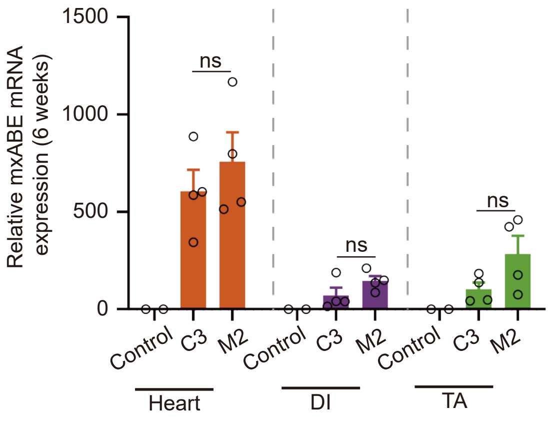


B

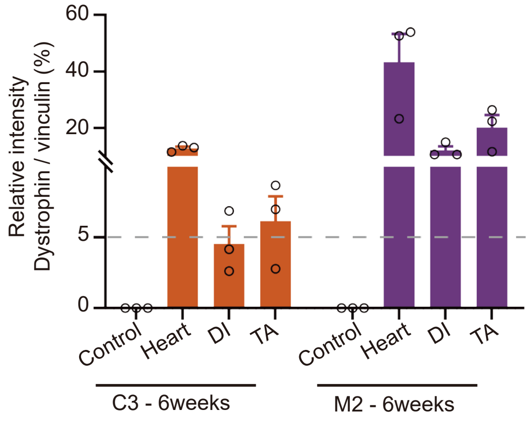


C

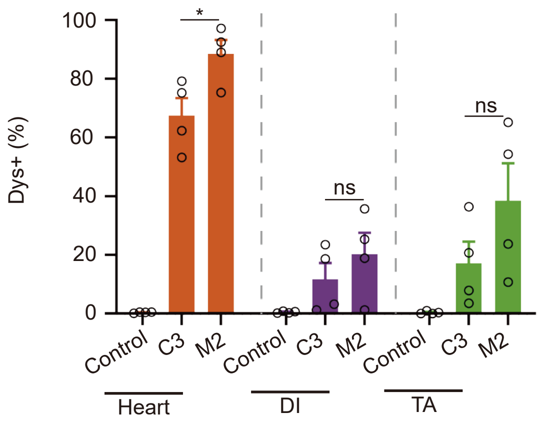

**Figure S18. mxABE expression and dystrophin restoration level after 6-week administration of AAV-mxABE systemically.**

A. qPCR analysis of mxABE expression in TA, DI, and heart muscles 6 weeks after systemic delivery (n=4). B. Relative dystrophin (Sigma, D8168) intensity was calibrated against a vinculin (CST, 13901S) internal control before normalizing to the WT control. The results showed restoration of dystrophin in TA, DI, and cardiac muscles of $DMD^{E30mut}$ mice 6 weeks after systemic delivery of C3 and M2 AAV-mxABE (n=3). C. Percentage of Dys+ tissue area in TA, DI, and heart from treated and untreated $DMD^{E30mut}$ mice (n=4). Values are shown as mean ± SEM. NS, not statistically significant using unpaired two-tailed Student’s t test (P<0.01).

Figure S19

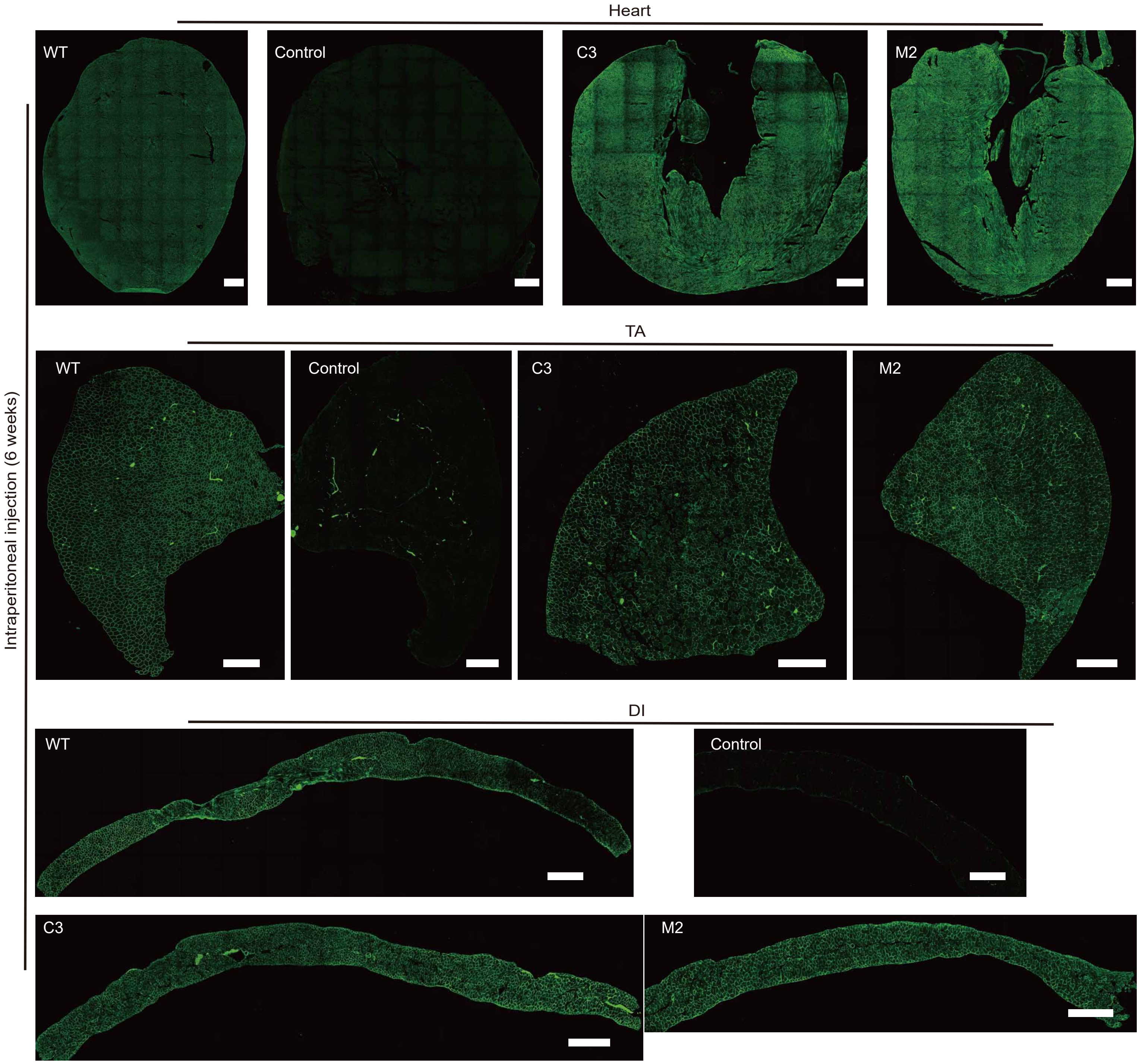
Heart
WT
Control
C3
M2
TA
WT
Control
C3
M2
DI
WT
Control
C3
M2
Intraperitoneal injection (6 weeks)

**Figure S19. Immunostaining of dystrophin in heart, TA, and DI tissues 6 weeks after systemic administration of AAV-mxABE.**

Whole-muscle scanning of TA, DI, and heart muscle of $DMD^{E30mut}$ mice 6 weeks after systemic delivery of AAV9-C3 and M2 particles. Control mice were injected with saline. Dystrophin (Abcam, ab15277) is shown in green. Scale bar, 500 µm. Images shown in both Figure 6C and Figure S19 were obtained from the same tissue at 20x magnification. Figure 6C showed the local region staining image rather than the reconstituted whole-tissue scanning image in Figure S19.

Figure S20

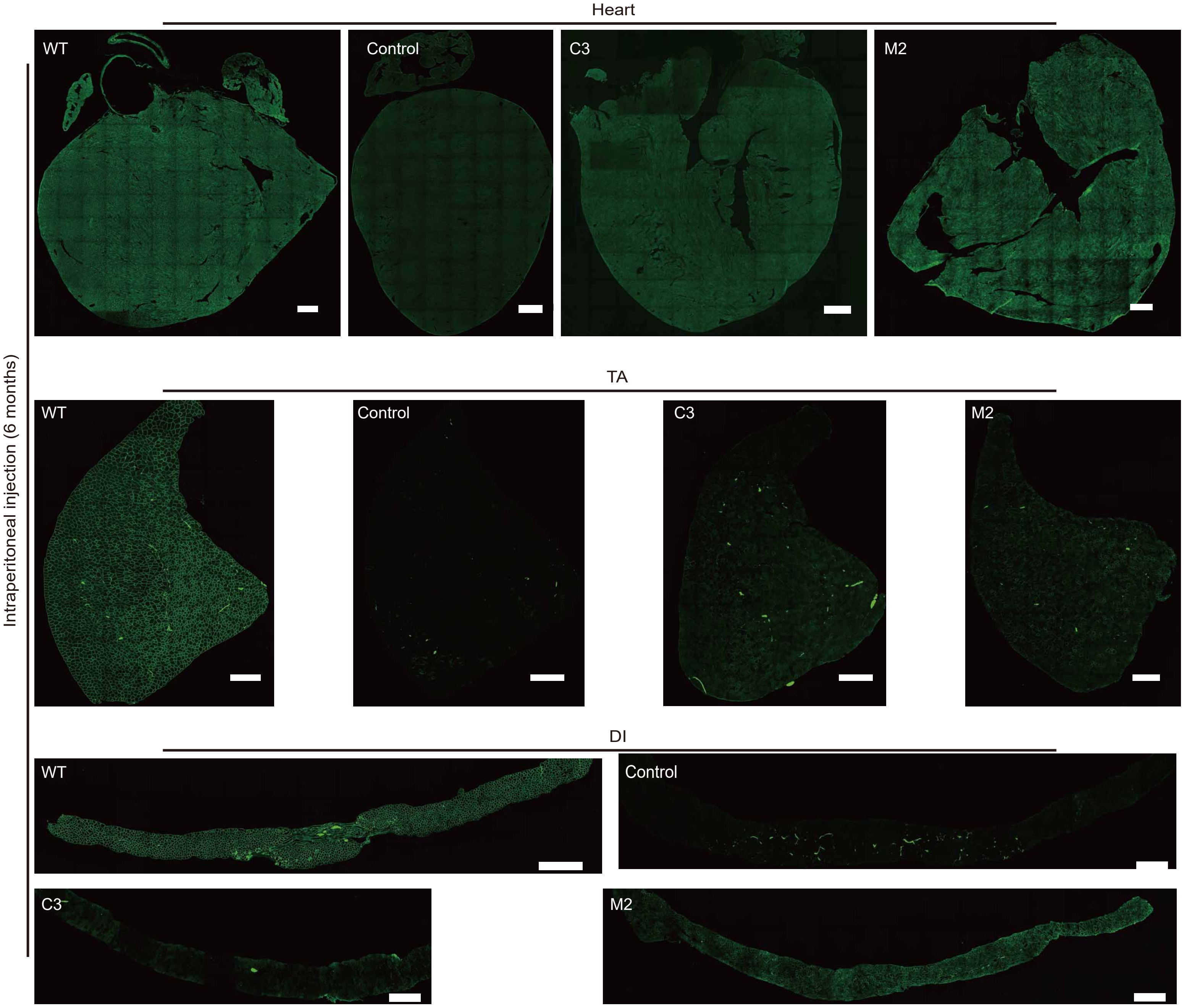
Heart
WT
Control
C3
M2
TA
WT
Control
C3
M2
DI
WT
Control
C3
M2
Intraperitoneal injection (6 months)

**Figure S20. Immunostaining of dystrophin in heart, TA, and DI 6 months after systemic administration of AAV-mxABE.**

Whole-muscle scanning of TA, DI, and heart muscle of $DMD^{E30mut}$ mice 6 months after systemic delivery of AAV9-C3 and M2 particles. Control mice were injected with saline. Dystrophin (Abcam, ab15277) is shown in green. Scale bar, 500 µm.

Figure S21

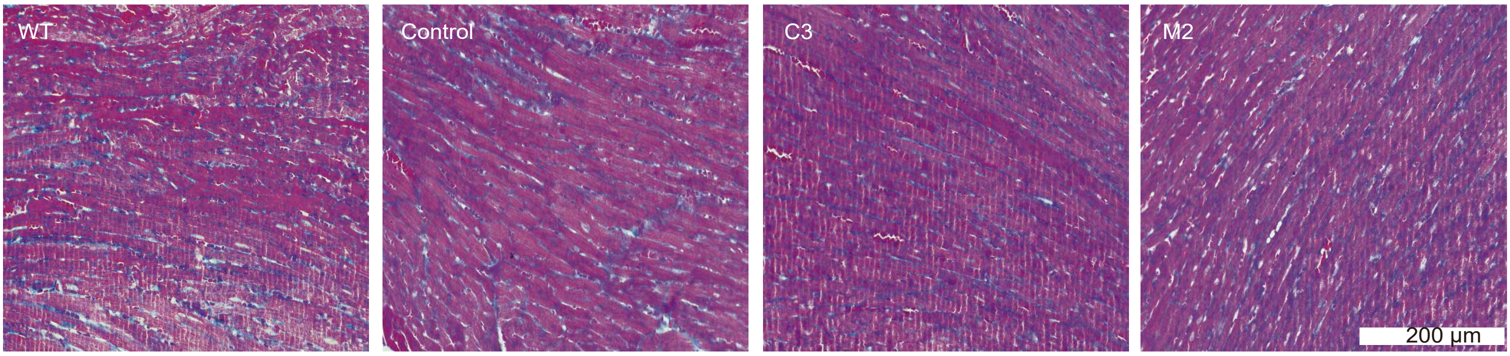

**Figure S21. Collagen staining shows no obvious fibrosis in treated and untreated hearts 6 months after systemic administration of AAV-mxABE.** Scale bar, 200 µm.

Figure S22

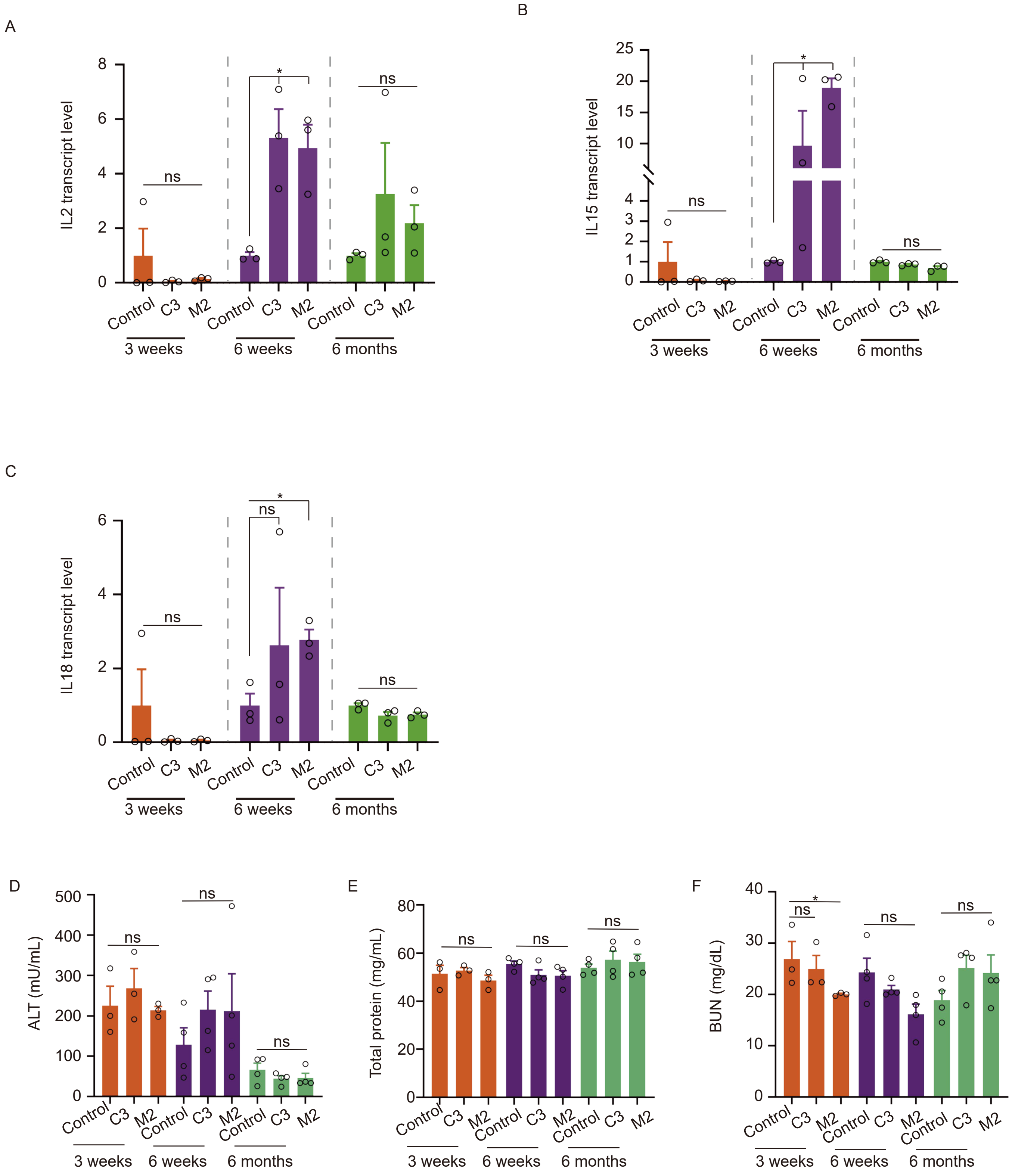
A
8
6
4
2
0
IL2 transcript level
ns
*
ns
Control
C3
M2
3 weeks
6 weeks
6 months
B
25
20
15
10
4
3
2
1
0
IL15 transcript level
C
IL18 transcript level
D
500
400
300
200
100
ALT (mU/mL)
E
80
60
40
20
Total protein (mg/mL)
F
40
30
20
10
BUN (mg/dL)

**Figure S22. Host immune response to AAV-mxABE treatment indicates no overt toxicity after 6 months.**

(A-C) IL2, IL15, and IL18 transcript levels at baseline for untreated mice, 3 weeks, 6 weeks, and 6 months for treated mice (n=3). (D-F) No obvious change in ALT, total protein and BUN level after AAV-mxABE treatment (n=3). Significance is indicated by asterisk ($P < 0.05$). NS represents not statistically significant using unpaired two-tailed Student’s t test.

Figure S23

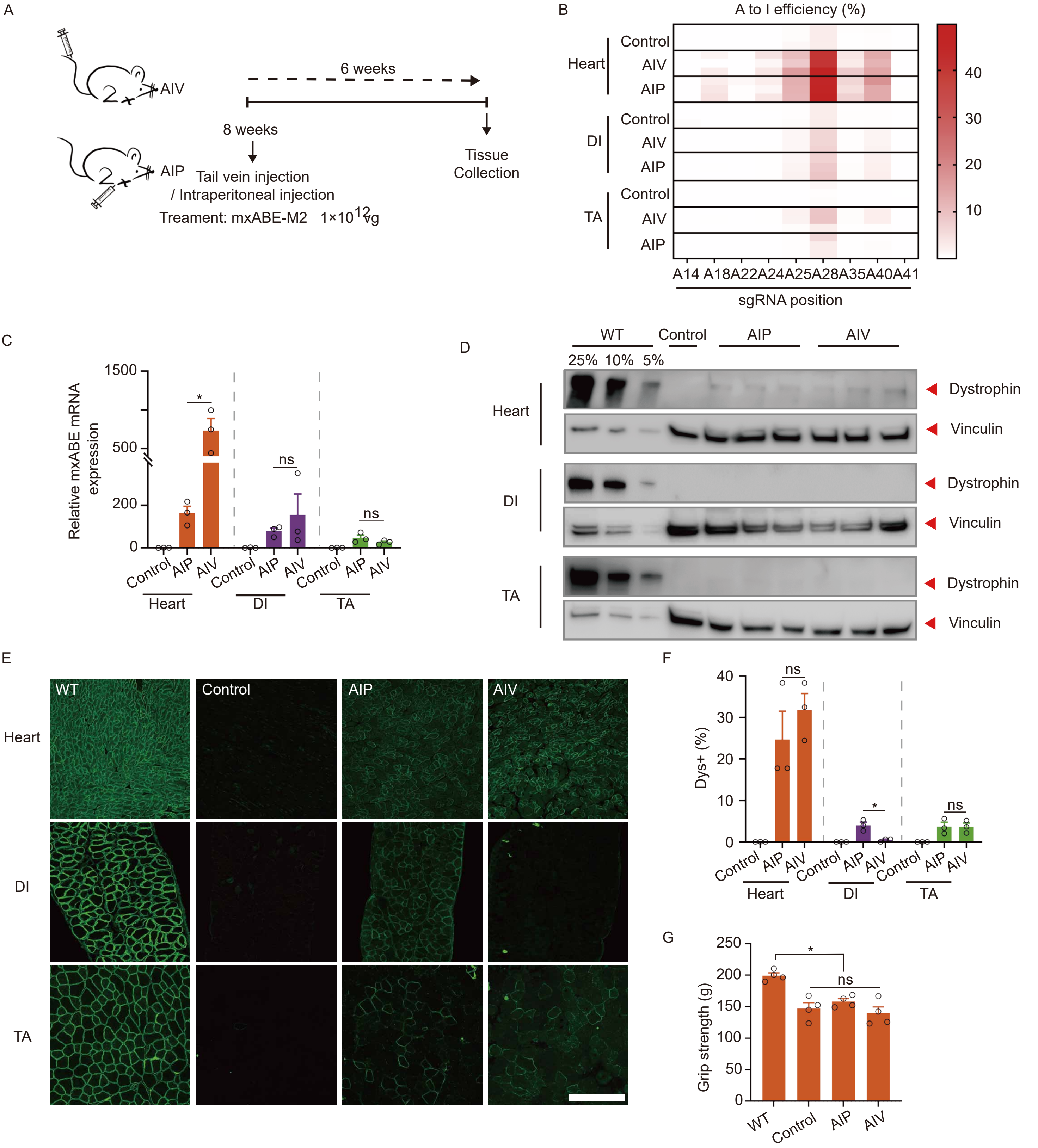

A
AIV
AIP
6 weeks
8 weeks
Tail vein injection
/ Intraperitoneal injection
Treament: mxABE-M2 1×10^12 vg
Tissue Collection
B
A to I efficiency (%)
Heart
DI
TA
Control
AIV
AIP
A14 A18 A22 A24 A25 A28 A35 A40 A41
sgRNA position
40
30
20
10
C
Relative mxABE mRNA expression
1500
500
200
0
Control AIP AIV
Heart
DI
TA
*
ns
D
WT
25% 10% 5%
Control
AIP
AIV
Dystrophin
Vinculin
E
WT
Control
AIP
AIV
Heart
DI
TA
F
Dys+ (%)
40
30
20
10
0
ns
*
G
Grip strength (g)
250
200
150
100
50
0
WT Control AIP AIV

**Figure S23. Systemic delivery of AAV-mxABE rescued dystrophin deficiency in adult DMD$^{E30mut}$ mice.**

A. Experimental design for mxABE administration and therapeutic analysis in adult DMD$^{E30mut}$ mice. Both intravenous (AIV group) and intraperitoneal (AIP group) injection of AAV-mxABE were evaluated. B. Heatmap shows base editing rate in heart, DI, and TA muscles (n=3). C. mxABE expression level in heart, DI, and TA muscles. D. Dystrophin restoration level analyzed by western blot in heart, DI, and TA muscles of treated and untreated mice. E. Tissue section immunostaining for dystrophin in heart, DI, and TA muscles of treated and untreated mice. Scale bar, 200 µm. F. Quantification of Dys+ immunostaining in (E) (n=3). G. Forelimb grip strength was measured in WT, DMD$^{E30mut}$ mice, and DMD$^{E30mut}$ mice treated with AAV9-M2 (n=4). Significance is indicated by asterisk ($P < 0.05$). NS represents not statistically significant using unpaired two-tailed Student's t test.

Figure S24

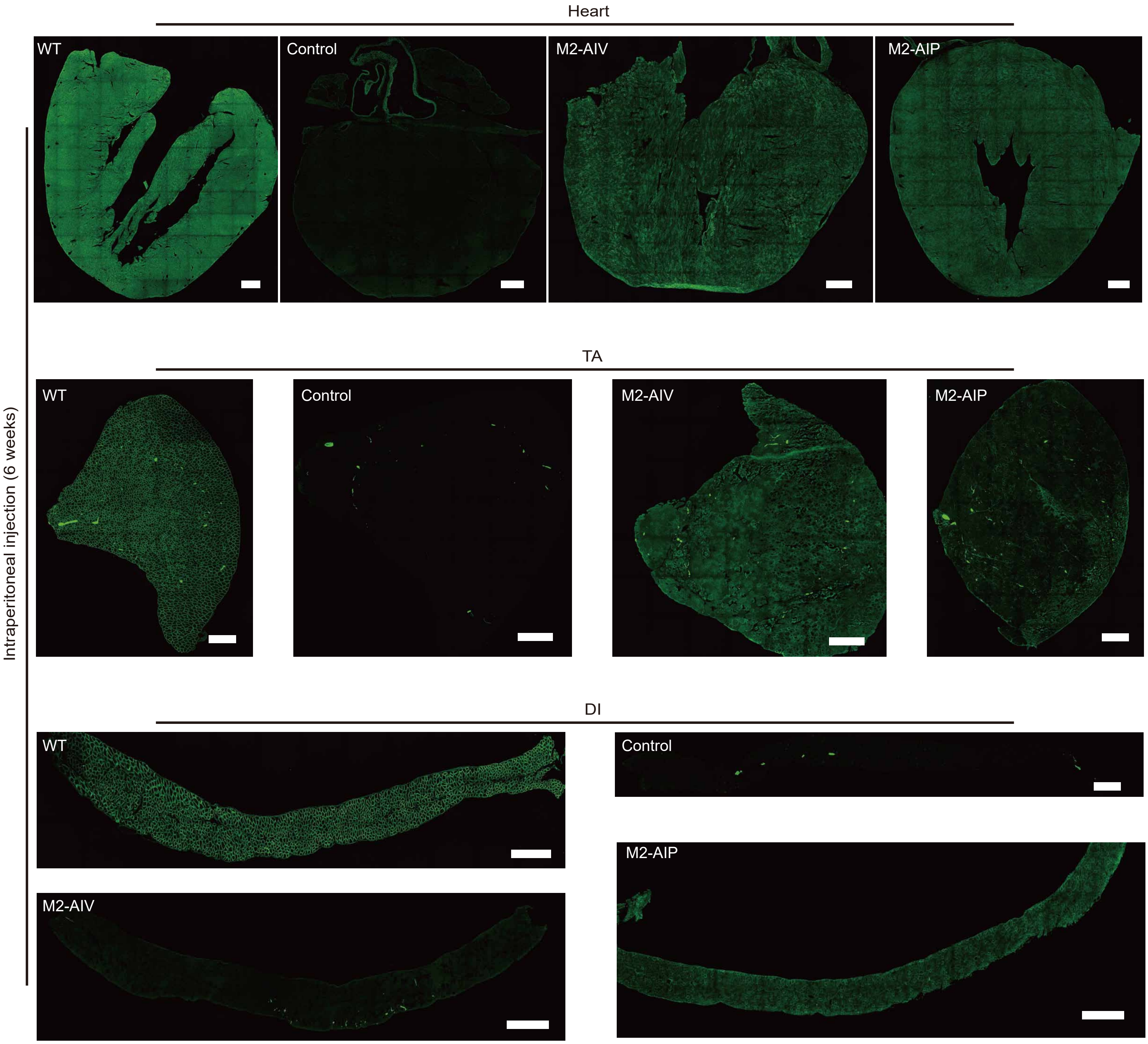
Heart
WT
Control
M2-AIV
M2-AIP
TA
WT
Control
M2-AIV
M2-AIP
DI
WT
Control
M2-AIV
M2-AIP
Intraperitoneal injection (6 weeks)

**Figure S24. Immunostaining of dystrophin in adult DMD$^{E30mut}$ mice 6 weeks after systemic injection of AAV-mxABE.**
Scale bar, 500 µm.

## Supplementary Table S1: Target sgRNA and primer sequence.

| Experiment | Primer name | Primer sequence (5'-3') |
|---|---|---|
| sgRNA for generation $DMD^{E30mut}$ model | DMD-T7sgRNA1 | CTTCTTCTATTTAGGCTGTG |
| | DMD-T7sgRNA2 | AGCTTATATCACTGACAAGG |
| Genotyping of $DMD^{E30mut}$ mice | $DMD^{E30mut}$-2333F | ATTCATATAGGGCTTCAGTTCC |
| | $DMD^{E30mut}$-2333R | CATCTGTTTTAATAGTGTGCAT |
| RT-PCR primer flanking exon 30 | RT-DMD-358F | AATCAGATTCGTCTATTGGCACA |
| | RT-DMD-358R | CCCTTTGGTTGGCATCCTT |
| RNA base editing sgRNA for exon 30 | 30nt-g1 | GCTGCCAACCACTTGTCAATGAATGTGAGG |
| | 30nt-g2 | AGCTGCCAACCACTTGTCAATGAATGTGAG |
| | 30nt-g3 | TGCAATATAAGCTGCCAACCACTTGTCAAT |
| | 30nt-g4 | CTGCAATATAAGCTGCCAACCACTTGTCAA |
| | 30nt-g5 | AAGCTGCCAACCACTTGTCAATGAATGTGA |
| | 30nt-g6 | TAAGCTGCCAACCACTTGTCAATGAATGTG |
| | 30nt-g7 | ATAAGCTGCCAACCACTTGTCAATGAATGT |
| | 30nt-g8 | TATAAGCTGCCAACCACTTGTCAATGAATG |
| | 30nt-g9 | ATATAAGCTGCCAACCACTTGTCAATGAAT |
| | 30nt-g10 | AATATAAGCTGCCAACCACTTGTCAATGAA |
| | 30nt-g11 | CAATATAAGCTGCCAACCACTTGTCAATGA |
| | 30nt-g12 | GCAATATAAGCTGCCAACCACTTGTCAATG |
| | 50nt-g1 | TGCAATATAAGCTGCCAACCACTTGTCAATGAATGTGAGGGACTCCTGGA |
| | 50nt-g2 | CTGCAATATAAGCTGCCAACCACTTGTCAATGAATGTGAGGGACTCCTGG |
| | 50nt-g3 | CCACCTTGTCTGCAATATAAGCTGCCAACCACTTGTCAATGAATGTGAGG |
| | 50nt-g4 | TCCACCTTGTCTGCAATATAAGCTGCCAACCACTTGTCAATGAATGTGAG |
| | 50nt-g5 | TCTGCAATATAAGCTGCCAACCACTTGTCAATGAATGTGAGGGACTCCTG |
| | 50nt-g6 | GTCTGCAATATAAGCTGCCAACCACTTGTCAATGAATGTGAGGGACTCCT |
| | 50nt-g7 | TGTCTGCAATATAAGCTGCCAACCACTTGTCAATGAATGTGAGGGACTCC |

| | | |
|---|---|---|
| | 50nt-g8 | TTGTCTGCAATATAAGCTGCCAACCACTTGTCAATGAATGTGAGGGACTC |
| | 50nt-g9 | CTTGTCTGCAATATAAGCTGCCAACCACTTGTCAATGAATGTGAGGGACT |
| | 50nt-g10 | CCTTGTCTGCAATATAAGCTGCCAACCACTTGTCAATGAATGTGAGGGAC |
| | 50nt-g11 | ACCTTGTCTGCAATATAAGCTGCCAACCACTTGTCAATGAATGTGAGGGA |
| | 50nt-g12 | CACCTTGTCTGCAATATAAGCTGCCAACCACTTGTCAATGAATGTGAGGG |
| RT-PCR primer for Reporter | RT-rep765F | CCACAACGAGGACTACACCA |
| | RT-rep765R | TCCTTGAAGTCGATGCCCTT |
| qPCR primer | GAPDH-119F | TCAACGACCCCTTCATTGACC |
| | GAPDH-119R | CTTCCCGTTGATGACAAGCTTC |
| | Cas13X-140F | GCCATGCAGAAATAATATCTCGG |
| | Cas13X-140R | CATTCTCCTTCAGCCTAAACCC |
| RNA base editing sgRNA for exon 23 | 50nt-g1 | CTGAGATAGTATAGGCCACTccATTGCTCTTGCAGAGAACTTTGTAAAGC |
| | 50nt-g2 | GCTGAGATAGTATAGGCCACTCCATTGCTCTTGCAGAGAACTTTGTAAAG |
| | 50nt-g3 | TGCTGAGATAGTATAGGCCACTCCATTGCTCTTGCAGAGAACTTTGTAAA |
| | 50nt-g4 | GTGCTGAGATAGTATAGGCCACTCCATTGCTCTTGCAGAGAACTTTGTAA |
| | 50nt-g5 | GGTGCTGAGATAGTATAGGCCACTCCATTGCTCTTGCAGAGAACTTTGTA |
| | 50nt-g6 | TGGTGCTGAGATAGTATAGGCCACTCCATTGCTCTTGCAGAGAACTTTGT |
| | 50nt-g7 | GTGGTGCTGAGATAGTATAGGCCACTCCATTGCTCTTGCAGAGAACTTTG |
| | 50nt-g8 | CACAGTGGTGCTGAGATAGTATAGGCCACTCCATTGCTCTTGCAGAGAAC |
| | 50nt-g9 | TCACAGTGGTGCTGAGATAGTATAGGCCACTCCATTGCTCTTGCAGAGAA |
| | 50nt-g10 | AGTGGTGCTGAGATAGTATAGGCCACTCCATTGCTCTTGCAGAGAACTTT |
| | 50nt-g11 | CAGTGGTGCTGAGATAGTATAGGCCACTCCATTGCTCTTGCAGAGAACTT |
| | 50nt-g12 | ACAGTGGTGCTGAGATAGTATAGGCCACTCCATTGCTCTTGCAGAGAACT |
| | 30nt-g1 | TAGTATAGGCCACTCCATTGCTCTTGCAGA |
| | 30nt-g2 | ATAGTATAGGCCACTCCATTGCTCTTGCAG |
| | 30nt-g3 | GATAGTATAGGCCACTCCATTGCTCTTGCA |
| | 30nt-g4 | AGATAGTATAGGCCACTCCATTGCTCTTGC |
| | 30nt-g5 | GAGATAGTATAGGCCACTCCATTGCTCTTG |
| | 30nt-g6 | TGAGATAGTATAGGCCACTCCATTGCTCTT |

| | | |
|---|---|---|
| | 30nt-g7 | CTGAGATAGTATAGGCCACTCCATTGCTCT |
| | 30nt-g8 | GCTGAGATAGTATAGGCCACTCCATTGCTC |
| | 30nt-g9 | TGCTGAGATAGTATAGGCCACTCCATTGCT |
| | 30nt-g10 | GTGCTGAGATAGTATAGGCCACTCCATTGC |
| | 30nt-g11 | GGTGCTGAGATAGTATAGGCCACTCCATTG |
| | 30nt-g12 | AGTATAGGCCACTCCATTGCTCTTGCAGAG |
| | 30nt-g13 | TAGGCCACTCCATTGCTCTTGCAGAGAACT |
| | 30nt-g14 | ATAGGCCACTCCATTGCTCTTGCAGAGAAC |
| | 30nt-g15 | TATAGGCCACTCCATTGCTCTTGCAGAGAA |
| | 30nt-g16 | GTATAGGCCACTCCATTGCTCTTGCAGAGA |
| RNA base editing sgRNA for exon 54 | 50nt-g1 | TGTAATTCATACCTTTTATGAATGCTTCTCCAAGAGGCATTGATATTCTC |
| | 50nt-g2 | GTAATTCATACCTTTTATGAATGCTTCTCCAAGAGGCATTGATATTCTCT |
| | 50nt-g3 | CATACCTTTTATGAATGCTTCTCCAAGAGGCATTGATATTCTCTGTTATC |
| | 50nt-g4 | TCATACCTTTTATGAATGCTTCTCCAAGAGGCATTGATATTCTCTGTTAT |
| | 50nt-g5 | TTCATACCTTTTATGAATGCTTCTCCAAGAGGCATTGATATTCTCTGTTA |
| | 50nt-g6 | ATTCATACCTTTTATGAATGCTTCTCCAAGAGGCATTGATATTCTCTGTT |
| | 50nt-g7 | AATTCATACCTTTTATGAATGCTTCTCCAAGAGGCATTGATATTCTCTGT |
| | 50nt-g8 | TAATTCATACCTTTTATGAATGCTTCTCCAAGAGGCATTGATATTCTCTG |
| | 50nt-g9 | ATACCTTTTATGAATGCTTCTCCAAGAGGCATTGATATTCTCTGTTATCA |
| | 50nt-g10 | TACCTTTTATGAATGCTTCTCCAAGAGGCATTGATATTCTCTGTTATCAT |
| | 50nt-g11 | ACCTTTTATGAATGCTTCTCCAAGAGGCATTGATATTCTCTGTTATCATG |
| | 50nt-g12 | CCTTTTATGAATGCTTCTCCAAGAGGCATTGATATTCTCTGTTATCATGT |
| | 30nt-g1 | CATACCTTTTATGAATGCTTCTCCAAGAGG |
| | 30nt-g2 | TCATACCTTTTATGAATGCTTCTCCAAGAG |
| | 30nt-g3 | TTCATACCTTTTATGAATGCTTCTCCAAGA |
| | 30nt-g4 | ATACCTTTTATGAATGCTTCTCCAAGAGGC |
| | 30nt-g5 | TACCTTTTATGAATGCTTCTCCAAGAGGCA |
| | 30nt-g6 | ACCTTTTATGAATGCTTCTCCAAGAGGCAT |
| | 30nt-g7 | TCTCCAAGAGGCATTGATATTCTCTGTTAT |

| | | |
|---|---|---|
| | 30nt-g8 | TTCTCCAAGAGGCATTGATATTCTCTGTTA |
| | 30nt-g9 | TATGAATGCTTCTCCAAGAGGCATTGATAT |
| | 30nt-g10 | TTATGAATGCTTCTCCAAGAGGCATTGATA |
| | 30nt-g11 | TTTATGAATGCTTCTCCAAGAGGCATTGAT |
| | 30nt-g12 | TTTTATGAATGCTTCTCCAAGAGGCATTGA |
| | 30nt-g13 | CTTTTATGAATGCTTCTCCAAGAGGCATTG |
| | 30nt-g14 | CCTTTTATGAATGCTTCTCCAAGAGGCATT |
| | 30nt-g15 | CTTCTCCAAGAGGCATTGATATTCTCTGTT |
| | 30nt-g16 | GCTTCTCCAAGAGGCATTGATATTCTCTGT |
| | 30nt-g17 | TGCTTCTCCAAGAGGCATTGATATTCTCTG |
| | 30nt-g18 | AATGCTTCTCCAAGAGGCATTGATATTCTC |
| | 30nt-g19 | GAATGCTTCTCCAAGAGGCATTGATATTCT |
| | 30nt-g20 | TGAATGCTTCTCCAAGAGGCATTGATATTC |
| | 30nt-g21 | ATGAATGCTTCTCCAAGAGGCATTGATATT |
| | 30nt-g22 | ATGCTTCTCCAAGAGGCATTGATATTCTCT |

**Supplemental sequences AAV constructs' sequences of C1 to C4, E1 to E4 and M1 to M3**

C1 to C4 AAV constructs are CBh-driven mxABE. CBh promoter sequence is highlighted in red

E1 to E4 AAV constructs are EFS-driven mxABE. EFS promoter sequence is highlighted in green

M1 to M3 AAV constructs are MHCK7-driven mxABE. MHCK7 promoter sequence is highlighted in blue

>C1
CCTGCAGGCAGCTGCGCGCTCGCTCGCTCACTGAGGCCGCCCGGGCG
TCGGGCGACCTTTGGTCGCCCGGCCTCAGTGAGCGAGCGAGCGCGCA
GAGAGGGAGTGGCCAACTCCATCACTAGGGGTTCCTGCGGCCTCTAGAg
gagggcctatttcccatgattccttcatatttgcatatacgatacaaggctgttagagagataattggaattaatt
tgactgtaaacacaaagatattagtacaaaatacgtgacgtagaaagtaataatttcttgggtagtttgcagttt
taaaattatgttttaaaatggactatcatatgcttaccgtaacttgaaagtatttcgatttcttggctttatatatcttG
TGGAAAGGACGAAACACCGGCTGGAGCAGCCCCCGATTTGTGGGGTGA
TTACAGCgTCTGCAATATAAGCTGCCAACCACTTGTCAATGAATGTGAGGG
ACTCCctGCTGGAGCAGCCCCCGATTTGTGGGGTGATTACAGCTTTTTTTccg
ttacataacttacggtaaatggcccgcctggctgaccgcccaacgacccccgcccattgacgtcaatagta
acgccaatagggactttccattgacgtcaatgggtggagtatttacggtaaactgcccacttggcagtacatc
aagtgtatcatatgccaagtacgccccctattgacgtcaatgacggtaaatggcccgcctggcattGtgccc
agtacatgaccttatgggactttcctacttggcagtacatctacgtattagtcatcgctattaccatggtcgaggt
gagccccacgttctgcttcactctccccatctcccccccctccccacccccaattttgtatttatttattttttaattat
tttgtgcagcgatgggggcggggggggggggggggcgcgcgccaggcggggcggggcggggcgag
gggcggggcggggcgaggcggagaggtgcggcggcagccaatcagagcggcgcgctccgaaagttt
ccttttatggcgaggcggcggcggcggcggccctataaaaagcgaagcgcgcggcgggcgggagtcgc
tgcgacgctgccttcgccccgtgccccgctccgccgccgcctcgcgccgcccgccccggctctgactgacc
gcgttactcccacaggtgagcgggcgggacggcccttctcctccgggctgtaattagctgagcaagaggta
agggtttaagggatggttggttggtggggtattaatgtttaattacctggagcacctgcctgaaatcactttttttc
aggttGGaccggtGCCACCATGGCCCCCAAGAAGAAGaGGAAGGTGCTGAGC
ATGTACTGCCTGAAGGACAGCAGATTCACCAAGGCCTGGGATAAGCGGG
TGCTGCTGTTCAGAGACATCCTGGCCCAGCTGGGAAGAATCCCCGCCGA
GGCCTACGAGTACTACCACGGCGAGCAGGGTGATAAGAAGAGAGCTAAC
GACAATGAGGGCACAAATCCCAAGCGGCACAAGGACAAGTTCATCGAAT
TTGCACTGCACTACCTGGAAGCCCAGCACAGCGAGATCTGCTTCGGCAG
ACGCCACATCGTGCGGGAAGAGGCCGGCGCCGGCGATGAGCACAAGAA
GCACCGGACCAAGGGAAAGGTGGTGGTGGACTTCAGCAAGAAGGACGA
GGACCAGAGCTACTATATCTCCAAGAACAACGTGATCGTGCGGATCGACA
AGAACGCCGGCCCTAGAAGCTACCGGATGGGCCTGAACGAGCTGAAGTA
CCTCGTGCTGCTGAGCCTGCAGGGGAAGGGCGACGATGCCATCGCCAA
GCTGTACAGATACAGACAGCACGTGGAGAACATCCTGGATGTGGTGAAG
GTGACCGATAAGGATAACCACGTGTTCCTGCCCCGCTTCGTGCTGGAGC

AGCACGGCATCGGCAGAAAGGCCTTCAAGCAGCGGATCGATGGACGGG
TGAAGCACGTGCGGGGCGTGTGGGAGAAGAAGAAGGCCGCCACCAATG
AAATGACCCTGCACGAGAAGGCCAGAGACATCCTGCAGTACGTGAACGA
AAACTGCACCCGGTCCTTCAACCCTGGCGAATACAACAGACTGCTGGTG
TGCCTGGTGGGCAAGGACGTGGAGAACTTTCAGGCCGGCCTGAAGCGG
CTGCAGCTGGCCGAAAGGATCGATGGCCGGGTGTACTCCATCTTCGCCC
AGACCAGCACCATCAATGAGATGCACCAGGTGGTGTGCGACCAGATCCT
GAACCGGCTGTGCAGAATCGGCGACCAGAAGCTGTACGATTACGTGGGA
CTGGGCAAGAAGGACGAAATCGACTACAAGCAGAAGGTGGCCTGGTTCA
AGGAGCACATCAGCATCCGGAGAGGATTCCTGAGAAAGAAGTTCTGGTA
CGATAGCAAGAAGGGATTCGCAAAGCTGGTGGAGGAACACCTGGAGTCC
GGCGGCGGCCAGCGCGACGTGGGCCTGGACAAGAAGTACTACCACATC
GACGCCATCGGCAGATTCGAGGGCGCCAACCCCGCCCTGTACGAGACC
CTGGCCAGAGATCGGCTGTGCCTCATGATGGCCCAGTACTTCCTGGGCA
GCGTGAGAAAGGAACTGGGCAACAAGATTGTGTGGAGCAACGACAGCAT
CGAACTGCCTCCCAAGAAGAAGCGGAAGGTGGGTGGAGGCGGAGGTTC
TGGGGGAGGAGGTAGTGGCGGTGGTGGTTCAGGAGGCGGCGGAAGCca
gctgcatttaccgcaggttttagctgacgctgtctcacgcctggtcctgggtaagtttggtgacctgaccgaca
acttctcctcccctcacgctcgcagaaaagtgctggctggagtcgtcatgacaacaggcacagatgttaaa
gatgccaaggtgataagtgtttctacaggaACcaaatgtattaatggtgaatacatgagtgatcgtggccttg
cattaaatgactgccatgcagaaataatatctcggagatccttgctcagatttctttatacacaacttgagcttta
cttaaataacaaagatgatcaaaaaagatccatctttcagaaatcagagcgaggggggtttaggctgaag
gagaatgtccagtttcatctgtacatcagcacctctccctgtggagatgccagaatcttctcaccacatgagcc
aatcctggaagaaccagcagatagacacccaaatcgtaaagcaagaggacagctacggaccaaaata
gagtctggtcaggggacgattccagtgcgctccaatgcgagcatccaaacgtgggacggggtgctgcaa
ggggagcggctgctcaccatgtcctgcagtgacaagattgcacgctggaacgtggtgggcatccagggat
cactgctcagcattttcgtggagcccatttacttctcgagcatcatcctgggcagcctttaccacggggaccac
ctttccagggccatgtaccagcggatctccaacatagaggacctgccacctctctacaccctcaacaagcct
ttgctcagtggcatcagcaatgcagaagcacggcagccagggaaggcccccaacttcagtgtcaactgg
acggtaggcgactccgctattgaggtcatcaacgccacgactgggaaggatgagctgggccgcgcgtcc
cgcctgtgtaagcACGCGTTGTACTGTCGCTGGATGCGTGTGCACGGCAAGGT
TCCCTCCCACTTACTACGCTCCAAGATTACCAAGCCCAACGTGTACCATG
AGTCCAAGCTGGCGGCAAAGGAGTACCAGGCCGCCAAGGCGCGTCTGT
TCACAGCCTTCATCAAGGCGGGGCTGGGGGCCTGGGTGGAGAAGCCCA
CCGAGCAGGACCAGTTCTCACTCACGtacccatacgatgttccagattacgcttatccta
cgacgtgcctgattatgcatacccatatgatgtccccgactatgccGCGGCCGCTGATTACAAA
GACGATGACGATAAGtaaATCGAATTCCGCTCGAGATAATCAACCTCTGGA
TTACAAAATTTGTGAAAGATTGACTGGTATTCTTAACTATGTTGCTCCTTTT
ACGCTATGTGGATACGCTGCTTTAATGCCTTTGTATCATGCTATTGCTTCCC
GTATGGCTTTCATTTTCTCCTCCTTGTATAAATCCTGGTTAGTTCTTGCCAC
GGCGGAACTCATCGCCGCCTGCCTTGCCCGCTGCTGGACAGGGGCTCG
GCTGTTGGGCACTGACAATTCCGTGGTGTTTATTTGTGAAATTTGTGATGC
TATTGCTTTATTTGTAACCATCTAGCTTTATTTGTGAAATTTGTGATGCTATT
GCTTTATTTGTAACCATTATAAGCTGCAATAAACAAGTTAACAACAACAATT

GCATTCATTTTATGTTTCAGGTTCAGGGGGAGATGTGGGAGGTTTTTTAAA
GCGGCCGCCggccgagGGCCGCAGGAACCCCTAGTGATGGAGTTGGCCA
CTCCCTCTCTGCGCGCTCGCTCGCTCACTGAGGCCGGGCGACCAAAGG
TCGCCCGACGCCCGGGCTTTGCCCGGGCGGCCTCAGTGAGCGAGCGA
GCGCGCAGCTGCCTGCAGGGGCGCCTGATGCGGTATTTTCTCCTTACGC
ATCTGTGCGGTATTTCACACCGCATACGTCAAAGCAACCATAGTACGCGC
CCTGTAGCGGCGCATTAAGCGCGGCGGGTGTGGTGGTTACGCGCAGCG
TGACCGCTACACTTGCCAGCGCCTTAGCGCCCGCTCCTTTCGCTTTCTTC
CCTTCCTTTCTCGCCACGTTCGCCGGCTTTCCCCGTCAAGCTCTAAATCG
GGGGCTCCCTTTAGGGTTCCGATTTAGTGCTTTACGGCACCTCGACCCCA
AAAAACTTGATTTGGGTGATGGTTCACGTAGTGGGCCATCGCCCTGATAG
ACGGTTTTTCGCCCTTTGACGTTGGAGTCCACGTTCTTTAATAGTGGACT
CTTGTTCCAAACTGGAACAACACTCAACTCTATCTCGGGCTATTCTTTTGA
TTTATAAGGGATTTTGCCGATTTCGGTCTATTGGTTAAAAAATGAGCTGATT
TAACAAAAATTTAACGCGAATTTTAACAAAATATTAACGTTTACAATTTTATG
GTGCACTCTCAGTACAATCTGCTCTGATGCCGCATAGTTAAGCCAGCCCC
GACACCCGCCAACACCCGCTGACGCGCCCTGACGGGCTTGTCTGCTCC
CGGCATCCGCTTACAGACAAGCTGTGACCGTCTccgggagctgcatgtgtcagaggt
tttcaccgtcatcaccgaaacgcgcgagACGAAAGGGCCTCGTGATACGCCTATTTTTA
TAGGTTAATGTCATGATAATAATGGTTTCTTAGACGTCAGGTGGCACTTTTC
GGGGAAATGTGCGCGGAACCCCTATTTGTTTATTTTTCTAAATACATTCAAA
TATGTATCCGCTCATGAGACAATAACCCTGATAAATGCTTCAATAATATTGA
AAAAGGAAGAGTATGAGTATTCAACATTTCCGTGTCGCCCTTATTCCCTTT
TTTGCGGCATTTTGCCTTCCTGTTTTTGCTCACCCAGAAACGCTGGTGAA
AGTAAAAGATGCTGAAGATCAGTTGGGTGCACGAGTGGGTTACATCGAAC
TGGATCTCAACAGCGGTAAGATCCTTGAGAGTTTTCGCCCCGAAGAACGT
TTTCCAATGATGAGCACTTTTAAAGTTCTGCTATGTGGCGCGGTATTATCC
CGTATTGACGCCGGGCAAGAGCAACTCGGTCGCCGCATACACTATTCTCA
GAATGACTTGGTTGAGTACTCACCAGTCACAGAAAAGCATCTTACGGATG
GCATGACAGTAAGAGAATTATGCAGTGCTGCCATAACCATGAGTGATAACA
CTGCGGCCAACTTACTTCTGACAACGATCGGAGGACCGAAGGAGCTAAC
CGCTTTTTTGCACAACATGGGGGATCATGTAACTCGCCTTGATCGTTGGG
AACCGGAGCTGAATGAAGCCATACCAAACGACGAGCGTGACACCACGAT
GCCTGTAGCAATGGCAACAACGTTGCGCAAACTATTAACTGGCGAACTAC
TTACTCTAGCTTCCCGGCAACAATTAATAGACTGGATGGAGGCGGATAAA
GTTGCAGGACCACTTCTGCGCTCGGCCCTTCCGGCTGGCTGGTTTATTG
CTGATAAATCTGGAGCCGGTGAGCGTGGAAGCCGCGGTATCATTGCAGC
ACTGGGGCCAGATGGTAAGCCCTCCCGTATCGTAGTTATCTACACGACGG
GGAGTCAGGCAACTATGGATGAACGAAATAGACAGATCGCTGAGATAGGT
GCCTCACTGATTAAGCATTGGTAACTGTCAGACCAAGTTTACTCATATATAC
TTTAGATTGATTTAAAACTTCATTTTTAATTTAAAAGGATCTAGGTGAAGATC
CTTTTTGATAATCTCATGACCAAAATCCCTTAACGTGAGTTTTCGTTCCACT
GAGCGTCAGACCCCGTAGAAAAGATCAAAGGATCTTCTTGAGATCCTTTT
TTTCTGCGCGTAATCTGCTGCTTGCAAACAAAAAAACCACCGCTACCAGC

GGTGGTTTGTTTGCCGGATCAAGAGCTACCAACTCTTTTTCCGAAGGTAA
CTGGCTTCAGCAGAGCGCAGATACCAAATACTGTTCTTCTAGTGTAGCCG
TAGTTAGGCCACCACTTCAAGAACTCTGTAGCACCGCCTACATACCTCGC
TCTGCTAATCCTGTTACCAGTGGCTGCTGCCAGTGGCGATAAGTCGTGTC
TTACCGGGTTGGACTCAAGACGATAGTTACCGGATAAGGCGCAGCGGTC
GGGCTGAACGGGGGGTTCGTGCACACAGCCCAGCTTGGAGCGAACGAC
CTACACCGAACTGAGATACCTACAGCGTGAGCTATGAGAAAGCGCCACG
CTTCCCGAAGGGAGAAAGGCGGACAGGTATCCGGTAAGCGGCAGGGTC
GGAACAGGAGAGCGCACGAGGGAGCTTCCAGGGGGAAACGCCTGGTAT
CTTTATAGTCCTGTCGGGTTTCGCCACCTCTGACTTGAGCGTCGATTTTT
GTGATGCTCGTCAGGGGGGCGGAGCCTATGGAAAAACGCCAGCAACGC
GGCCTTTTTACGGTTCCTGGCCTTTTGCTGGCCTTTTGCTCACATGT

>C2
CCTGCAGGCAGCTGCGCGCTCGCTCGCTCACTGAGGCCGCCCGGGCG
TCGGGCGACCTTTGGTCGCCCGGCCTCAGTGAGCGAGCGAGCGCGCA
GAGAGGGAGTGGCCAACTCCATCACTAGGGGTTCCTGCGGCCTCTAGAg
gagggcctatttcccatgattccttcatatttgcatatacgatacaaggctgttagagagataattggaattaatt
tgactgtaaacacaaagatattagtacaaaatacgtgacgtagaaagtaataatttcttgggtagtttgcagttt
taaaattatgttttaaaatggactatcatatgcttaccgtaacttgaaagtatttcgatttcttggctttatatatcttG
TGGAAAGGACGAAACACCGgCTGGAGCAGCCCCCGATTTGTGGGGTGAT
TACAGCGTCTGCAATATAAGCTGCCAACCACTTGTCAATGAATGTGAGGG
ACTCCTGCTGGAGCAGCCCCCGATTTGTGGGGTGATTACAGCTTTTTTTcc
gttacataacttacggtaaatggcccgcctggctgaccgcccaacgacccccgcccattgacgtcaatagt
aacgccaatagggactttccattgacgtcaatgggtggagtatttacggtaaactgcccacttggcagtacat
caagtgtatcatatgccaagtacgccccctattgacgtcaatgacggtaaatggcccgcctggcattGtgcc
cagtacatgaccttatgggactttcctacttggcagtacatctacgtattagtcatcgctattaccatggtcgagg
tgagccccacgttctgcttcactctccccatctcccccccctccccacccccaattttgtatttatttattttttaatta
ttttgtgcagcgatgggggcgggggggggggggggcgcgcgccaggcggggcggggcggggcgag
gggcggggcggggcgaggcggagaggtgcggcggcagccaatcagagcggcgcgctccgaaagttt
ccttttatggcgaggcggcggcggcggcggccctataaaaagcgaagcgcgcggcgggcgggagtcgc
tgcgacgctgccttcgccccgtgccccgctccgccgccgcctcgcgccgcccgccccggctctgactgacc
gcgttactcccacaggtgagcgggcgggacggcccttctcctccgggctgtaattagctgagcaagaggta
agggtttaagggatggttggttggtggggtattaatgtttaattacctggagcacctgcctgaaatcactttttttc
aggttGGaccggtGCCACCATGGCCCCCAAGAAGAAGaGGAAGGTGCTGAGC
ATGTACTGCCTGAAGGACAGCAGATTCACCAAGGCCTGGGATAAGCGG
TGCTGCTGTTCAGAGACATCCTGGCCCAGCTGGGAAGAATCCCCGCCGA
GGCCTACGAGTACTACCACGGCGAGCAGGGTGATAAGAAGAGAGCTAAC
GACAATGAGGGCACAAATCCCAAGCGGCACAAGGACAAGTTCATCGAAT
TTGCACTGCACTACCTGGAAGCCCAGCACAGCGAGATCTGCTTCGGCAG
ACGCCACATCGTGCGGAAGAGGCCGGCGCCGGCGATGAGCACAAGAA
GCACCGGACCAAGGGAAAGGTGGTGGTGGACTTCAGCAAGAAGGACGA
GGACCAGAGCTACTATATCTCCAAGAACAACGTGATCGTGCGGATCGACA
AGAACGCCGGCCCTAGAAGCTACCGGATGGGCCTGAACGAGCTGAAGTA

CCTCGTGCTGCTGAGCCTGCAGGGGAAGGGCGACGATGCCATCGCCAA
GCTGTACAGATACAGACAGCACGTGGAGAACATCCTGGATGTGGTGAAG
GTGACCGATAAGGATAACCACGTGTTCCTGCCCCGCTTCGTGCTGGAGC
AGCACGGCATCGGCAGAAAGGCCTTCAAGCAGCGGATCGATGGACGGG
TGAAGCACGTGCGGGGCGTGTGGGAGAAGAAGAAGGCCGCCACCAATG
AAATGACCCTGCACGAGAAGGCCAGAGACATCCTGCAGTACGTGAACGA
AAACTGCACCCGGTCCTTCAACCCTGGCGAATACAACAGACTGCTGGTG
TGCCTGGTGGGCAAGGACGTGGAGAACTTTCAGGCCGGCCTGAAGCGG
CTGCAGCTGGCCGAAAGGATCGATGGCCGGGTGTACTCCATCTTCGCCC
AGACCAGCACCATCAATGAGATGCACCAGGTGGTGTGCGACCAGATCCT
GAACCGGCTGTGCAGAATCGGCGACCAGAAGCTGTACGATTACGTGGGA
CTGGGCAAGAAGGACGAAATCGACTACAAGCAGAAGGTGGCCTGGTTCA
AGGAGCACATCAGCATCCGGAGAGGATTCCTGAGAAAGAAGTTCTGGTA
CGATAGCAAGAAGGGATTCGCAAAGCTGGTGGAGGAACACCTGGAGTCC
GGCGGCGGCCAGCGCGACGTGGGCCTGGACAAGAAGTACTACCACATC
GACGCCATCGGCAGATTCGAGGGCGCCAACCCCGCCCTGTACGAGACC
CTGGCCAGAGATCGGCTGTGCCTCATGATGGCCCAGTACTTCCTGGGCA
GCGTGAGAAAGGAACTGGGCAACAAGATTGTGTGGAGCAACGACAGCAT
CGAACTGCCTCCCAAGAAGAAGCGGAAGGTGGGTGGAGGCGGAGGTTC
TGGGGGAGGAGGTAGTGGCGGTGGTGGTTCAGGAGGCGGCGGAAGCca
gctgcatttaccgcaggttttagctgacgctgtctcacgcctggtcctgggtaagtttggtgacctgaccgaca
acttctcctcccctcacgctcgcagaaaagtgctggctggagtcgtcatgacaacaggcacagatgttaaa
gatgccaaggtgataagtgtttctacaggaACcaaatgtattaatggtgaatacatgagtgatcgtggccttg
cattaaatgactgccatgcagaaataatatctcggagatccttgctcagatttctttatacacaacttgagcttta
cttaaataacaaagatgatcaaaaaagatccatctttcagaaatcagagcgagggggtttaggctgaag
gagaatgtccagtttcatctgtacatcagcacctctccctgtggagatgccagaatcttctcaccacatgagcc
aatcctggaagaaccagcagatagacacccaaatcgtaaagcaagaggacagctacggaccaaaata
gagtctggtcaggggacgattccagtgcgctccaatgcgagcatccaaacgtgggacggggtgctgcaa
ggggagcggctgctcaccatgtcctgcagtgacaagattgcacgctggaacgtggtgggcatccagggat
cactgctcagcattttcgtggagcccatttacttctcgagcatcatcctgggcagcctttaccacggggaccac
ctttccagggccatgtaccagcggatctccaacatagaggacctgccacctctctacaccctcaacaagcct
ttgctcagtggcatcagcaatgcagaagcacggcagccagggaaggcccccaacttcagtgtcaactgg
acggtaggcgactccgctattgaggtcatcaacgccacgactgggaaggatgagctgggccgcgcgtcc
cgcctgtgtaagcACGCGTTGTACTGTCGCTGGATGCGTGTGCACGGCAAGGT
TCCCTCCCACTTACTACGCTCCAAGATTACCAAGCCCAACGTGTACCATG
AGTCCAAGCTGGCGGCAAAGGAGTACCAGGCCGCCAAGGCGCGTCTGT
TCACAGCCTTCATCAAGGCGGGGCTGGGGGCCTGGGTGGAGAAGCCCA
CCGAGCAGGACCAGTTCTCACTCACGCCCAAGAAGAAGaGGAAGGTGG
GATCCtacccatacgatgttccagattacgcttatccctacgacgtgcctgattatgcatacccatatgatgt
ccccgactatgccGCGGCCGCTGATTACAAAGACGATGACGATAAGtaaGTCGA
CAACTTGTTTATTGCAGCTTATAATGGTTACAAATAAAGCAATAGCATCACA
AATTTCACAAATAAAGCATTTTTTTCACTGCATTCTAGTTGTGGTTTGTCCA
AACTCATCAATGTATCTTATCATGTCTGGATCGCggccgagGGCCGCAGGAA
CCCCTAGTGATGGAGTTGGCCACTCCCTCTCTGCGCGCTCGCTCGCTCA

CTGAGGCCGGGCGACCAAAGGTCGCCCGACGCCCGGGCTTTGCCCGG
GCGGCCTCAGTGAGCGAGCGAGCGCGCAGCTGCCTGCAGGGGCGCCT
GATGCGGTATTTTCTCCTTACGCATCTGTGCGGTATTTCACACCGCATACG
TCAAAGCAACCATAGTACGCGCCCTGTAGCGGCGCATTAAGCGCGGCGG
GTGTGGTGGTTACGCGCAGCGTGACCGCTACACTTGCCAGCGCCTTAGC
GCCCGCTCCTTTCGCTTTCTTCCCTTCCTTTCTCGCCACGTTCGCCGGCT
TTCCCCGTCAAGCTCTAAATCGGGGGCTCCCTTTAGGGTTCCGATTTAGT
GCTTTACGGCACCTCGACCCCAAAAAACTTGATTTGGGTGATGGTTCACG
TAGTGGGCCATCGCCCTGATAGACGGTTTTTCGCCCTTTGACGTTGGAGT
CCACGTTCTTTAATAGTGGACTCTTGTTCCAAACTGGAACAACACTCAACT
CTATCTCGGGCTATTCTTTTGATTTATAAGGGATTTTGCCGATTTCGGTCTA
TTGGTTAAAAAATGAGCTGATTTAACAAAAATTTAACGCGAATTTTAACAAA
ATATTAACGTTTACAATTTTATGGTGCACTCTCAGTACAATCTGCTCTGATG
CCGCATAGTTAAGCCAGCCCCGACACCCGCCAACACCCGCTGACGCGC
CCTGACGGGCTTGTCTGCTCCCGGCATCCGCTTACAGACAAGCTGTGAC
CGTCTccgggagctgcatgtgtcagaggttttcaccgtcatcaccgaaacgcgcgagACGAAAGG
GCCTCGTGATACGCCTATTTTTATAGGTTAATGTCATGATAATAATGGTTTCT
TAGACGTCAGGTGGCACTTTTCGGGGAAATGTGCGCGGAACCCCTATTT
GTTTATTTTTCTAAATACATTCAAATATGTATCCGCTCATGAGACAATAACCC
TGATAAATGCTTCAATAATATTGAAAAAGGAAGAGTATGAGTATTCAACATT
TCCGTGTCGCCCTTATTCCCTTTTTTGCGGCATTTTGCCTTCCTGTTTTTG
CTCACCCAGAAACGCTGGTGAAAGTAAAAGATGCTGAAGATCAGTTGGG
TGCACGAGTGGGTTACATCGAACTGGATCTCAACAGCGGTAAGATCCTTG
AGAGTTTTCGCCCCGAAGAACGTTTTCCAATGATGAGCACTTTTAAAGTT
CTGCTATGTGGCGCGGTATTATCCCGTATTGACGCCGGGCAAGAGCAACT
CGGTCGCCGCATACACTATTCTCAGAATGACTTGGTTGAGTACTCACCAG
TCACAGAAAAGCATCTTACGGATGGCATGACAGTAAGAGAATTATGCAGT
GCTGCCATAACCATGAGTGATAACACTGCGGCCAACTTACTTCTGACAAC
GATCGGAGGACCGAAGGAGCTAACCGCTTTTTTGCACAACATGGGGGAT
CATGTAACTCGCCTTGATCGTTGGGAACCGGAGCTGAATGAAGCCATACC
AAACGACGAGCGTGACACCACGATGCCTGTAGCAATGGCAACAACGTTG
CGCAAACTATTAACTGGCGAACTACTTACTCTAGCTTCCCGGCAACAATTA
ATAGACTGGATGGAGGCGGATAAAGTTGCAGGACCACTTCTGCGCTCGG
CCCTTCCGGCTGGCTGGTTTATTGCTGATAAATCTGGAGCCGGTGAGCGT
GGAAGCCGCGGTATCATTGCAGCACTGGGGCCAGATGGTAAGCCCTCCC
GTATCGTAGTTATCTACACGACGGGGAGTCAGGCAACTATGGATGAACGA
AATAGACAGATCGCTGAGATAGGTGCCTCACTGATTAAGCATTGGTAACTG
TCAGACCAAGTTTACTCATATATACTTTAGATTGATTTAAAACTTCATTTTTAA
TTTAAAAGGATCTAGGTGAAGATCCTTTTTGATAATCTCATGACCAAAATCC
CTTAACGTGAGTTTTCGTTCCACTGAGCGTCAGACCCCGTAGAAAAGATC
AAAGGATCTTCTTGAGATCCTTTTTTTCTGCGCGTAATCTGCTGCTTGCAA
ACAAAAAAACCACCGCTACCAGCGGTGGTTTGTTTGCCGGATCAAGAGC
TACCAACTCTTTTTCCGAAGGTAACTGGCTTCAGCAGAGCGCAGATACCA
AATACTGTTCTTCTAGTGTAGCCGTAGTTAGGCCACCACTTCAAGAACTCT

GTAGCACCGCCTACATACCTCGCTCTGCTAATCCTGTTACCAGTGGCTGC
TGCCAGTGGCGATAAGTCGTGTCTTACCGGGTTGGACTCAAGACGATAGT
TACCGGATAAGGCGCAGCGGTCGGGCTGAACGGGGGGTTCGTGCACAC
AGCCCAGCTTGGAGCGAACGACCTACACCGAACTGAGATACCTACAGCG
TGAGCTATGAGAAAGCGCCACGCTTCCCGAAGGGAGAAAGGCGGACAG
GTATCCGGTAAGCGGCAGGGTCGGAACAGGAGAGCGCACGAGGGAGCT
TCCAGGGGGAAACGCCTGGTATCTTTATAGTCCTGTCGGGTTTCGCCACC
TCTGACTTGAGCGTCGATTTTTGTGATGCTCGTCAGGGGGGCGGAGCCT
ATGGAAAAACGCCAGCAACGCGGCCTTTTTACGGTTCCTGGCCTTTTGCT
GGCCTTTTGCTCACATGT

>C3
CCTGCAGGCAGCTGCGCGCTCGCTCGCTCACTGAGGCCGCCCGGGCG
TCGGGCGACCTTTGGTCGCCCGGCCTCAGTGAGCGAGCGAGCGCGCA
GAGAGGGAGTGGCCAACTCCATCACTAGGGGTTCCTGCGGCCTCTAGAg
gagggcctatttcccatgattccttcatatttgcatatacgatacaaggctgttagagagataattggaattaatt
tgactgtaaacacaaagatattagtacaaaatacgtgacgtagaaagtaataatttcttgggtagtttgcagttt
taaaattatgttttaaaatggactatcatatgcttaccgtaacttgaaagtatttcgatttcttggctttatatatcttG
TGGAAAGGACGAAACACCGGCTGGAGCAGCCCCCGATTTGTGGGGTGA
TTACAGCGTCTGCAATATAAGCTGCCAACCACTTGTCAATGAATGTGAGG
GACTCCTGCTGGAGCAGCCCCCGATTTGTGGGGTGATTACAGCTTTTTTT
ccgttacataacttacggtaaatggcccgcctggctgaccgcccaacgacccccgcccattgacgtcaata
gtaacgccaatagggactttccattgacgtcaatgggtggagtatttacggtaaactgcccacttggcagtac
atcaagtgtatcatatgccaagtacgccccctattgacgtcaatgacggtaaatggcccgcctggcattGtg
cccagtacatgaccttatgggactttcctacttggcagtacatctacgtattagtcatcgctattaccatggtcga
ggtgagccccacgttctgcttcactctccccatctcccccccctccccacccccaattttgtatttatttattttttaa
ttattttgtgcagcgatgggggcgggggggggggggggcgcgcgccaggcggggcggggcggggcg
aggggcggggcggggcgaggcggagaggtgcggcggcagccaatcagagcggcgcgctccgaaag
tttccttttatggcgaggcggcggcggcggcggccctataaaaagcgaagcgcgcggcgggcgggagtc
gctgcgacgctgccttcgccccgtgccccgctccgccgccgcctcgcgccgcccgccccggctctgactga
ccgcgttactcccacaggtgagcgggcgggacggcccttctcctccgggctgtaattagctgagcaagag
gtaagggtttaagggatggttggttggtggggtattaatgtttaattacctggagcacctgcctgaaatcactttttt
ttcaggttGGaccggtGCCACCATGGCCCCCAAGAAGAAGaGGAAGGTGCTGA
GCATGTACTGCCTGAAGGACAGCAGATTCACCAAGGCCTGGGATAAGCG
GGTGCTGCTGTTCAGAGACATCCTGGCCCAGCTGGGAAGAATCCCCGCC
GAGGCCTACGAGTACTACCACGGCGAGCAGGGTGATAAGAAGAGAGCTA
ACGACAATGAGGGCACAAATCCCAAGCGGCACAAGGACAAGTTCATCGA
ATTTGCACTGCACTACCTGGAAGCCCAGCACAGCGAGATCTGCTTCGGC
AGACGCCACATCGTGCGGGAAGAGGCCGGCGCCGGCGATGAGCACAAG
AAGCACCGGACCAAGGGAAAGGTGGTGGACTTCAGCAAGAAGGAC
GAGGACCAGAGCTACTATATCTCCAAGAACAACGTGATCGTGCGGATCGA
CAAGAACGCCGGCCCTAGAAGCTACCGGATGGGCCTGAACGAGCTGAA
GTACCTCGTGCTGCTGAGCCTGCAGGGGAAGGGCGACGATGCCATCGC
CAAGCTGTACAGATACAGACAGCACGTGGAGAACATCCTGGATGTGGTG

AAGGTGACCGATAAGGATAACCACGTGTTCCTGCCCCGCTTCGTGCTGG
AGCAGCACGGCATCGGCAGAAAGGCCTTCAAGCAGCGGATCGATGGAC
GGGTGAAGCACGTGCGGGGCGTGTGGGAGAAGAAGAAGGCCGCCACC
AATGAAATGACCCTGCACGAGAAGGCCAGAGACATCCTGCAGTACGTGA
ACGAAAACTGCACCCGGTCCTTCAACCCTGGCGAATACAACAGACTGCT
GGTGTGCCTGGTGGGCAAGGACGTGGAGAACTTTCAGGCCGGCCTGAA
GCGGCTGCAGCTGGCCGAAAGGATCGATGGCCGGGTGTACTCCATCTTC
GCCCAGACCAGCACCATCAATGAGATGCACCAGGTGGTGTGCGACCAGA
TCCTGAACCGGCTGTGCAGAATCGGCGACCAGAAGCTGTACGATTACGT
GGGACTGGGCAAGAAGGACGAAATCGACTACAAGCAGAAGGTGGCCTG
GTTCAAGGAGCACATCAGCATCCGGAGAGGATTCCTGAGAAAGAAGTTC
TGGTACGATAGCAAGAAGGGATTCGCAAAGCTGGTGGAGGAACACCTGG
AGTCCGGCGGCGGCCAGCGCGACGTGGGCCTGGACAAGAAGTACTACC
ACATCGACGCCATCGGCAGATTCGAGGGCGCCAACCCCGCCCTGTACGA
GACCCTGGCCAGAGATCGGCTGTGCCTCATGATGGCCCAGTACTTCCTG
GGCAGCGTGAGAAAGGAACTGGGCAACAAGATTGTGTGGAGCAACGAC
AGCATCGAACTGCCTCCCAAGAAGAAGCGGAAGGTGGGTGGAGGCGGA
GGTTCTGGGGGAGGAGGTAGTGGCGGTGGTGGTTCAGGAGGCGGCGG
AAGCcagctgcatttaccgcaggttttagctgacgctgtctcacgcctggtcctgggtaagtttggtgacctg
accgacaacttctcctcccctcacgctcgcagaaaagtgctggctggagtcgtcatgacaacaggcacag
atgttaaagatgccaaggtgataagtgtttctacaggaACcaaatgtattaatggtgaatacatgagtgatc
gtggccttgcattaaatgactgccatgcagaaataatatctcggagatccttgctcagatttctttatacacaac
ttgagctttacttaaataacaaagatgatcaaaaaagatccatctttcagaaatcagagcgaggggggttta
ggctgaaggagaatgtccagtttcatctgtacatcagcacctctccctgtggagatgccagaatcttctcacc
acatgagccaatcctggaagaaccagcagatagacacccaaatcgtaaagcaagaggacagctacgg
accaaaatagagtctggtcaggggacgattccagtgcgctccaatgcgagcatccaaacgtgggacggg
gtgctgcaaggggagcggctgctcaccatgtcctgcagtgacaagattgcacgctggaacgtggtgggca
tccagggatcactgctcagcattttcgtggagcccatttacttctcgagcatcatcctgggcagcctttaccacg
gggaccacctttccagggccatgtaccagcggatctccaacatagaggacctgccacctctctacaccctc
aacaagcctttgctcagtggcatcagcaatgcagaagcacggcagccagggaaggcccccaacttcagt
gtcaactggacggtaggcgactccgctattgaggtcatcaacgccacgactgggaaggatgagctgggcc
gcgcgtcccgcctgtgtaagcACGCGTTGTACTGTCGCTGGATGCGTGTGCACGG
CAAGGTTCCCTCCCACTTACTACGCTCCAAGATTACCAAGCCCAACGTGT
ACCATGAGTCCAAGCTGGCGGCAAAGGAGTACCAGGCCGCCAAGGCGC
GTCTGTTCACAGCCTTCATCAAGGCGGGGCTGGGGGCCTGGGTGGAGA
AGCCCACCGAGCAGGACCAGTTCTCACTCACGAAAAGGCCGGCGGCCA
CGAAAAAGGCCGGCCAGGCAAAAAAGAAAAAGtacccatacgatgttccagattacg
cttatccctacgacgtgcctgattatgcatacccatatgatgtccccgactatgccGCGGCCGCTGAT
TACAAAGACGATGACGATAAGtaaATCGAATTCCGCTCGAGATAATCAACCT
CTGGATTACAAAATTTGTGAAAGATTGACTGGTATTCTTAACTATGTTGCTC
CTTTTACGCTATGTGGATACGCTGCTTTAATGCCTTTGTATCATGCTATTGC
TTCCCGTATGGCTTTCATTTTCTCCTCCTTGTATAAATCCTGGTTAGTTCTT
GCCACGGCGGAACTCATCGCCGCCTGCCTTGCCCGCTGCTGGACAGGG
GCTCGGCTGTTGGGCACTGACAATTCCGTGGTGTTTATTTGTGAAATTTG

TGATGCTATTGCTTTATTTGTAACCATCTAGCTTTATTTGTGAAATTTGTGAT
GCTATTGCTTTATTTGTAACCATTATAAGCTGCAATAAACAAGTTAACAACA
ACAATTGCATTCATTTTATGTTTCAGGTTCAGGGGGAGATGTGGGAGGTTT
TTTAAAGCGGCCGCCggccgagGGCCGCAGGAACCCCTAGTGATGGAGTT
GGCCACTCCCTCTCTGCGCGCTCGCTCGCTCACTGAGGCCGGGCGACC
AAAGGTCGCCCGACGCCCGGGCTTTGCCCGGGCGGCCTCAGTGAGCG
AGCGAGCGCGCAGCTGCCTGCAGGGGCGCCTGATGCGGTATTTTCTCCT
TACGCATCTGTGCGGTATTTCACACCGCATACGTCAAAGCAACCATAGTAC
GCGCCCTGTAGCGGCGCATTAAGCGCGGCGGGTGTGGTGGTTACGCGC
AGCGTGACCGCTACACTTGCCAGCGCCTTAGCGCCCGCTCCTTTCGCTT
TCTTCCCTTCCTTTCTCGCCACGTTCGCCGGCTTTCCCCGTCAAGCTCTA
AATCGGGGGCTCCCTTTAGGGTTCCGATTTAGTGCTTTACGGCACCTCGA
CCCCAAAAAACTTGATTTGGGTGATGGTTCACGTAGTGGGCCATCGCCCT
GATAGACGGTTTTTCGCCCTTTGACGTTGGAGTCCACGTTCTTTAATAGTG
GACTCTTGTTCCAAACTGGAACAACACTCAACTCTATCTCGGGCTATTCTT
TTGATTTATAAGGGATTTTGCCGATTTCGGTCTATTGGTTAAAAAATGAGCT
GATTTAACAAAAATTTAACGCGAATTTTAACAAAATATTAACGTTTACAATTT
TATGGTGCACTCTCAGTACAATCTGCTCTGATGCCGCATAGTTAAGCCAG
CCCCGACACCCGCCAACACCCGCTGACGCGCCCTGACGGGCTTGTCTG
CTCCCGGCATCCGCTTACAGACAAGCTGTGACCGTCTccgggagctgcatgtgtc
agaggttttcaccgtcatcaccgaaacgcgcgagACGAAAGGGCCTCGTGATACGCCTAT
TTTTATAGGTTAATGTCATGATAATAATGGTTTCTTAGACGTCAGGTGGCAC
TTTTCGGGGAAATGTGCGCGGAACCCCTATTTGTTTATTTTTCTAAATACAT
TCAAATATGTATCCGCTCATGAGACAATAACCCTGATAAATGCTTCAATAATA
TTGAAAAAGGAAGAGTATGAGTATTCAACATTTCCGTGTCGCCCTTATTCC
CTTTTTTGCGGCATTTTGCCTTCCTGTTTTTGCTCACCCAGAAACGCTGG
TGAAAGTAAAAGATGCTGAAGATCAGTTGGGTGCACGAGTGGGTTACATC
GAACTGGATCTCAACAGCGGTAAGATCCTTGAGAGTTTTCGCCCCGAAG
AACGTTTTCCAATGATGAGCACTTTTAAAGTTCTGCTATGTGGCGCGGTAT
TATCCCGTATTGACGCCGGGCAAGAGCAACTCGGTCGCCGCATACACTAT
TCTCAGAATGACTTGGTTGAGTACTCACCAGTCACAGAAAAGCATCTTAC
GGATGGCATGACAGTAAGAGAATTATGCAGTGCTGCCATAACCATGAGTG
ATAACACTGCGGCCAACTTACTTCTGACAACGATCGGAGGACCGAAGGA
GCTAACCGCTTTTTTGCACAACATGGGGGATCATGTAACTCGCCTTGATC
GTTGGGAACCGGAGCTGAATGAAGCCATACCAAACGACGAGCGTGACAC
CACGATGCCTGTAGCAATGGCAACAACGTTGCGCAAACTATTAACTGGCG
AACTACTTACTCTAGCTTCCCGGCAACAATTAATAGACTGGATGGAGGCG
GATAAAGTTGCAGGACCACTTCTGCGCTCGGCCCTTCCGGCTGGCTGGT
TTATTGCTGATAAATCTGGAGCCGGTGAGCGTGGAAGCCGCGGTATCATT
GCAGCACTGGGGCCAGATGGTAAGCCCTCCCGTATCGTAGTTATCTACAC
GACGGGGAGTCAGGCAACTATGGATGAACGAAATAGACAGATCGCTGAG
ATAGGTGCCTCACTGATTAAGCATTGGTAACTGTCAGACCAAGTTTACTCA
TATATACTTTAGATTGATTTAAAACTTCATTTTTAATTTAAAAGGATCTAGGTG
AAGATCCTTTTTGATAATCTCATGACCAAAATCCCTTAACGTGAGTTTTCGT

TCCACTGAGCGTCAGACCCCGTAGAAAAGATCAAAGGATCTTCTTGAGAT
CCTTTTTTTCTGCGCGTAATCTGCTGCTTGCAAACAAAAAAACCACCGCTA
CCAGCGGTGGTTTGTTTGCCGGATCAAGAGCTACCAACTCTTTTTCCGAA
GGTAACTGGCTTCAGCAGAGCGCAGATACCAAATACTGTTCTTCTAGTGT
AGCCGTAGTTAGGCCACCACTTCAAGAACTCTGTAGCACCGCCTACATAC
CTCGCTCTGCTAATCCTGTTACCAGTGGCTGCTGCCAGTGGCGATAAGTC
GTGTCTTACCGGGTTGGACTCAAGACGATAGTTACCGGATAAGGCGCAG
CGGTCGGGCTGAACGGGGGGTTCGTGCACACAGCCCAGCTTGGAGCGA
ACGACCTACACCGAACTGAGATACCTACAGCGTGAGCTATGAGAAAGCG
CCACGCTTCCCGAAGGGAGAAAGGCGGACAGGTATCCGGTAAGCGGCA
GGGTCGGAACAGGAGAGCGCACGAGGGAGCTTCCAGGGGGAAACGCC
TGGTATCTTTATAGTCCTGTCGGGTTTCGCCACCTCTGACTTGAGCGTCG
ATTTTTGTGATGCTCGTCAGGGGGGCGGAGCCTATGGAAAAACGCCAGC
AACGCGGCCTTTTTACGGTTCCTGGCCTTTTGCTGGCCTTTTGCTCACAT
GT

>C4
CCTGCAGGCAGCTGCGCGCTCGCTCGCTCACTGAGGCCGCCCGGGCG
TCGGGCGACCTTTGGTCGCCCGGCCTCAGTGAGCGAGCGAGCGCGCA
GAGAGGGAGTGGCCAACTCCATCACTAGGGGTTCCTGCGGCCTCTAGAg
agggcctatttcccatgattccttcatatttgcatatacgatacaaggctgttagagagataattggaattaattt
gactgtaaacacaaagatattagtacaaaatacgtgacgtagaaagtaataatttcttgggtagtttgcagtttt
aaaattatgttttaaaatggactatcatatgcttaccgtaacttgaaagtatttcgatttcttggctttatatatcttG
TGGAAAGGACGAAACACCGGTCTGCAATATAAGCTGCCAACCACTTGTCA
ATGAATGTGAGGGACTCCTGCTGGAGCAGCCCCCGATTTGTGGGGTGAT
TACAGCTTTTTTTccgttacataacttacggtaaatggcccgcctggctgaccgcccaacgacccccc
gcccattgacgtcaatagtaacgccaatagggactttccattgacgtcaatgggtggagtatttacggtaaac
tgcccacttggcagtacatcaagtgtatcatatgccaagtacgccccctattgacgtcaatgacggtaaatgg
cccgcctggcattGtgcccagtacatgaccttatgggactttcctacttggcagtacatctacgtattagtcatc
gctattaccatggtcgaggtgagccccacgttctgcttcactctccccatctcccccccctccccacccccaat
tttgtatttatttattttttaattattttgtgcagcgatgggggcggggggggggggggggcgcgcgccaggcgg
ggcggggcggggcgaggggcggggcggggcgaggcggagaggtgcggcggcagccaatcagagc
ggcgcgctccgaaagtttccttttatggcgaggcggcggcggcggcggccctataaaaagcgaagcgcg
cggcgggcgggagtcgctgcgacgctgccttcgccccgtgccccgctccgccgccgcctcgcgccgccc
gccccggctctgactgaccgcgttactcccacaggtgagcgggcgggacggcccttctcctccgggctgta
attagctgagcaagaggtaagggtttaagggatggttggttggtggggtattaatgtttaattacctggagcac
ctgcctgaaatcactttttttcaggttGGaccggtgccaccATGaagcggactgctgatggcagtgaatttg
agtccccaaagaagaagagaaaggtgCTGAGCATGTACTGCCTGAAGGACAGCAGA
TTCACCAAGGCCTGGGATAAGCGGGTGCTGCTGTTCAGAGACATCCTGG
CCCAGCTGGGAAGAATCCCCGCCGAGGCCTACGAGTACTACCACGGCG
AGCAGGGTGATAAGAAGAGAGCTAACGACAATGAGGGCACAAATCCCAA
GCGGCACAAGGACAAGTTCATCGAATTTGCACTGCACTACCTGGAAGCC
CAGCACAGCGAGATCTGCTTCGGCAGACGCCACATCGTGCGGGAAGAG
GCCGGCGCCGGCGATGAGCACAAGAAGCACCGGACCAAGGGAAAGGT

GGTGGTGGACTTCAGCAAGAAGGACGAGGACCAGAGCTACTATATCTCC
AAGAACAACGTGATCGTGCGGATCGACAAGAACGCCGGCCCTAGAAGCT
ACCGGATGGGCCTGAACGAGCTGAAGTACCTCGTGCTGCTGAGCCTGCA
GGGGAAGGGCGACGATGCCATCGCCAAGCTGTACAGATACAGACAGCAC
GTGGAGAACATCCTGGATGTGGTGAAGGTGACCGATAAGGATAACCACG
TGTTCCTGCCCCGCTTCGTGCTGGAGCAGCACGGCATCGGCAGAAAGG
CCTTCAAGCAGCGGATCGATGGACGGGTGAAGCACGTGCGGGGCGTGT
GGGAGAAGAAGAAGGCCGCCACCAATGAAATGACCCTGCACGAGAAGG
CCAGAGACATCCTGCAGTACGTGAACGAAAACTGCACCCGGTCCTTCAA
CCCTGGCGAATACAACAGACTGCTGGTGTGCCTGGTGGGCAAGGACGT
GGAGAACTTTCAGGCCGGCCTGAAGCGGCTGCAGCTGGCCGAAAGGAT
CGATGGCCGGGTGTACTCCATCTTCGCCCAGACCAGCACCATCAATGAG
ATGCACCAGGTGGTGTGCGACCAGATCCTGAACCGGCTGTGCAGAATCG
GCGACCAGAAGCTGTACGATTACGTGGGACTGGGCAAGAAGGACGAAAT
CGACTACAAGCAGAAGGTGGCCTGGTTCAAGGAGCACATCAGCATCCGG
AGAGGATTCCTGAGAAAGAAGTTCTGGTACGATAGCAAGAAGGGATTCGC
AAAGCTGGTGGAGGAACACCTGGAGTCCGGCGGCGGCCAGCGCGACG
TGGGCCTGGACAAGAAGTACTACCACATCGACGCCATCGGCAGATTCGA
GGGCGCCAACCCCGCCCTGTACGAGACCCTGGCCAGAGATCGGCTGTG
CCTCATGATGGCCCAGTACTTCCTGGGCAGCGTGAGAAAGGAACTGGGC
AACAAGATTGTGTGGAGCAACGACAGCATCGAACTGCCTaagcgcaccgccga
tggttccgagttcgaaagccccaaaaaaaagcgcaaggtcGGTGGAGGCGGAGGTTCTGG
GGGAGGAGGTAGTGGCGGTGGTGGTTCAGGAGGCGGCGGAAGCcagctg
catttaccgcaggttttagctgacgctgtctcacgcctggtcctgggtaagtttggtgacctgaccgacaacttc
tcctcccctcacgctcgcagaaaagtgctggctggagtcgtcatgacaacaggcacagatgttaaagatgc
caaggtgataagtgtttctacaggaACcaaatgtattaatggtgaatacatgagtgatcgtggccttgcatta
aatgactgccatgcagaaataatatctcggagatccttgctcagatttctttatacacaacttgagctttacttaa
ataacaaagatgatcaaaaaagatccatctttcagaaatcagagcgagggggggtttaggctgaaggaga
atgtccagtttcatctgtacatcagcacctctccctgtggagatgccagaatcttctcaccacatgagccaatc
ctggaagaaccagcagatagacacccaaatcgtaaagcaagaggacagctacggaccaaaatagagt
ctggtcaggggacgattccagtgcgctccaatgcgagcatccaaacgtgggacggggtgctgcaagggg
agcggctgctcaccatgtcctgcagtgacaagattgcacgctggaacgtggtgggcatccagggatcactg
ctcagcattttcgtggagcccatttacttctcgagcatcatcctgggcagcctttaccacggggaccacctttcc
agggccatgtaccagcggatctccaacatagaggacctgccacctctctacaccctcaacaagcctttgct
cagtggcatcagcaatgcagaagcacggcagccagggaaggcccccaacttcagtgtcaactggacgg
taggcgactccgctattgaggtcatcaacgccacgactgggaaggatgagctgggccgcgcgtcccgcct
gtgtaagcACGCGTTGTACTGTCGCTGGATGCGTGTGCACGGCAAGGTTCC
CTCCCACTTACTACGCTCCAAGATTACCAAGCCCAACGTGTACCATGAGT
CCAAGCTGGCGGCAAAGGAGTACCAGGCCGCCAAGGCGCGTCTGTTCA
CAGCCTTCATCAAGGCGGGGCTGGGGGCCTGGGTGGAGAAGCCCACCG
AGCAGGACCAGTTCTCACTCACGAAAAGGCCGGCGGCCACGAAAAAGG
CCGGCCAGGCAAAAAAGAAAAAGtacccatacgatgttccagattacgcttatccctacgac
gtgcctgattatgcatacccatatgatgtccccgactatgccGCGGCCGCTGATTACAAAGAC
GATGACGATAAGtaaATCGAATTCCGCTCGAGATAATCAACCTCTGGATTAC

AAAATTTGTGAAAGATTGACTGGTATTCTTAACTATGTTGCTCCTTTTACGC
TATGTGGATACGCTGCTTTAATGCCTTTGTATCATGCTATTGCTTCCCGTAT
GGCTTTCATTTTCTCCTCCTTGTATAAATCCTGGTTAGTTCTTGCCACGGC
GGAACTCATCGCCGCCTGCCTTGCCCGCTGCTGGACAGGGGCTCGGCT
GTTGGGCACTGACAATTCCGTGGTGTTTATTTGTGAAATTTGTGATGCTAT
TGCTTTATTTGTAACCATCTAGCTTTATTTGTGAAATTTGTGATGCTATTGCT
TTATTTGTAACCATTATAAGCTGCAATAAACAAGTTAACAACAACAATTGCA
TTCATTTTATGTTTCAGGTTCAGGGGGAGATGTGGGAGGTTTTTTAAAGC
GGCCGCCggccgagGGCCGCAGGAACCCCTAGTGATGGAGTTGGCCACTC
CCTCTCTGCGCGCTCGCTCGCTCACTGAGGCCGGGCGACCAAAGGTCG
CCCGACGCCCGGGCTTTGCCCGGGCGGCCTCAGTGAGCGAGCGAGCG
CGCAGCTGCCTGCAGGGGCGCCTGATGCGGTATTTTCTCCTTACGCATC
TGTGCGGTATTTCACACCGCATACGTCAAAGCAACCATAGTACGCGCCCT
GTAGCGGCGCATTAAGCGCGGCGGGTGTGGTGGTTACGCGCAGCGTGA
CCGCTACACTTGCCAGCGCCTTAGCGCCCGCTCCTTTCGCTTTCTTCCCT
TCCTTTCTCGCCACGTTCGCCGGCTTTCCCCGTCAAGCTCTAAATCGGG
GGCTCCCTTTAGGGTTCCGATTTAGTGCTTTACGGCACCTCGACCCCAAA
AAACTTGATTTGGGTGATGGTTCACGTAGTGGGCCATCGCCCTGATAGAC
GGTTTTTCGCCCTTTGACGTTGGAGTCCACGTTCTTTAATAGTGGACTCTT
GTTCCAAACTGGAACAACACTCAACTCTATCTCGGGCTATTCTTTTGATTT
ATAAGGGATTTTGCCGATTTCGGTCTATTGGTTAAAAAATGAGCTGATTTAA
CAAAAATTTAACGCGAATTTTAACAAAATATTAACGTTTACAATTTTATGGTG
CACTCTCAGTACAATCTGCTCTGATGCCGCATAGTTAAGCCAGCCCCGAC
ACCCGCCAACACCCGCTGACGCGCCCTGACGGGCTTGTCTGCTCCCGG
CATCCGCTTACAGACAAGCTGTGACCGTCTccgggagctgcatgtgtcagaggttttca
ccgtcatcaccgaaacgcgcgagACGAAAGGGCCTCGTGATACGCCTATTTTTATAG
GTTAATGTCATGATAATAATGGTTTCTTAGACGTCAGGTGGCACTTTTCGG
GGAAATGTGCGCGGAACCCCTATTTGTTTATTTTTCTAAATACATTCAAATA
TGTATCCGCTCATGAGACAATAACCCTGATAAATGCTTCAATAATATTGAAA
AAGGAAGAGTATGAGTATTCAACATTTCCGTGTCGCCCTTATTCCCTTTTT
TGCGGCATTTTGCCTTCCTGTTTTTGCTCACCCAGAAACGCTGGTGAAAG
TAAAAGATGCTGAAGATCAGTTGGGTGCACGAGTGGGTTACATCGAACTG
GATCTCAACAGCGGTAAGATCCTTGAGAGTTTTCGCCCCGAAGAACGTTT
TCCAATGATGAGCACTTTTAAAGTTCTGCTATGTGGCGCGGTATTATCCCG
TATTGACGCCGGGCAAGAGCAACTCGGTCGCCGCATACACTATTCTCAGA
ATGACTTGGTTGAGTACTCACCAGTCACAGAAAAGCATCTTACGGATGGC
ATGACAGTAAGAGAATTATGCAGTGCTGCCATAACCATGAGTGATAACACT
GCGGCCAACTTACTTCTGACAACGATCGGAGGACCGAAGGAGCTAACCG
CTTTTTTGCACAACATGGGGGATCATGTAACTCGCCTTGATCGTTGGGAA
CCGGAGCTGAATGAAGCCATACCAAACGACGAGCGTGACACCACGATGC
CTGTAGCAATGGCAACAACGTTGCGCAAACTATTAACTGGCGAACTACTTA
CTCTAGCTTCCCGGCAACAATTAATAGACTGGATGGAGGCGGATAAAGTT
GCAGGACCACTTCTGCGCTCGGCCCTTCCGGCTGGCTGGTTTATTGCTG
ATAAATCTGGAGCCGGTGAGCGTGGAAGCCGCGGTATCATTGCAGCACT

GGGGCCAGATGGTAAGCCCTCCCGTATCGTAGTTATCTACACGACGGGG
AGTCAGGCAACTATGGATGAACGAAATAGACAGATCGCTGAGATAGGTGC
CTCACTGATTAAGCATTGGTAACTGTCAGACCAAGTTTACTCATATATACTT
TAGATTGATTTAAAACTTCATTTTTAATTTAAAAGGATCTAGGTGAAGATCCT
TTTTGATAATCTCATGACCAAAATCCCTTAACGTGAGTTTTCGTTCCACTG
AGCGTCAGACCCCGTAGAAAAGATCAAAGGATCTTCTTGAGATCCTTTTTT
TCTGCGCGTAATCTGCTGCTTGCAAACAAAAAAACCACCGCTACCAGCG
GTGGTTTGTTTGCCGGATCAAGAGCTACCAACTCTTTTTCCGAAGGTAAC
TGGCTTCAGCAGAGCGCAGATACCAAATACTGTTCTTCTAGTGTAGCCGT
AGTTAGGCCACCACTTCAAGAACTCTGTAGCACCGCCTACATACCTCGCT
CTGCTAATCCTGTTACCAGTGGCTGCTGCCAGTGGCGATAAGTCGTGTCT
TACCGGGTTGGACTCAAGACGATAGTTACCGGATAAGGCGCAGCGGTCG
GGCTGAACGGGGGGTTCGTGCACACAGCCCAGCTTGGAGCGAACGACC
TACACCGAACTGAGATACCTACAGCGTGAGCTATGAGAAAGCGCCACGCT
TCCCGAAGGGAGAAAGGCGGACAGGTATCCGGTAAGCGGCAGGGTCGG
AACAGGAGAGCGCACGAGGGAGCTTCCAGGGGGAAACGCCTGGTATCT
TTATAGTCCTGTCGGGTTTCGCCACCTCTGACTTGAGCGTCGATTTTTGT
GATGCTCGTCAGGGGGGCGGAGCCTATGGAAAAACGCCAGCAACGCGG
CCTTTTTACGGTTCCTGGCCTTTTGCTGGCCTTTTGCTCACATGT

>E1
CCTGCAGGCAGCTGCGCGCTCGCTCGCTCACTGAGGCCGCCCGGGCG
TCGGGCGACCTTTGGTCGCCCGGCCTCAGTGAGCGAGCGAGCGCGCA
GAGAGGGAGTGGCCAACTCCATCACTAGGGGTTCCTGCGGCCTCTAGAg
agggcctatttcccatgattccttcatatttgcatatacgatacaaggctgttagagagataattggaattaattt
gactgtaaacacaaagatattagtacaaaatacgtgacgtagaaagtaataatttcttgggtagtttgcagtttt
aaaattatgttttaaaatggactatcatatgcttaccgtaacttgaaagtatttcgatttcttggctttatatatcttG
TGGAAAGGACGAAACACCGGTCTGCAATATAAGCTGCCAACCACTTGTCA
ATGAATGTGAGGGACTCCTGCTGGAGCAGCCCCCGATTTGTGGGGTGAT
TACAGCTTTTTTTGATATCtaggtcttgaaaggagtgggaattggctccggtgcccgtcagtggg
cagagcgcacatcgcccacagtccccgagaagttggggggaggggtcggcaattgatccggtgcctaga
gaaggtggcgcggggtaaactgggaaagtgatgtcgtgtactggctccgcctttttcccgagggtgggggga
gaaccgtatataagtgcagtagtcgccgtgaacgttctttttcgcaacgggtttgccgccagaacacaggac
ACCGGTGCCACCATGGCCCCCAAGAAGAAGaGGAAGGTGCTGAGCATGT
ACTGCCTGAAGGACAGCAGATTCACCAAGGCCTGGGATAAGCGGGTGCT
GCTGTTCAGAGACATCCTGGCCCAGCTGGGAAGAATCCCCGCCGAGGC
CTACGAGTACTACCACGGCGAGCAGGGTGATAAGAAGAGAGCTAACGAC
AATGAGGGCACAAATCCCAAGCGGCACAAGGACAAGTTCATCGAATTTGC
ACTGCACTACCTGGAAGCCCAGCACAGCGAGATCTGCTTCGGCAGACGC
CACATCGTGCGGGAAGAGGCCGGCGCCGGCGATGAGCACAAGAAGCAC
CGGACCAAGGGAAAGGTGGTGGTGGACTTCAGCAAGAAGGACGAGGAC
CAGAGCTACTATATCTCCAAGAACAACGTGATCGTGCGGATCGACAAGAA
CGCCGGCCCTAGAAGCTACCGGATGGGCCTGAACGAGCTGAAGTACCTC
GTGCTGCTGAGCCTGCAGGGGAAGGGCGACGATGCCATCGCCAAGCTG

TACAGATACAGACAGCACGTGGAGAACATCCTGGATGTGGTGAAGGTGA
CCGATAAGGATAACCACGTGTTCCTGCCCCGCTTCGTGCTGGAGCAGCA
CGGCATCGGCAGAAAGGCCTTCAAGCAGCGGATCGATGGACGGGTGAA
GCACGTGCGGGGCGTGTGGGAGAAGAAGGCCGCCACCAATGAAAT
GACCCTGCACGAGAAGGCCAGAGACATCCTGCAGTACGTGAACGAAAAC
TGCACCCGGTCCTTCAACCCTGGCGAATACAACAGACTGCTGGTGTGCC
TGGTGGGCAAGGACGTGGAGAACTTTCAGGCCGGCCTGAAGCGGCTGC
AGCTGGCCGAAAGGATCGATGGCCGGGTGTACTCCATCTTCGCCCAGAC
CAGCACCATCAATGAGATGCACCAGGTGGTGTGCGACCAGATCCTGAAC
CGGCTGTGCAGAATCGGCGACCAGAAGCTGTACGATTACGTGGGACTGG
GCAAGAAGGACGAAATCGACTACAAGCAGAAGGTGGCCTGGTTCAAGGA
GCACATCAGCATCCGGAGAGGATTCCTGAGAAAGAAGTTCTGGTACGATA
GCAAGAAGGGATTCGCAAAGCTGGTGGAGGAACACCTGGAGTCCGGCG
GCGGCCAGCGCGACGTGGGCCTGGACAAGAAGTACTACCACATCGACG
CCATCGGCAGATTCGAGGGCGCCAACCCCGCCCTGTACGAGACCCTGG
CCAGAGATCGGCTGTGCCTCATGATGGCCCAGTACTTCCTGGGCAGCGT
GAGAAAGGAACTGGGCAACAAGATTGTGTGGAGCAACGACAGCATCGAA
CTGCCTCCCAAGAAGAAGCGGAAGGTGGGTGGAGGCGGAGGTTCTGGG
GGAGGAGGTAGTGGCGGTGGTGGTTCAGGAGGCGGCGGAAGCcagctgca
tttaccgcaggttttagctgacgctgtctcacgcctggtcctgggtaagtttggtgacctgaccgacaacttctc
ctcccctcacgctcgcagaaaagtgctggctggagtcgtcatgacaacaggcacagatgttaaagatgcc
aaggtgataagtgtttctacaggaACcaaatgtattaatggtgaatacatgagtgatcgtggccttgcattaa
atgactgccatgcagaaataatatctcggagatccttgctcagatttctttatacacaacttgagctttacttaaa
taacaaagatgatcaaaaaagatccatctttcagaaatcagagcgagggggtttaggctgaaggagaat
gtccagtttcatctgtacatcagcacctctccctgtggagatgccagaatcttctcaccacatgagccaatcct
ggaagaaccagcagatagacacccaaatcgtaaagcaagaggacagctacggaccaaaatagagtct
ggtcaggggacgattccagtgcgctccaatgcgagcatccaaacgtgggacggggtgctgcaagggga
gcggctgctcaccatgtcctgcagtgacaagattgcacgctggaacgtggtgggcatccagggatcactgc
tcagcattttcgtggagcccatttacttctcgagcatcatcctgggcagcctttaccacggggaccacctttcca
gggccatgtaccagcggatctccaacatagaggacctgccacctctctacaccctcaacaagcctttgctc
agtggcatcagcaatgcagaagcacggcagccagggaaggcccccaacttcagtgtcaactggacggt
aggcgactccgctattgaggtcatcaacgccacgactgggaaggatgagctgggccgcgcgtcccgcct
gtgtaagcACGCGTTGTACTGTCGCTGGATGCGTGTGCACGGCAAGGTTCC
CTCCCACTTACTACGCTCCAAGATTACCAAGCCCAACGTGTACCATGAGT
CCAAGCTGGCGGCAAAGGAGTACCAGGCCGCCAAGGCGCGTCTGTTCA
CAGCCTTCATCAAGGCGGGGCTGGGGGCCTGGGTGGAGAAGCCCACCG
AGCAGGACCAGTTCTCACTCACGGGATCCtacccatacgatgttccagattacgcttatc
cctacgacgtgcctgattatgcatacccatatgatgtccccgactatgccGCGGCCGCTGATTACA
AAGACGATGACGATAAGtGAgaattcCTAGAGCTCGCTGATCAGCCTCGACT
GTGCCTTCTAGTTGCCAGCCATCTGTTGTTTGCCCCTCCCCCGTGCCTTC
CTTGACCCTGGAAGGTGCCACTCCCACTGTCCTTTCCTAATAAAATGAGG
AAATTGCATCGCATTGTCTGAGTAGGTGTCATTCTATTCTGGGGGGTGGG
GTGGGGCAGGACAGCAAGGGGGAGGATTGGGAAGAgAATAGCAGGCAT
GCTGGGGAgcGGCCGCAGGAACCCCTAGTGATGGAGTTGGCCACTCCCT

CTCTGCGCGCTCGCTCGCTCACTGAGGCCGGGCGACCAAAGGTCGCCC
GACGCCCGGGCTTTGCCCGGGCGGCCTCAGTGAGCGAGCGAGCGCGC
AGCTGCCTGCAGGGGCGCCTGATGCGGTATTTTCTCCTTACGCATCTGTG
CGGTATTTCACACCGCATACGTCAAAGCAACCATAGTACGCGCCCTGTAG
CGGCGCATTAAGCGCGGCGGGTGTGGTGGTTACGCGCAGCGTGACCGC
TACACTTGCCAGCGCCTTAGCGCCCGCTCCTTTCGCTTTCTTCCCTTCCT
TTCTCGCCACGTTCGCCGGCTTTCCCCGTCAAGCTCTAAATCGGGGGCT
CCCTTTAGGGTTCCGATTTAGTGCTTTACGGCACCTCGACCCCAAAAAAC
TTGATTTGGGTGATGGTTCACGTAGTGGGCCATCGCCCTGATAGACGGTT
TTTCGCCCTTTGACGTTGGAGTCCACGTTCTTTAATAGTGGACTCTTGTTC
CAAACTGGAACAACACTCAACTCTATCTCGGGCTATTCTTTTGATTTATAAG
GGATTTTGCCGATTTCGGTCTATTGGTTAAAAAATGAGCTGATTTAACAAA
AATTTAACGCGAATTTTAACAAAATATTAACGTTTACAATTTTATGGTGCACT
CTCAGTACAATCTGCTCTGATGCCGCATAGTTAAGCCAGCCCCGACACCC
GCCAACACCCGCTGACGCGCCCTGACGGGCTTGTCTGCTCCCGGCATC
CGCTTACAGACAAGCTGTGACCGTCTccgggagctgcatgtgtcagaggttttcaccgtca
tcaccgaaacgcgcgagACGAAAGGGCCTCGTGATACGCCTATTTTTATAGGTTA
ATGTCATGATAATAATGGTTTCTTAGACGTCAGGTGGCACTTTTCGGGGAA
ATGTGCGCGGAACCCCTATTTGTTTATTTTTCTAAATACATTCAAATATGTAT
CCGCTCATGAGACAATAACCCTGATAAATGCTTCAATAATATTGAAAAAGG
AAGAGTATGAGTATTCAACATTTCCGTGTCGCCCTTATTCCCTTTTTTGCG
GCATTTTGCCTTCCTGTTTTTGCTCACCCAGAAACGCTGGTGAAAGTAAA
AGATGCTGAAGATCAGTTGGGTGCACGAGTGGGTTACATCGAACTGGAT
CTCAACAGCGGTAAGATCCTTGAGAGTTTTCGCCCCGAAGAACGTTTTCC
AATGATGAGCACTTTTAAAGTTCTGCTATGTGGCGCGGTATTATCCCGTAT
TGACGCCGGGCAAGAGCAACTCGGTCGCCGCATACACTATTCTCAGAAT
GACTTGGTTGAGTACTCACCAGTCACAGAAAAGCATCTTACGGATGGCAT
GACAGTAAGAGAATTATGCAGTGCTGCCATAACCATGAGTGATAACACTG
CGGCCAACTTACTTCTGACAACGATCGGAGGACCGAAGGAGCTAACCGC
TTTTTTGCACAACATGGGGGATCATGTAACTCGCCTTGATCGTTGGGAAC
CGGAGCTGAATGAAGCCATACCAAACGACGAGCGTGACACCACGATGCC
TGTAGCAATGGCAACAACGTTGCGCAAACTATTAACTGGCGAACTACTTAC
TCTAGCTTCCCGGCAACAATTAATAGACTGGATGGAGGCGGATAAAGTTG
CAGGACCACTTCTGCGCTCGGCCCTTCCGGCTGGCTGGTTTATTGCTGA
TAAATCTGGAGCCGGTGAGCGTGGAAGCCGCGGTATCATTGCAGCACTG
GGGCCAGATGGTAAGCCCTCCCGTATCGTAGTTATCTACACGACGGGGA
GTCAGGCAACTATGGATGAACGAAATAGACAGATCGCTGAGATAGGTGCC
TCACTGATTAAGCATTGGTAACTGTCAGACCAAGTTTACTCATATATACTTT
AGATTGATTTAAAACTTCATTTTTAATTTAAAAGGATCTAGGTGAAGATCCTT
TTTGATAATCTCATGACCAAAATCCCTTAACGTGAGTTTTCGTTCCACTGA
GCGTCAGACCCCGTAGAAAAGATCAAAGGATCTTCTTGAGATCCTTTTTTT
CTGCGCGTAATCTGCTGCTTGCAAACAAAAAAACCACCGCTACCAGCGG
TGGTTTGTTTGCCGGATCAAGAGCTACCAACTCTTTTTCCGAAGGTAACT
GGCTTCAGCAGAGCGCAGATACCAAATACTGTTCTTCTAGTGTAGCCGTA

GTTAGGCCACCACTTCAAGAACTCTGTAGCACCGCCTACATACCTCGCTC
TGCTAATCCTGTTACCAGTGGCTGCTGCCAGTGGCGATAAGTCGTGTCTT
ACCGGGTTGGACTCAAGACGATAGTTACCGGATAAGGCGCAGCGGTCGG
GCTGAACGGGGGGTTCGTGCACACAGCCCAGCTTGGAGCGAACGACCT
ACACCGAACTGAGATACCTACAGCGTGAGCTATGAGAAAGCGCCACGCT
TCCCGAAGGGAGAAAGGCGGACAGGTATCCGGTAAGCGGCAGGGTCGG
AACAGGAGAGCGCACGAGGGAGCTTCCAGGGGGAAACGCCTGGTATCT
TTATAGTCCTGTCGGGTTTCGCCACCTCTGACTTGAGCGTCGATTTTTGT
GATGCTCGTCAGGGGGGCGGAGCCTATGGAAAAACGCCAGCAACGCGG
CCTTTTTACGGTTCCTGGCCTTTTGCTGGCCTTTTGCTCACATGT

>E2
CCTGCAGGCAGCTGCGCGCTCGCTCGCTCACTGAGGCCGCCCGGGCG
TCGGGCGACCTTTGGTCGCCCGGCCTCAGTGAGCGAGCGAGCGCGCA
GAGAGGGAGTGGCCAACTCCATCACTAGGGGTTCCTGCGGCCTCTAGAg
agggcctatttcccatgattccttcatatttgcatatacgatacaaggctgttagagagataattggaattaattt
gactgtaaacacaaagatattagtacaaaatacgtgacgtagaaagtaataatttcttgggtagtttgcagtttt
aaaattatgttttaaaatggactatcatatgcttaccgtaacttgaaagtatttcgatttcttggctttatatatcttG
TGGAAAGGACGAAACACCGGCTGGAGCAGCCCCCGATTTGTGGGGTGA
TTACAGCGTCTGCAATATAAGCTGCCAACCACTTGTCAATGAATGTGAGG
GACTCCTGCTGGAGCAGCCCCCGATTTGTGGGGTGATTACAGCTTTTTTT
GATATCtaggtcttgaaaggagtgggaattggctccggtgcccgtcagtgggcagagcgcacatcgcc
cacagtccccgagaagttggggggaggggtcggcaattgatccggtgcctagagaaggtggcgcggggt
aaactgggaaagtgatgtcgtgtactggctccgcctttttcccgagggtgggggagaaccgtatataagtgc
agtagtcgccgtgaacgttctttttcgcaacgggtttgccgccagaacacaggacACCGGTGCCAC
CATGGCCCCCAAGAAGAAGaGGAAGGTGCTGAGCATGTACTGCCTGAAG
GACAGCAGATTCACCAAGGCCTGGGATAAGCGGGTGCTGCTGTTCAGAG
ACATCCTGGCCCAGCTGGGAAGAATCCCCGCCGAGGCCTACGAGTACTA
CCACGGCGAGCAGGGTGATAAGAAGAGAGCTAACGACAATGAGGGCACA
AATCCCAAGCGGCACAAGGACAAGTTCATCGAATTTGCACTGCACTACCT
GGAAGCCCAGCACAGCGAGATCTGCTTCGGCAGACGCCACATCGTGCG
GGAAGAGGCCGGCGCCGGCGATGAGCACAAGAAGCACCGGACCAAGG
GAAAGGTGGTGGTGGACTTCAGCAAGAAGGACGAGGACCAGAGCTACTA
TATCTCCAAGAACAACGTGATCGTGCGGATCGACAAGAACGCCGGCCCT
AGAAGCTACCGGATGGGCCTGAACGAGCTGAAGTACCTCGTGCTGCTGA
GCCTGCAGGGGAAGGGCGACGATGCCATCGCCAAGCTGTACAGATACAG
ACAGCACGTGGAGAACATCCTGGATGTGGTGAAGGTGACCGATAAGGAT
AACCACGTGTTCCTGCCCCGCTTCGTGCTGGAGCAGCACGGCATCGGC
AGAAAGGCCTTCAAGCAGCGGATCGATGGACGGGTGAAGCACGTGCGG
GGCGTGTGGGAGAAGAAGAAGGCCGCCACCAATGAAATGACCCTGCAC
GAGAAGGCCAGAGACATCCTGCAGTACGTGAACGAAAACTGCACCCGGT
CCTTCAACCCTGGCGAATACAACAGACTGCTGGTGTGCCTGGTGGGCAA
GGACGTGGAGAACTTTCAGGCCGGCCTGAAGCGGCTGCAGCTGGCCGA
AAGGATCGATGGCCGGGTGTACTCCATCTTCGCCCAGACCAGCACCATC

AATGAGATGCACCAGGTGGTGTGCGACCAGATCCTGAACCGGCTGTGCA
GAATCGGCGACCAGAAGCTGTACGATTACGTGGGACTGGGCAAGAAGGA
CGAAATCGACTACAAGCAGAAGGTGGCCTGGTTCAAGGAGCACATCAGC
ATCCGGAGAGGATTCCTGAGAAAGAAGTTCTGGTACGATAGCAAGAAGG
GATTCGCAAAGCTGGTGGAGGAACACCTGGAGTCCGGCGGCGGCCAGC
GCGACGTGGGCCTGGACAAGAAGTACTACCACATCGACGCCATCGGCAG
ATTCGAGGGCGCCAACCCCGCCCTGTACGAGACCCTGGCCAGAGATCG
GCTGTGCCTCATGATGGCCCAGTACTTCCTGGGCAGCGTGAGAAAGGAA
CTGGGCAACAAGATTGTGTGGAGCAACGACAGCATCGAACTGCCTCCCA
AGAAGAAGCGGAAGGTGGGTGGAGGCGGAGGTTCTGGGGGAGGAGGT
AGTGGCGGTGGTGGTTCAGGAGGCGGCGGAAGCcagctgcatttaccgcaggtttt
agctgacgctgtctcacgcctggtcctgggtaagtttggtgacctgaccgacaacttctcctcccctcacgctc
gcagaaaagtgctggctggagtcgtcatgacaacaggcacagatgttaaagatgccaaggtgataagtgt
ttctacaggaACcaaatgtattaatggtgaatacatgagtgatcgtggccttgcattaaatgactgccatgca
gaaataatatctcggagatccttgctcagatttctttatacacaacttgagctttacttaaataacaaagatgatc
aaaaaagatccatctttcagaaatcagagcgagggggtttaggctgaaggagaatgtccagtttcatctgt
acatcagcacctctccctgtggagatgccagaatcttctcaccacatgagccaatcctggaagaaccagca
gatagacacccaaatcgtaaagcaagaggacagctacggaccaaaatagagtctggtcaggggacgat
tccagtgcgctccaatgcgagcatccaaacgtgggacggggtgctgcaaggggagcggctgctcaccat
gtcctgcagtgacaagattgcacgctggaacgtggtgggcatccagggatcactgctcagcattttcgtgga
gcccatttacttctcgagcatcatcctgggcagcctttaccacggggaccacctttccagggccatgtaccag
cggatctccaacatagaggacctgccacctctctacaccctcaacaagcctttgctcagtggcatcagcaat
gcagaagcacggcagccagggaaggcccccaacttcagtgtcaactggacggtaggcgactccgctatt
gaggtcatcaacgccacgactgggaaggatgagctgggccgcgcgtcccgcctgtgtaagcACGCG
TTGTACTGTCGCTGGATGCGTGTGCACGGCAAGGTTCCCTCCCACTTACT
ACGCTCCAAGATTACCAAGCCCAACGTGTACCATGAGTCCAAGCTGGCG
GCAAAGGAGTACCAGGCCGCCAAGGCGCGTCTGTTCACAGCCTTCATCA
AGGCGGGGCTGGGGGCCTGGGTGGAGAAGCCCACCGAGCAGGACCAG
TTCTCACTCACGGGATCCtacccatacgatgttccagattacgcttatccctacgacgtgcctgatt
atgcatacccatatgatgtccccgactatgccGCGGCCGCTGATTACAAAGACGATGACG
ATAAGtGAgaattcCTAGAGCTCGCTGATCAGCCTCGACTGTGCCTTCTAGTT
GCCAGCCATCTGTTGTTTGCCCCTCCCCCGTGCCTTCCTTGACCCTGGA
AGGTGCCACTCCCACTGTCCTTTCCTAATAAAATGAGGAAATTGCATCGCA
TTGTCTGAGTAGGTGTCATTCTATTCTGGGGGGTGGGGTGGGGCAGGAC
AGCAAGGGGGAGGATTGGGAAGAgAATAGCAGGCATGCTGGGGAgcGGC
CGCAGGAACCCCTAGTGATGGAGTTGGCCACTCCCTCTCTGCGCGCTCG
CTCGCTCACTGAGGCCGGGCGACCAAAGGTCGCCCGACGCCCGGGCTT
TGCCCGGGCGGCCTCAGTGAGCGAGCGAGCGCGCAGCTGCCTGCAGG
GGCGCCTGATGCGGTATTTTCTCCTTACGCATCTGTGCGGTATTTCACAC
CGCATACGTCAAAGCAACCATAGTACGCGCCCTGTAGCGGCGCATTAAGC
GCGGCGGGTGTGGTGGTTACGCGCAGCGTGACCGCTACACTTGCCAGC
GCCTTAGCGCCCGCTCCTTTCGCTTTCTTCCCTTCCTTTCTCGCCACGTT
CGCCGGCTTTCCCCGTCAAGCTCTAAATCGGGGGCTCCCTTTAGGGTTC
CGATTTAGTGCTTTACGGCACCTCGACCCCAAAAAACTTGATTTGGGTGA

3050 TGGTTCACGTAGTGGGCCATCGCCCTGATAGACGGTTTTTCGCCCTTTGA
3051 CGTTGGAGTCCACGTTCTTTAATAGTGGACTCTTGTTCCAAACTGGAACA
3052 ACACTCAACTCTATCTCGGGCTATTCTTTTGATTTATAAGGGATTTTGCCGA
3053 TTTCGGTCTATTGGTTAAAAAATGAGCTGATTTAACAAAAATTTAACGCGAA
3054 TTTTAACAAAATATTAACGTTTACAATTTTATGGTGCACTCTCAGTACAATCT
3055 GCTCTGATGCCGCATAGTTAAGCCAGCCCCGACACCCGCCAACACCCGC
3056 TGACGCGCCCTGACGGGCTTGTCTGCTCCCGGCATCCGCTTACAGACAA
3057 GCTGTGACCGTCTccgggagctgcatgtgtcagaggttttcaccgtcatcaccgaaacgcgcgag
3058 ACGAAAGGGCCTCGTGATACGCCTATTTTTATAGGTTAATGTCATGATAATA
3059 ATGGTTTCTTAGACGTCAGGTGGCACTTTTCGGGGAAATGTGCGCGGAA
3060 CCCCTATTTGTTTATTTTTCTAAATACATTCAAATATGTATCCGCTCATGAGA
3061 CAATAACCCTGATAAATGCTTCAATAATATTGAAAAAGGAAGAGTATGAGTA
3062 TTCAACATTTCCGTGTCGCCCTTATTCCCTTTTTTGCGGCATTTTGCCTTC
3063 CTGTTTTTGCTCACCCAGAAACGCTGGTGAAAGTAAAAGATGCTGAAGAT
3064 CAGTTGGGTGCACGAGTGGGTTACATCGAACTGGATCTCAACAGCGGTA
3065 AGATCCTTGAGAGTTTTCGCCCCGAAGAACGTTTTCCAATGATGAGCACT
3066 TTTAAAGTTCTGCTATGTGGCGCGGTATTATCCCGTATTGACGCCGGGCA
3067 AGAGCAACTCGGTCGCCGCATACACTATTCTCAGAATGACTTGGTTGAGT
3068 ACTCACCAGTCACAGAAAAGCATCTTACGGATGGCATGACAGTAAGAGAA
3069 TTATGCAGTGCTGCCATAACCATGAGTGATAACACTGCGGCCAACTTACTT
3070 CTGACAACGATCGGAGGACCGAAGGAGCTAACCGCTTTTTTGCACAACA
3071 TGGGGGATCATGTAACTCGCCTTGATCGTTGGGAACCGGAGCTGAATGA
3072 AGCCATACCAAACGACGAGCGTGACACCACGATGCCTGTAGCAATGGCA
3073 ACAACGTTGCGCAAACTATTAACTGGCGAACTACTTACTCTAGCTTCCCG
3074 GCAACAATTAATAGACTGGATGGAGGCGGATAAAGTTGCAGGACCACTTC
3075 TGCGCTCGGCCCTTCCGGCTGGCTGGTTTATTGCTGATAAATCTGGAGCC
3076 GGTGAGCGTGGAAGCCGCGGTATCATTGCAGCACTGGGGCCAGATGGTA
3077 AGCCCTCCCGTATCGTAGTTATCTACACGACGGGGAGTCAGGCAACTATG
3078 GATGAACGAAATAGACAGATCGCTGAGATAGGTGCCTCACTGATTAAGCA
3079 TTGGTAACTGTCAGACCAAGTTTACTCATATATACTTTAGATTGATTTAAAAC
3080 TTCATTTTTAATTTAAAAGGATCTAGGTGAAGATCCTTTTTGATAATCTCATG
3081 ACCAAAATCCCTTAACGTGAGTTTTCGTTCCACTGAGCGTCAGACCCCGT
3082 AGAAAAGATCAAAGGATCTTCTTGAGATCCTTTTTTTCTGCGCGTAATCTG
3083 CTGCTTGCAAACAAAAAAACCACCGCTACCAGCGGTGGTTTGTTTGCCG
3084 GATCAAGAGCTACCAACTCTTTTTCCGAAGGTAACTGGCTTCAGCAGAGC
3085 GCAGATACCAAATACTGTTCTTCTAGTGTAGCCGTAGTTAGGCCACCACTT
3086 CAAGAACTCTGTAGCACCGCCTACATACCTCGCTCTGCTAATCCTGTTACC
3087 AGTGGCTGCTGCCAGTGGCGATAAGTCGTGTCTTACCGGGTTGGACTCA
3088 AGACGATAGTTACCGGATAAGGCGCAGCGGTCGGGCTGAACGGGGGGT
3089 TCGTGCACACAGCCCAGCTTGGAGCGAACGACCTACACCGAACTGAGAT
3090 ACCTACAGCGTGAGCTATGAGAAAGCGCCACGCTTCCCGAAGGGAGAAA
3091 GGCGGACAGGTATCCGGTAAGCGGCAGGGTCGGAACAGGAGAGCGCAC
3092 GAGGGAGCTTCCAGGGGGAAACGCCTGGTATCTTTATAGTCCTGTCGGG
3093 TTTCGCCACCTCTGACTTGAGCGTCGATTTTTGTGATGCTCGTCAGGGGG

GCGGAGCCTATGGAAAAACGCCAGCAACGCGGCCTTTTTACGGTTCCTG
GCCTTTTGCTGGCCTTTTGCTCACATGT

>E3
CCTGCAGGCAGCTGCGCGCTCGCTCGCTCACTGAGGCCGCCCGGGCG
TCGGGCGACCTTTGGTCGCCCGGCCTCAGTGAGCGAGCGAGCGCGCA
GAGAGGGAGTGGCCAACTCCATCACTAGGGGTTCCTGCGGCCTCTAGAg
agggcctatttcccatgattccttcatatttgcatatacgatacaaggctgttagagagataattggaattaattt
gactgtaaacacaaagatattagtacaaaatacgtgacgtagaaagtaataatttcttgggtagtttgcagtttt
aaaattatgttttaaaatggactatcatatgcttaccgtaacttgaaagtatttcgatttcttggctttatatatcttG
TGGAAAGGACGAAACACCGGTCTGCAATATAAGCTGCCAACCACTTGTCA
ATGAATGTGAGGGACTCCTGCTGGAGCAGCCCCCGATTTGTGGGGTGAT
TACAGCTTTTTTTGATATCtaggtcttgaaaggagtgggaattggctccggtgcccgtcagtggg
cagagcgcacatcgcccacagtccccgagaagttggggggaggggtcggcaattgatccggtgcctaga
gaaggtggcgcggggtaaactgggaaagtgatgtcgtgtactggctccgcctttttcccgagggtgggggga
gaaccgtatataagtgcagtagtcgccgtgaacgttctttttcgcaacgggtttgccgccagaacacaggac
ACCGGTGCCACCATGGCCCCCAAGAAGAAGaGGAAGGTGCTGAGCATGT
ACTGCCTGAAGGACAGCAGATTCACCAAGGCCTGGGATAAGCGGGTGCT
GCTGTTCAGAGACATCCTGGCCCAGCTGGGAAGAATCCCCGCCGAGGC
CTACGAGTACTACCACGGCGAGCAGGGTGATAAGAAGAGAGCTAACGAC
AATGAGGGCACAAATCCCAAGCGGCACAAGGACAAGTTCATCGAATTTGC
ACTGCACTACCTGGAAGCCCAGCACAGCGAGATCTGCTTCGGCAGACGC
CACATCGTGCGGGAAGAGGCCGGCGCCGGCGATGAGCACAAGAAGCAC
CGGACCAAGGGAAAGGTGGTGGTGGACTTCAGCAAGAAGGACGAGGAC
CAGAGCTACTATATCTCCAAGAACAACGTGATCGTGCGGATCGACAAGAA
CGCCGGCCCTAGAAGCTACCGGATGGGCCTGAACGAGCTGAAGTACCTC
GTGCTGCTGAGCCTGCAGGGGAAGGGCGACGATGCCATCGCCAAGCTG
TACAGATACAGACAGCACGTGGAGAACATCCTGGATGTGGTGAAGGTGA
CCGATAAGGATAACCACGTGTTCCTGCCCCGCTTCGTGCTGGAGCAGCA
CGGCATCGGCAGAAAGGCCTTCAAGCAGCGGATCGATGGACGGGTGAA
GCACGTGCGGGGCGTGTGGGAGAAGAAGAAGGCCGCCACCAATGAAAT
GACCCTGCACGAGAAGGCCAGAGACATCCTGCAGTACGTGAACGAAAAC
TGCACCCGGTCCTTCAACCCTGGCGAATACAACAGACTGCTGGTGTGCC
TGGTGGGCAAGGACGTGGAGAACTTTCAGGCCGGCCTGAAGCGGCTGC
AGCTGGCCGAAAGGATCGATGGCCGGGTGTACTCCATCTTCGCCCAGAC
CAGCACCATCAATGAGATGCACCAGGTGGTGTGCGACCAGATCCTGAAC
CGGCTGTGCAGAATCGGCGACCAGAAGCTGTACGATTACGTGGGACTGG
GCAAGAAGGACGAAATCGACTACAAGCAGAAGGTGGCCTGGTTCAAGGA
GCACATCAGCATCCGGAGAGGATTCCTGAGAAAGAAGTTCTGGTACGATA
GCAAGAAGGGATTCGCAAAGCTGGTGGAGGAACACCTGGAGTCCGGCG
GCGGCCAGCGCGACGTGGGCCTGGACAAGAAGTACTACCACATCGACG
CCATCGGCAGATTCGAGGGCGCCAACCCCGCCCTGTACGAGACCCTGG
CCAGAGATCGGCTGTGCCTCATGATGGCCCAGTACTTCCTGGGCAGCGT
GAGAAAGGAACTGGGCAACAAGATTGTGTGGAGCAACGACAGCATCGAA

CTGCCTCCCAAGAAGAAGCGGAAGGTGGGTGGAGGCGGAGGTTCTGGG
GGAGGAGGTAGTGGCGGTGGTGGTTCAGGAGGCGGCGGAAGCcagctgca
tttaccgcaggttttagctgacgctgtctcacgcctggtcctgggtaagtttggtgacctgaccgacaacttctc
ctcccctcacgctcgcagaaaagtgctggctggagtcgtcatgacaacaggcacagatgttaaagatgcc
aaggtgataagtgtttctacaggaACcaaatgtattaatggtgaatacatgagtgatcgtggccttgcattaa
atgactgccatgcagaaataatatctcggagatccttgctcagatttctttatacacaacttgagctttacttaaa
taacaaagatgatcaaaaaagatccatctttcagaaatcagagcgaggggggtttaggctgaaggagaat
gtccagtttcatctgtacatcagcacctctccctgtggagatgccagaatcttctcaccacatgagccaatcct
ggaagaaccagcagatagacacccaaatcgtaaagcaagaggacagctacggaccaaaatagagtct
ggtcaggggacgattccagtgcgctccaatgcgagcatccaaacgtgggacggggtgctgcaaggga
gcggctgctcaccatgtcctgcagtgacaagattgcacgctggaacgtggtgggcatccagggatcactgc
tcagcattttcgtggagcccatttacttctcgagcatcatcctgggcagcctttaccacggggaccacctttcca
gggccatgtaccagcggatctccaacatagaggacctgccacctctctacaccctcaacaagcctttgctc
agtggcatcagcaatgcagaagcacggcagccagggaaggcccccaacttcagtgtcaactggacggt
aggcgactccgctattgaggtcatcaacgccacgactgggaaggatgagctgggccgcgcgtcccgcct
gtgtaagcACGCGTTGTACTGTCGCTGGATGCGTGTGCACGGCAAGGTTCC
CTCCCACTTACTACGCTCCAAGATTACCAAGCCCAACGTGTACCATGAGT
CCAAGCTGGCGGCAAAGGAGTACCAGGCCGCCAAGGCGCGTCTGTTCA
CAGCCTTCATCAAGGCGGGGCTGGGGGCCTGGGTGGAGAAGCCCACCG
AGCAGGACCAGTTCTCACTCACGGGATCCtacccatacgatgttccagattacgcttatc
cctacgacgtgcctgattatgcatacccatatgatgtccccgactatgccGCGGCCGCTGATTACA
AAGACGATGACGATAAGtAAATCGAATTCCGCTCGAGATAATCAACCTCTG
GATTACAAAATTTGTGAAAGATTGACTGGTATTCTTAACTATGTTGCTCCTT
TTACGCTATGTGGATACGCTGCTTTAATGCCTTTGTATCATGCTATTGCTTC
CCGTATGGCTTTCATTTTCTCCTCCTTGTATAAATCCTGGTTAGTTCTTGCC
ACGGCGGAACTCATCGCCGCCTGCCTTGCCCGCTGCTGGACAGGGGCT
CGGCTGTTGGGCACTGACAATTCCGTGGTGTTTATTTGTGAAATTTGTGAT
GCTATTGCTTTATTTGTAACCATCTAGCTTTATTTGTGAAATTTGTGATGCTA
TTGCTTTATTTGTAACCATTATAAGCTGCAATAAACAAGTTAACAACAACAA
TTGCATTCATTTTATGTTTCAGGTTCAGGGGGAGATGTGGGAGGTTTTTTA
AAGCGGCCGCCGGCCGAGGGCCGCAGGAACCCCTAGTGATGGAGTTGG
CCACTCCCTCTCTGCGCGCTCGCTCGCTCACTGAGGCCGGGCGACCAA
AGGTCGCCCGACGCCCGGGCTTTGCCCGGGCGGCCTCAGTGAGCGAG
CGAGCGCGCAGCTGCCTGCAGGGGCGCCTGATGCGGTATTTTCTCCTTA
CGCATCTGTGCGGTATTTCACACCGCATACGTCAAAGCAACCATAGTACG
CGCCCTGTAGCGGCGCATTAAGCGCGGCGGGTGTGGTGGTTACGCGCA
GCGTGACCGCTACACTTGCCAGCGCCTTAGCGCCCGCTCCTTTCGCTTT
CTTCCCTTCCTTTCTCGCCACGTTCGCCGGCTTTCCCCGTCAAGCTCTAA
ATCGGGGGCTCCCTTTAGGGTTCCGATTTAGTGCTTTACGGCACCTCGAC
CCCAAAAAACTTGATTTGGGTGATGGTTCACGTAGTGGGCCATCGCCCTG
ATAGACGGTTTTTCGCCCTTTGACGTTGGAGTCCACGTTCTTTAATAGTGG
ACTCTTGTTCCAAACTGGAACAACACTCAACTCTATCTCGGGCTATTCTTT
TGATTTATAAGGGATTTTGCCGATTTCGGTCTATTGGTTAAAAAATGAGCTG
ATTTAACAAAAATTTAACGCGAATTTTAACAAAATATTAACGTTTACAATTTTA

TGGTGCACTCTCAGTACAATCTGCTCTGATGCCGCATAGTTAAGCCAGCC
CCGACACCCGCCAACACCCGCTGACGCGCCCTGACGGGCTTGTCTGCT
CCCGGCATCCGCTTACAGACAAGCTGTGACCGTCTccgggagctgcatgtgtcaga
ggttttcaccgtcatcaccgaaacgcgcgagACGAAAGGGCCTCGTGATACGCCTATTT
TTATAGGTTAATGTCATGATAATAATGGTTTCTTAGACGTCAGGTGGCACTT
TTCGGGGAAATGTGCGCGGAACCCCTATTTGTTTATTTTTCTAAATACATTC
AAATATGTATCCGCTCATGAGACAATAACCCTGATAAATGCTTCAATAATATT
GAAAAAGGAAGAGTATGAGTATTCAACATTTCCGTGTCGCCCTTATTCCCT
TTTTTGCGGCATTTTGCCTTCCTGTTTTTGCTCACCCAGAAACGCTGGTG
AAAGTAAAAGATGCTGAAGATCAGTTGGGTGCACGAGTGGGTTACATCGA
ACTGGATCTCAACAGCGGTAAGATCCTTGAGAGTTTTCGCCCCGAAGAAC
GTTTTCCAATGATGAGCACTTTTAAAGTTCTGCTATGTGGCGCGGTATTAT
CCCGTATTGACGCCGGGCAAGAGCAACTCGGTCGCCGCATACACTATTC
TCAGAATGACTTGGTTGAGTACTCACCAGTCACAGAAAAGCATCTTACGG
ATGGCATGACAGTAAGAGAATTATGCAGTGCTGCCATAACCATGAGTGATA
ACACTGCGGCCAACTTACTTCTGACAACGATCGGAGGACCGAAGGAGCT
AACCGCTTTTTTGCACAACATGGGGGATCATGTAACTCGCCTTGATCGTT
GGGAACCGGAGCTGAATGAAGCCATACCAAACGACGAGCGTGACACCAC
GATGCCTGTAGCAATGGCAACAACGTTGCGCAAACTATTAACTGGCGAAC
TACTTACTCTAGCTTCCCGGCAACAATTAATAGACTGGATGGAGGCGGATA
AAGTTGCAGGACCACTTCTGCGCTCGGCCCTTCCGGCTGGCTGGTTTAT
TGCTGATAAATCTGGAGCCGGTGAGCGTGGAAGCCGCGGTATCATTGCA
GCACTGGGGCCAGATGGTAAGCCCTCCCGTATCGTAGTTATCTACACGAC
GGGGAGTCAGGCAACTATGGATGAACGAAATAGACAGATCGCTGAGATAG
GTGCCTCACTGATTAAGCATTGGTAACTGTCAGACCAAGTTTACTCATATAT
ACTTTAGATTGATTTAAAACTTCATTTTTAATTTAAAAGGATCTAGGTGAAGA
TCCTTTTTGATAATCTCATGACCAAAATCCCTTAACGTGAGTTTTCGTTCCA
CTGAGCGTCAGACCCCGTAGAAAAGATCAAAGGATCTTCTTGAGATCCTT
TTTTTCTGCGCGTAATCTGCTGCTTGCAAACAAAAAAACCACCGCTACCA
GCGGTGGTTTGTTTGCCGGATCAAGAGCTACCAACTCTTTTTCCGAAGGT
AACTGGCTTCAGCAGAGCGCAGATACCAAATACTGTTCTTCTAGTGTAGC
CGTAGTTAGGCCACCACTTCAAGAACTCTGTAGCACCGCCTACATACCTC
GCTCTGCTAATCCTGTTACCAGTGGCTGCTGCCAGTGGCGATAAGTCGTG
TCTTACCGGGTTGGACTCAAGACGATAGTTACCGGATAAGGCGCAGCGG
TCGGGCTGAACGGGGGGTTCGTGCACACAGCCCAGCTTGGAGCGAACG
ACCTACACCGAACTGAGATACCTACAGCGTGAGCTATGAGAAAGCGCCAC
GCTTCCCGAAGGGAGAAAGGCGGACAGGTATCCGGTAAGCGGCAGGGT
CGGAACAGGAGAGCGCACGAGGGAGCTTCCAGGGGGAAACGCCTGGTA
TCTTTATAGTCCTGTCGGGTTTCGCCACCTCTGACTTGAGCGTCGATTTTT
GTGATGCTCGTCAGGGGGGCGGAGCCTATGGAAAAACGCCAGCAACGC
GGCCTTTTTACGGTTCCTGGCCTTTTGCTGGCCTTTTGCTCACATGT

>M1
CCTGCAGGCAGCTGCGCGCTCGCTCGCTCACTGAGGCCGCCCGGGCG

TCGGGCGACCTTTGGTCGCCCGGCCTCAGTGAGCGAGCGAGCGCGCA
GAGAGGGAGTGGCCAACTCCATCACTAGGGGTTCCTGCGGCCTCTAGAg
agggcctatttcccatgattccttcatatttgcatatacgatacaaggctgttagagagataattggaattaattt
gactgtaaacacaaagatattagtacaaaatacgtgacgtagaaagtaataatttcttgggtagtttgcagtttt
aaaattatgttttaaaatggactatcatatgcttaccgtaacttgaaagtatttcgatttcttggctttatatatcttG
TGGAAAGGACGAAACACCGGCTGGAGCAGCCCCCGATTTGTGGGGTGA
TTACAGCGTCTGCAATATAAGCTGCCAACCACTTGTCAATGAATGTGAGG
GACTCCTGCTGGAGCAGCCCCCGATTTGTGGGGTGATTACAGCTTTTTTT
GATATCACCCTTCAGATTAAAAATAACTGAGGTAAGGGCCTGGGTAGGGG
AGGTGGTGTGAGACGCTCCTGTCTCTCCTCTATCTGCCCATCGGCCCTTT
GGGGAGGAGGAATGTGCCCAAGGACTAAAAAAAGGCCATGGAGCCAGA
GGGGCGAGGGCAACAGACCTTTCATGGGCAAACCTTGGGGCCCTGCTG
TCTAGCATGCCCCACTACGGGTCTAGGCTGCCCATGTAAGGAGGCAAGG
CCTGGGGACACCCGAGATGCCTGGTTATAATTAACCCAGACATGTGGCTG
CCCCCCCCCCCCCAACACCTGCTGCCTCTAAAAATAACCCTGTCCCTGG
TGGATCCCCTGCATGCGAAGATCTTCGAACAAGGCTGTGGGGGACTGAG
GGCAGGCTGTAACAGGCTTGGGGGCCAGGGCTTATACGTGCCTGGGAC
TCCCAAAGTATTACTGTTCCATGTTCCCGGCGAAGGGCCAGCTGTCCCCC
GCCAGCTAGACTCAGCACTTAGTTTAGGAACCAGTGAGCAAGTCAGCCC
TTGGGGCAGCCCATACAAGGCCATGGGGCTGGGCAAGCTGCACGCCTG
GGTCCGGGGTGGGCACGGTGCCCGGGCAACGAGCTGAAAGCTCATCTG
CTCTCAGGGGCCCCTCCCTGGGGACAGCCCCTCCTGGCTAGTCACACC
CTGTAGGCTCCTCTATATAACCCAGGGGCACAGGGGCTGCCCTCATTCTA
CCACCACCTCCACAGCACAGACAGACACTCAGGAGCCAGCCAGACCGG
TgccaccATGaagcggactgctgatggcagtgaatttgagtccccaaagaagaagagaaaggtgCT
GAGCATGTACTGCCTGAAGGACAGCAGATTCACCAAGGCCTGGGATAAG
CGGGTGCTGCTGTTCAGAGACATCCTGGCCCAGCTGGGAAGAATCCCC
GCCGAGGCCTACGAGTACTACCACGGCGAGCAGGGTGATAAGAAGAGA
GCTAACGACAATGAGGGCACAAATCCCAAGCGGCACAAGGACAAGTTCA
TCGAATTTGCACTGCACTACCTGGAAGCCCAGCACAGCGAGATCTGCTT
CGGCAGACGCCACATCGTGCGGGAAGAGGCCGGCGCCGGCGATGAGC
ACAAGAAGCACCGGACCAAGGGAAAGGTGGTGGTGGACTTCAGCAAGA
AGGACGAGGACCAGAGCTACTATATCTCCAAGAACAACGTGATCGTGCG
GATCGACAAGAACGCCGGCCCTAGAAGCTACCGGATGGGCCTGAACGA
GCTGAAGTACCTCGTGCTGCTGAGCCTGCAGGGGAAGGGCGACGATGC
CATCGCCAAGCTGTACAGATACAGACAGCACGTGGAGAACATCCTGGAT
GTGGTGAAGGTGACCGATAAGGATAACCACGTGTTCCTGCCCCGCTTCG
TGCTGGAGCAGCACGGCATCGGCAGAAAGGCCTTCAAGCAGCGGATCG
ATGGACGGGTGAAGCACGTGCGGGGCGTGTGGGAGAAGAAGAAGGCC
GCCACCAATGAAATGACCCTGCACGAGAAGGCCAGAGACATCCTGCAGT
ACGTGAACGAAAACTGCACCCGGTCCTTCAACCCTGGCGAATACAACAG
ACTGCTGGTGTGCCTGGTGGGCAAGGACGTGGAGAACTTTCAGGCCGG
CCTGAAGCGGCTGCAGCTGGCCGAAAGGATCGATGGCCGGGTGTACTC
CATCTTCGCCCAGACCAGCACCATCAATGAGATGCACCAGGTGGTGTGC

GACCAGATCCTGAACCGGCTGTGCAGAATCGGCGACCAGAAGCTGTACG
ATTACGTGGGACTGGGCAAGAAGGACGAAATCGACTACAAGCAGAAGGT
GGCCTGGTTCAAGGAGCACATCAGCATCCGGAGAGGATTCCTGAGAAAG
AAGTTCTGGTACGATAGCAAGAAGGGATTCGCAAAGCTGGTGGAGGAAC
ACCTGGAGTCCGGCGGCGGCCAGCGCGACGTGGGCCTGGACAAGAAG
TACTACCACATCGACGCCATCGGCAGATTCGAGGGCGCCAACCCCGCCC
TGTACGAGACCCTGGCCAGAGATCGGCTGTGCCTCATGATGGCCCAGTA
CTTCCTGGGCAGCGTGAGAAAGGAACTGGGCAACAAGATTGTGTGGAGC
AACGACAGCATCGAACTGCCTaagcgcaccgccgatggttccgagttcgaaagccccaaaa
aaaagcgcaaggtcGGTGGAGGCGGAGGTTCTGGGGGAGGAGGTAGTGGCG
GTGGTGGTTCAGGAGGCGGCGGAAGCcagctgcatttaccgcaggttttagctgacgctg
tctcacgcctggtcctgggtaagtttggtgacctgaccgacaacttctcctcccctcacgctcgcagaaaagt
gctggctggagtcgtcatgacaacaggcacagatgttaaagatgccaaggtgataagtgtttctacaggaA
Ccaaatgtattaatggtgaatacatgagtgatcgtggccttgcattaaatgactgccatgcagaaataatatct
cggagatccttgctcagatttctttatacacaacttgagctttacttaaataacaaagatgatcaaaaaagatc
catctttcagaaatcagagcgagggggtttaggctgaaggagaatgtccagtttcatctgtacatcagcac
ctctccctgtggagatgccagaatcttctcaccacatgagccaatcctggaagaaccagcagatagacacc
caaatcgtaaagcaagaggacagctacggaccaaaatagagtctggtcaggggacgattccagtgcgct
ccaatgcgagcatccaaacgtgggacggggtgctgcaaggggagcggctgctcaccatgtcctgcagtg
acaagattgcacgctggaacgtggtgggcatccagggatcactgctcagcattttcgtggagcccatttactt
ctcgagcatcatcctgggcagcctttaccacggggaccacctttccagggccatgtaccagcggatctcca
acatagaggacctgccacctctctacaccctcaacaagcctttgctcagtggcatcagcaatgcagaagca
cggcagccagggaaggcccccaacttcagtgtcaactggacggtaggcgactccgctattgaggtcatca
acgccacgactgggaaggatgagctgggccgcgcgtcccgcctgtgtaagcACGCGTTGTACTG
TCGCTGGATGCGTGTGCACGGCAAGGTTCCCTCCCACTTACTACGCTCC
AAGATTACCAAGCCCAACGTGTACCATGAGTCCAAGCTGGCGGCAAAGG
AGTACCAGGCCGCCAAGGCGCGTCTGTTCACAGCCTTCATCAAGGCGG
GGCTGGGGGCCTGGGTGGAGAAGCCCACCGAGCAGGACCAGTTCTCAC
TCACGGGATCCtacccatacgatgttccagattacgcttatccctacgacgtgcctgattatgcatacc
catatgatgtccccgactatgccGCGGCCGCTGATTACAAAGACGATGACGATAAGtA
AATCGAATTCCGCTCGAGATAATCAACCTCTGGATTACAAAATTTGTGAAA
GATTGACTGGTATTCTTAACTATGTTGCTCCTTTTACGCTATGTGGATACGC
TGCTTTAATGCCTTTGTATCATGCTATTGCTTCCCGTATGGCTTTCATTTTC
TCCTCCTTGTATAAATCCTGGTTAGTTCTTGCCACGGCGGAACTCATCGC
CGCCTGCCTTGCCCGCTGCTGGACAGGGGCTCGGCTGTTGGGCACTGA
CAATTCCGTGGTGTTTATTTGTGAAATTTGTGATGCTATTGCTTTATTTGTA
ACCATCTAGCTTTATTTGTGAAATTTGTGATGCTATTGCTTTATTTGTAACCA
TTATAAGCTGCAATAAACAAGTTAACAACAACAATTGCATTCATTTTATGTTT
CAGGTTCAGGGGGAGATGTGGGAGGTTTTTTAAAGCGGCCGCCGGCCG
AGGGCCGCAGGAACCCCTAGTGATGGAGTTGGCCACTCCCTCTCTGCG
CGCTCGCTCGCTCACTGAGGCCGGGCGACCAAAGGTCGCCCGACGCCC
GGGCTTTGCCCGGGCGGCCTCAGTGAGCGAGCGAGCGCGCAGCTGCC
TGCAGGGGCGCCTGATGCGGTATTTTCTCCTTACGCATCTGTGCGGTATT
TCACACCGCATACGTCAAAGCAACCATAGTACGCGCCCTGTAGCGGCGC

ATTAAGCGCGGCGGGTGTGGTGGTTACGCGCAGCGTGACCGCTACACTT
GCCAGCGCCTTAGCGCCCGCTCCTTTCGCTTTCTTCCCTTCCTTTCTCGC
CACGTTCGCCGGCTTTCCCCGTCAAGCTCTAAATCGGGGGCTCCCTTTA
GGGTTCCGATTTAGTGCTTTACGGCACCTCGACCCCAAAAAACTTGATTT
GGGTGATGGTTCACGTAGTGGGCCATCGCCCTGATAGACGGTTTTTCGC
CCTTTGACGTTGGAGTCCACGTTCTTTAATAGTGGACTCTTGTTCCAAACT
GGAACAACACTCAACTCTATCTCGGGCTATTCTTTTGATTTATAAGGGATTT
TGCCGATTTCGGTCTATTGGTTAAAAAATGAGCTGATTTAACAAAAATTTAA
CGCGAATTTTAACAAAATATTAACGTTTACAATTTTATGGTGCACTCTCAGT
ACAATCTGCTCTGATGCCGCATAGTTAAGCCAGCCCCGACACCCGCCAA
CACCCGCTGACGCGCCCTGACGGGCTTGTCTGCTCCCGGCATCCGCTTA
CAGACAAGCTGTGACCGTCTccgggagctgcatgtgtcagaggttttcaccgtcatcaccgaa
acgcgcgagACGAAAGGGCCTCGTGATACGCCTATTTTTATAGGTTAATGTCA
TGATAATAATGGTTTCTTAGACGTCAGGTGGCACTTTTCGGGGAAATGTGC
GCGGAACCCCTATTTGTTTATTTTTCTAAATACATTCAAATATGTATCCGCTC
ATGAGACAATAACCCTGATAAATGCTTCAATAATATTGAAAAAGGAAGAGTA
TGAGTATTCAACATTTCCGTGTCGCCCTTATTCCCTTTTTTGCGGCATTTT
GCCTTCCTGTTTTTGCTCACCCAGAAACGCTGGTGAAAGTAAAAGATGCT
GAAGATCAGTTGGGTGCACGAGTGGGTTACATCGAACTGGATCTCAACA
GCGGTAAGATCCTTGAGAGTTTTCGCCCCGAAGAACGTTTTCCAATGATG
AGCACTTTTAAAGTTCTGCTATGTGGCGCGGTATTATCCCGTATTGACGCC
GGGCAAGAGCAACTCGGTCGCCGCATACACTATTCTCAGAATGACTTGGT
TGAGTACTCACCAGTCACAGAAAAGCATCTTACGGATGGCATGACAGTAA
GAGAATTATGCAGTGCTGCCATAACCATGAGTGATAACACTGCGGCCAAC
TTACTTCTGACAACGATCGGAGGACCGAAGGAGCTAACCGCTTTTTTGCA
CAACATGGGGGATCATGTAACTCGCCTTGATCGTTGGGAACCGGAGCTG
AATGAAGCCATACCAAACGACGAGCGTGACACCACGATGCCTGTAGCAAT
GGCAACAACGTTGCGCAAACTATTAACTGGCGAACTACTTACTCTAGCTT
CCCGGCAACAATTAATAGACTGGATGGAGGCGGATAAAGTTGCAGGACCA
CTTCTGCGCTCGGCCCTTCCGGCTGGCTGGTTTATTGCTGATAAATCTGG
AGCCGGTGAGCGTGGAAGCCGCGGTATCATTGCAGCACTGGGGCCAGA
TGGTAAGCCCTCCCGTATCGTAGTTATCTACACGACGGGGAGTCAGGCAA
CTATGGATGAACGAAATAGACAGATCGCTGAGATAGGTGCCTCACTGATTA
AGCATTGGTAACTGTCAGACCAAGTTTACTCATATATACTTTAGATTGATTTA
AAACTTCATTTTTAATTTAAAAGGATCTAGGTGAAGATCCTTTTTGATAATCT
CATGACCAAAATCCCTTAACGTGAGTTTTCGTTCCACTGAGCGTCAGACC
CCGTAGAAAAGATCAAAGGATCTTCTTGAGATCCTTTTTTTCTGCGCGTAA
TCTGCTGCTTGCAAACAAAAAAACCACCGCTACCAGCGGTGGTTTGTTTG
CCGGATCAAGAGCTACCAACTCTTTTTCCGAAGGTAACTGGCTTCAGCAG
AGCGCAGATACCAAATACTGTTCTTCTAGTGTAGCCGTAGTTAGGCCACC
ACTTCAAGAACTCTGTAGCACCGCCTACATACCTCGCTCTGCTAATCCTGT
TACCAGTGGCTGCTGCCAGTGGCGATAAGTCGTGTCTTACCGGGTTGGA
CTCAAGACGATAGTTACCGGATAAGGCGCAGCGGTCGGGCTGAACGGG
GGGTTCGTGCACACAGCCCAGCTTGGAGCGAACGACCTACACCGAACT

GAGATACCTACAGCGTGAGCTATGAGAAAGCGCCACGCTTCCCGAAGGG
AGAAAGGCGGACAGGTATCCGGTAAGCGGCAGGGTCGGAACAGGAGAG
CGCACGAGGGAGCTTCCAGGGGGAAACGCCTGGTATCTTTATAGTCCTG
TCGGGTTTCGCCACCTCTGACTTGAGCGTCGATTTTTGTGATGCTCGTCA
GGGGGGCGGAGCCTATGGAAAAACGCCAGCAACGCGGCCTTTTTACGG
TTCCTGGCCTTTTGCTGGCCTTTTGCTCACATGT

>M2
CCTGCAGGCAGCTGCGCGCTCGCTCGCTCACTGAGGCCGCCCGGGCG
TCGGGCGACCTTTGGTCGCCCGGCCTCAGTGAGCGAGCGAGCGCGCA
GAGAGGGAGTGGCCAACTCCATCACTAGGGGTTCCTGCGGCCTCTAGAg
gagggcctatttcccatgattccttcatatttgcatatacgatacaaggctgttagagagataattggaattaatt
tgactgtaaacacaaagatattagtacaaaatacgtgacgtagaaagtaataatttcttgggtagtttgcagttt
taaaattatgttttaaaatggactatcatatgcttaccgtaacttgaaagtatttcgatttcttggctttatatatcttG
TGGAAAGGACGAAACACCGGCTGGAGCAGCCCCCGATTTGTGGGGTGA
TTACAGCGTCTGCAATATAAGCTGCCAACCACTTGTCAATGAATGTGAGG
GACTCCTGCTGGAGCAGCCCCCGATTTGTGGGGTGATTACAGCTTTTTTT
ACCCTTCAGATTAAAAATAACTGAGGTAAGGGCCTGGGTAGGGGAGGTG
GTGTGAGACGCTCCTGTCTCTCCTCTATCTGCCCATCGGCCCTTTGGGG
AGGAGGAATGTGCCCAAGGACTAAAAAAAGGCCATGGAGCCAGAGGGG
CGAGGGCAACAGACCTTTCATGGGCAAACCTTGGGGCCCTGCTGTCTAG
CATGCCCCACTACGGGTCTAGGCTGCCCATGTAAGGAGGCAAGGCCTGG
GGACACCCGAGATGCCTGGTTATAATTAACCCAGACATGTGGCTGCCCCC
CCCCCCCCAACACCTGCTGCCTCTAAAAATAACCCTGTCCCTGGTGGATC
CCCTGCATGCGAAGATCTTCGAACAAGGCTGTGGGGGACTGAGGGCAG
GCTGTAACAGGCTTGGGGGCCAGGGCTTATACGTGCCTGGGACTCCCAA
AGTATTACTGTTCCATGTTCCCGGCGAAGGGCCAGCTGTCCCCCGCCAG
CTAGACTCAGCACTTAGTTTAGGAACCAGTGAGCAAGTCAGCCCTTGGG
GCAGCCCATACAAGGCCATGGGGCTGGGCAAGCTGCACGCCTGGGTCC
GGGGTGGGCACGGTGCCCGGGCAACGAGCTGAAAGCTCATCTGCTCTC
AGGGGCCCCTCCCTGGGGACAGCCCCTCCTGGCTAGTCACACCCTGTA
GGCTCCTCTATATAACCCAGGGGCACAGGGGCTGCCCTCATTCTACCACC
ACCTCCACAGCACAGACAGACACTCAGGAGCCAGCCAGaccggtGCCACC
ATGGCCCCCAAGAAGAAGaGGAAGGTGCTGAGCATGTACTGCCTGAAGG
ACAGCAGATTCACCAAGGCCTGGGATAAGCGGGTGCTGCTGTTCAGAGA
CATCCTGGCCCAGCTGGGAAGAATCCCCGCCGAGGCCTACGAGTACTAC
CACGGCGAGCAGGGTGATAAGAAGAGAGCTAACGACAATGAGGGCACAA
ATCCCAAGCGGCACAAGGACAAGTTCATCGAATTTGCACTGCACTACCTG
GAAGCCCAGCACAGCGAGATCTGCTTCGGCAGACGCCACATCGTGCGG
GAAGAGGCCGGCGCCGGCGATGAGCACAAGAAGCACCGGACCAAGGG
AAAGGTGGTGGTGGACTTCAGCAAGAAGGACGAGGACCAGAGCTACTAT
ATCTCCAAGAACAACGTGATCGTGCGGATCGACAAGAACGCCGGCCCTA
GAAGCTACCGGATGGGCCTGAACGAGCTGAAGTACCTCGTGCTGCTGAG
CCTGCAGGGGAAGGGCGACGATGCCATCGCCAAGCTGTACAGATACAGA

CAGCACGTGGAGAACATCCTGGATGTGGTGAAGGTGACCGATAAGGATAACCACGTGTTCCTGCCCCGCTTCGTGCTGGAGCAGCACGGCATCGGCAGAAAGGCCTTCAAGCAGCGGATCGATGGACGGGTGAAGCACGTGCGGGGCGTGTGGGAGAAGAAGAAGGCCGCCACCAATGAAATGACCCTGCACGAGAAGGCCAGAGACATCCTGCAGTACGTGAACGAAAACTGCACCCGGTCCTTCAACCCTGGCGAATACAACAGACTGCTGGTGTGCCTGGTGGGCAAGGACGTGGAGAACTTTCAGGCCGGCCTGAAGCGGCTGCAGCTGGCCGAAAGGATCGATGGCCGGGTGTACTCCATCTTCGCCCAGACCAGCACCATCAATGAGATGCACCAGGTGGTGTGCGACCAGATCCTGAACCGGCTGTGCAGAATCGGCGACCAGAAGCTGTACGATTACGTGGGACTGGGCAAGAAGGACGAAATCGACTACAAGCAGAAGGTGGCCTGGTTCAAGGAGCACATCAGCATCCGGAGAGGATTCCTGAGAAAGAAGTTCTGGTACGATAGCAAGAAGGGATTCGCAAAGCTGGTGGAGGAACACCTGGAGTCCGGCGGCGGCCAGCGCGACGTGGGCCTGGACAAGAAGTACTACCACATCGACGCCATCGGCAGATTCGAGGGCGCCAACCCCGCCCTGTACGAGACCCTGGCCAGAGATCGGCTGTGCCTCATGATGGCCCAGTACTTCCTGGGCAGCGTGAGAAAGGAACTGGGCAACAAGATTGTGTGGAGCAACGACAGCATCGAACTGCCTCCCAAGAAGAAGCGGAAGGTGGGTGGAGGCGGAGGTTCTGGGGGAGGAGGTAGTGGCGGTGGTGGTTCAGGAGGCGGCGGAAGCcagctgcatttaccgcaggttttagctgacgctgtctcacgcctggtcctgggtaagtttggtgacctgaccgacaacttctcctcccctcacgctcgcagaaaagtgctggctggagtcgtcatgacaacaggcacagatgttaaagatgccaaggtgataagtgtttctacaggaACcaaatgtattaatggtgaatacatgagtgatcgtggccttgcattaaatgactgccatgcagaaataatatctcggagatccttgctcagatttctttatacacaacttgagctttacttaaataacaaagatgatcaaaaaagatccatctttcagaaatcagagcgaggggggtttaggctgaaggagaatgtccagtttcatctgtacatcagcacctctccctgtggagatgccagaatcttctcaccacatgagccaatcctggaagaaccagcagatagacacccaaatcgtaaagcaagaggacagctacggaccaaaatagagtctggtcaggggacgattccagtgcgctccaatgcgagcatccaaacgtgggacggggtgctgcaaggggagcggctgctcaccatgtcctgcagtgacaagattgcacgctggaacgtggtgggcatccagggatcactgctcagcattttcgtggagcccatttacttctcgagcatcatcctgggcagcctttaccacggggaccacctttccagggccatgtaccagcggatctccaacatagaggacctgccacctctctacaccctcaacaagcctttgctcagtggcatcagcaatgcagaagcacggcagccagggaaggcccccaacttcagtgtcaactggacggtaggcgactccgctattgaggtcatcaacgccacgactgggaaggatgagctgggccgcgcgtcccgcctgtgtaagcACGCGTTGTACTGTCGCTGGATGCGTGTGCACGGCAAGGTTCCCTCCCACTTACTACGCTCCAAGATTACCAAGCCCAACGTGTACCATGAGTCCAAGCTGGCGGCAAAGGAGTACCAGGCCGCCAAGGCGCGTCTGTTCACAGCCTTCATCAAGGCGGGGCTGGGGGCCTGGGTGGAGAAGCCCACCGAGCAGGACCAGTTCTCACTCACGAAAAGGCCGGCGGCCACGAAAAAGGCCGGCCAGGCAAAAAAGAAAAAGtacccatacgatgttccagattacgcttatccctacgacgtgcctgattatgcataccatatgatgtccccgactatgccGCGGCCGCTGATTACAAAGACGATGACGATAAGtaaATCGAATTCCGCTCGAGATAATCAACCTCTGGATTACAAAATTTGTGAAAGATTGACTGGTATTCTTAACTATGTTGCTCCTTTTACGCTATGTGGATACGCTGCTTTAATGCCTTTGTATCATGCTATTGCTTCCCGTATGGCTTTCATTTTCTCCTCCTTGTATAAATCCTGGTTAGTTCTTGCCACGGCGGAACTCATCGCCGCCTGCCTTGCCCGCTGCTGGACAGGGGCTCGGCTGTTGGGCACTGAC

AATTCCGTGGTGTTTATTTGTGAAATTTGTGATGCTATTGCTTTATTTGTAA
CCATCTAGCTTTATTTGTGAAATTTGTGATGCTATTGCTTTATTTGTAACCAT
TATAAGCTGCAATAAACAAGTTAACAACAACAATTGCATTCATTTTATGTTTC
AGGTTCAGGGGGAGATGTGGGAGGTTTTTTAAAGCGGCCGCCggccgagG
GCCGCAGGAACCCCTAGTGATGGAGTTGGCCACTCCCTCTCTGCGCGCT
CGCTCGCTCACTGAGGCCGGGCGACCAAAGGTCGCCCGACGCCCGGG
CTTTGCCCGGGCGGCCTCAGTGAGCGAGCGAGCGCGCAGCTGCCTGCA
GGGGCGCCTGATGCGGTATTTTCTCCTTACGCATCTGTGCGGTATTTCAC
ACCGCATACGTCAAAGCAACCATAGTACGCGCCCTGTAGCGGCGCATTAA
GCGCGGCGGGTGTGGTGGTTACGCGCAGCGTGACCGCTACACTTGCCA
GCGCCTTAGCGCCCGCTCCTTTCGCTTTCTTCCCTTCCTTTCTCGCCACG
TTCGCCGGCTTTCCCCGTCAAGCTCTAAATCGGGGGCTCCCTTTAGGGT
TCCGATTTAGTGCTTTACGGCACCTCGACCCCAAAAAACTTGATTTGGGT
GATGGTTCACGTAGTGGGCCATCGCCCTGATAGACGGTTTTTCGCCCTTT
GACGTTGGAGTCCACGTTCTTTAATAGTGGACTCTTGTTCCAAACTGGAA
CAACACTCAACTCTATCTCGGGCTATTCTTTTGATTTATAAGGGATTTTGCC
GATTTCGGTCTATTGGTTAAAAAATGAGCTGATTTAACAAAAATTTAACGCG
AATTTTAACAAAATATTAACGTTTACAATTTTATGGTGCACTCTCAGTACAAT
CTGCTCTGATGCCGCATAGTTAAGCCAGCCCCGACACCCGCCAACACCC
GCTGACGCGCCCTGACGGGCTTGTCTGCTCCCGGCATCCGCTTACAGAC
AAGCTGTGACCGTCTccgggagctgcatgtgtcagaggttttcaccgtcatcaccgaaacgcgcg
agACGAAAGGGCCTCGTGATACGCCTATTTTTATAGGTTAATGTCATGATAA
TAATGGTTTCTTAGACGTCAGGTGGCACTTTTCGGGGAAATGTGCGCGGA
ACCCCTATTTGTTTATTTTTCTAAATACATTCAAATATGTATCCGCTCATGAG
ACAATAACCCTGATAAATGCTTCAATAATATTGAAAAAGGAAGAGTATGAGT
ATTCAACATTTCCGTGTCGCCCTTATTCCCTTTTTTGCGGCATTTTGCCTT
CCTGTTTTTGCTCACCCAGAAACGCTGGTGAAAGTAAAAGATGCTGAAGA
TCAGTTGGGTGCACGAGTGGGTTACATCGAACTGGATCTCAACAGCGGT
AAGATCCTTGAGAGTTTTCGCCCCGAAGAACGTTTTCCAATGATGAGCAC
TTTTAAAGTTCTGCTATGTGGCGCGGTATTATCCCGTATTGACGCCGGGCA
AGAGCAACTCGGTCGCCGCATACACTATTCTCAGAATGACTTGGTTGAGT
ACTCACCAGTCACAGAAAAGCATCTTACGGATGGCATGACAGTAAGAGAA
TTATGCAGTGCTGCCATAACCATGAGTGATAACACTGCGGCCAACTTACTT
CTGACAACGATCGGAGGACCGAAGGAGCTAACCGCTTTTTTGCACAACA
TGGGGGATCATGTAACTCGCCTTGATCGTTGGGAACCGGAGCTGAATGA
AGCCATACCAAACGACGAGCGTGACACCACGATGCCTGTAGCAATGGCA
ACAACGTTGCGCAAACTATTAACTGGCGAACTACTTACTCTAGCTTCCCG
GCAACAATTAATAGACTGGATGGAGGCGGATAAAGTTGCAGGACCACTTC
TGCGCTCGGCCCTTCCGGCTGGCTGGTTTATTGCTGATAAATCTGGAGCC
GGTGAGCGTGGAAGCCGCGGTATCATTGCAGCACTGGGGCCAGATGGTA
AGCCCTCCCGTATCGTAGTTATCTACACGACGGGGAGTCAGGCAACTATG
GATGAACGAAATAGACAGATCGCTGAGATAGGTGCCTCACTGATTAAGCA
TTGGTAACTGTCAGACCAAGTTTACTCATATATACTTTAGATTGATTTAAAAC
TTCATTTTTAATTTAAAAGGATCTAGGTGAAGATCCTTTTTGATAATCTCATG

ACCAAAATCCCTTAACGTGAGTTTTCGTTCCACTGAGCGTCAGACCCCGT
AGAAAAGATCAAAGGATCTTCTTGAGATCCTTTTTTTCTGCGCGTAATCTG
CTGCTTGCAAACAAAAAAACCACCGCTACCAGCGGTGGTTTGTTTGCCG
GATCAAGAGCTACCAACTCTTTTTCCGAAGGTAACTGGCTTCAGCAGAGC
GCAGATACCAAATACTGTTCTTCTAGTGTAGCCGTAGTTAGGCCACCACTT
CAAGAACTCTGTAGCACCGCCTACATACCTCGCTCTGCTAATCCTGTTACC
AGTGGCTGCTGCCAGTGGCGATAAGTCGTGTCTTACCGGGTTGGACTCA
AGACGATAGTTACCGGATAAGGCGCAGCGGTCGGGCTGAACGGGGGGT
TCGTGCACACAGCCCAGCTTGGAGCGAACGACCTACACCGAACTGAGAT
ACCTACAGCGTGAGCTATGAGAAAGCGCCACGCTTCCCGAAGGGAGAAA
GGCGGACAGGTATCCGGTAAGCGGCAGGGTCGGAACAGGAGAGCGCAC
GAGGGAGCTTCCAGGGGGAAACGCCTGGTATCTTTATAGTCCTGTCGGG
TTTCGCCACCTCTGACTTGAGCGTCGATTTTTGTGATGCTCGTCAGGGGG
GCGGAGCCTATGGAAAAACGCCAGCAACGCGGCCTTTTTACGGTTCCTG
GCCTTTTGCTGGCCTTTTGCTCACATGT

>M3
CCTGCAGGCAGCTGCGCGCTCGCTCGCTCACTGAGGCCGCCCGGGCG
TCGGGCGACCTTTGGTCGCCCGGCCTCAGTGAGCGAGCGAGCGCGCA
GAGAGGGAGTGGCCAACTCCATCACTAGGGGTTCCTGCGGCCTCTAGAg
gagggcctatttcccatgattccttcatatttgcatatacgatacaaggctgttagagagataattggaattaatt
tgactgtaaacacaaagatattagtacaaaatacgtgacgtagaaagtaataatttcttgggtagtttgcagttt
taaaattatgttttaaaatggactatcatatgcttaccgtaacttgaaagtatttcgatttcttggctttatatatcttG
TGGAAAGGACGAAACACCGGTCTGCAATATAAGCTGCCAACCACTTGTCA
ATGAATGTGAGGGACTCCTGCTGGAGCAGCCCCCGATTTGTGGGGTGAT
TACAGCTTTTTTTACCCTTCAGATTAAAAATAACTGAGGTAAGGGCCTGGG
TAGGGGAGGTGGTGTGAGACGCTCCTGTCTCTCCTCTATCTGCCCATCG
GCCCTTTGGGGAGGAGGAATGTGCCCAAGGACTAAAAAAAGGCCATGGA
GCCAGAGGGGCGAGGGCAACAGACCTTTCATGGGCAAACCTTGGGGCC
CTGCTGTCTAGCATGCCCCACTACGGGTCTAGGCTGCCCATGTAAGGAG
GCAAGGCCTGGGGACACCCGAGATGCCTGGTTATAATTAACCCAGACATG
TGGCTGCCCCCCCCCCCCCAACACCTGCTGCCTCTAAAAATAACCCTGT
CCCTGGTGGATCCCCTGCATGCGAAGATCTTCGAACAAGGCTGTGGGGG
ACTGAGGGCAGGCTGTAACAGGCTTGGGGGCCAGGGCTTATACGTGCCT
GGGACTCCCAAAGTATTACTGTTCCATGTTCCCGGCGAAGGGCCAGCTG
TCCCCCGCCAGCTAGACTCAGCACTTAGTTTAGGAACCAGTGAGCAAGT
CAGCCCTTGGGGCAGCCCATACAAGGCCATGGGGCTGGGCAAGCTGCA
CGCCTGGGTCCGGGGTGGGCACGGTGCCCGGGCAACGAGCTGAAAGC
TCATCTGCTCTCAGGGGCCCCTCCCTGGGGACAGCCCCTCCTGGCTAGT
CACACCCTGTAGGCTCCTCTATATAACCCAGGGGCACAGGGGCTGCCCT
CATTCTACCACCACCTCCACAGCACAGACAGACACTCAGGAGCCAGCCA
GaccggtGCCACCATGGCCCCCAAGAAGAAGaGGAAGGTGCTGAGCATGT
ACTGCCTGAAGGACAGCAGATTCACCAAGGCCTGGGATAAGCGGGTGCT
GCTGTTCAGAGACATCCTGGCCCAGCTGGGAAGAATCCCCGCCGAGGC

CTACGAGTACTACCACGGCGAGCAGGGTGATAAGAAGAGAGCTAACGAC
AATGAGGGCACAAATCCCAAGCGGCACAAGGACAAGTTCATCGAATTTGC
ACTGCACTACCTGGAAGCCCAGCACAGCGAGATCTGCTTCGGCAGACGC
CACATCGTGCGGGAAGAGGCCGGCGCCGGCGATGAGCACAAGAAGCAC
CGGACCAAGGGAAAGGTGGTGGTGGACTTCAGCAAGAAGGACGAGGAC
CAGAGCTACTATATCTCCAAGAACAACGTGATCGTGCGGATCGACAAGAA
CGCCGGCCCTAGAAGCTACCGGATGGGCCTGAACGAGCTGAAGTACCTC
GTGCTGCTGAGCCTGCAGGGGAAGGGCGACGATGCCATCGCCAAGCTG
TACAGATACAGACAGCACGTGGAGAACATCCTGGATGTGGTGAAGGTGA
CCGATAAGGATAACCACGTGTTCCTGCCCCGCTTCGTGCTGGAGCAGCA
CGGCATCGGCAGAAAGGCCTTCAAGCAGCGGATCGATGGACGGGTGAA
GCACGTGCGGGGCGTGTGGGAGAAGAAGAAGGCCGCCACCAATGAAAT
GACCCTGCACGAGAAGGCCAGAGACATCCTGCAGTACGTGAACGAAAAC
TGCACCCGGTCCTTCAACCCTGGCGAATACAACAGACTGCTGGTGTGCC
TGGTGGGCAAGGACGTGGAGAACTTTCAGGCCGGCCTGAAGCGGCTGC
AGCTGGCCGAAAGGATCGATGGCCGGGTGTACTCCATCTTCGCCCAGAC
CAGCACCATCAATGAGATGCACCAGGTGGTGTGCGACCAGATCCTGAAC
CGGCTGTGCAGAATCGGCGACCAGAAGCTGTACGATTACGTGGGACTGG
GCAAGAAGGACGAAATCGACTACAAGCAGAAGGTGGCCTGGTTCAAGGA
GCACATCAGCATCCGGAGAGGATTCCTGAGAAAGAAGTTCTGGTACGATA
GCAAGAAGGGATTCGCAAAGCTGGTGGAGGAACACCTGGAGTCCGGCG
GCGGCCAGCGCGACGTGGGCCTGGACAAGAAGTACTACCACATCGACG
CCATCGGCAGATTCGAGGGCGCCAACCCCGCCCTGTACGAGACCCTGG
CCAGAGATCGGCTGTGCCTCATGATGGCCCAGTACTTCCTGGGCAGCGT
GAGAAAGGAACTGGGCAACAAGATTGTGTGGAGCAACGACAGCATCGAA
CTGCCTCCCAAGAAGAAGCGGAAGGTGGGTGGAGGCGGAGGTTCTGGG
GGAGGAGGTAGTGGCGGTGGTGGTTCAGGAGGCGGCGGAAGCcagctgca
tttaccgcaggttttagctgacgctgtctcacgcctggtcctgggtaagtttggtgacctgaccgacaacttctc
ctcccctcacgctcgcagaaaagtgctggctggagtcgtcatgacaacaggcacagatgttaaagatgcc
aaggtgataagtgtttctacaggaACcaaatgtattaatggtgaatacatgagtgatcgtggccttgcattaa
atgactgccatgcagaaataatatctcggagatccttgctcagatttctttatacacaacttgagctttacttaaa
taacaaagatgatcaaaaaagatccatctttcagaaatcagagcgaggggggtttaggctgaaggagaat
gtccagtttcatctgtacatcagcacctctccctgtggagatgccagaatcttctcaccacatgagccaatcct
ggaagaaccagcagatagacacccaaatcgtaaagcaagaggacagctacggaccaaaatagagtct
ggtcaggggacgattccagtgcgctccaatgcgagcatccaaacgtgggacggggtgctgcaaggggа
gcggctgctcaccatgtcctgcagtgacaagattgcacgctggaacgtggtgggcatccagggatcactgc
tcagcattttcgtggagcccatttacttctcgagcatcatcctgggcagcctttaccacggggaccacctttcca
gggccatgtaccagcggatctccaacatagaggacctgccacctctctacaccctcaacaagcctttgctc
agtggcatcagcaatgcagaagcacggcagccagggaaggcccccaacttcagtgtcaactggacggt
aggcgactccgctattgaggtcatcaacgccacgactgggaaggatgagctgggccgcgcgtcccgcct
gtgtaagcACGCGTTGTACTGTCGCTGGATGCGTGTGCACGGCAAGGTTCC
CTCCCACTTACTACGCTCCAAGATTACCAAGCCCAACGTGTACCATGAGT
CCAAGCTGGCGGCAAAGGAGTACCAGGCCGCCAAGGCGCGTCTGTTCA
CAGCCTTCATCAAGGCGGGGCTGGGGGCCTGGGTGGAGAAGCCCACCG

AGCAGGACCAGTTCTCACTCACGAAAAGGCCGGCGGCCACGAAAAAGG
CCGGCCAGGCAAAAAAGAAAAAGtacccatacgatgttccagattacgcttatccctacgac
gtgcctgattatgcatacccatatgatgtccccgactatgccGCGGCCGCTGATTACAAAGAC
GATGACGATAAGtaaATCGAATTCCGCTCGAGATAATCAACCTCTGGATTAC
AAAATTTGTGAAAGATTGACTGGTATTCTTAACTATGTTGCTCCTTTTACGC
TATGTGGATACGCTGCTTTAATGCCTTTGTATCATGCTATTGCTTCCCGTAT
GGCTTTCATTTTCTCCTCCTTGTATAAATCCTGGTTAGTTCTTGCCACGGC
GGAACTCATCGCCGCCTGCCTTGCCCGCTGCTGGACAGGGGCTCGGCT
GTTGGGCACTGACAATTCCGTGGTGTTTATTTGTGAAATTTGTGATGCTAT
TGCTTTATTTGTAACCATCTAGCTTTATTTGTGAAATTTGTGATGCTATTGCT
TTATTTGTAACCATTATAAGCTGCAATAAACAAGTTAACAACAACAATTGCA
TTCATTTTATGTTTCAGGTTCAGGGGGAGATGTGGGAGGTTTTTTAAAGC
GGCCGCCggccgagGGCCGCAGGAACCCCTAGTGATGGAGTTGGCCACTC
CCTCTCTGCGCGCTCGCTCGCTCACTGAGGCCGGGCGACCAAAGGTCG
CCCGACGCCCGGGCTTTGCCCGGGCGGCCTCAGTGAGCGAGCGAGCG
CGCAGCTGCCTGCAGGGGCGCCTGATGCGGTATTTTCTCCTTACGCATC
TGTGCGGTATTTCACACCGCATACGTCAAAGCAACCATAGTACGCGCCCT
GTAGCGGCGCATTAAGCGCGGCGGGTGTGGTGGTTACGCGCAGCGTGA
CCGCTACACTTGCCAGCGCCTTAGCGCCCGCTCCTTTCGCTTTCTTCCCT
TCCTTTCTCGCCACGTTCGCCGGCTTTCCCCGTCAAGCTCTAAATCGGG
GGCTCCCTTTAGGGTTCCGATTTAGTGCTTTACGGCACCTCGACCCCAAA
AAACTTGATTTGGGTGATGGTTCACGTAGTGGGCCATCGCCCTGATAGAC
GGTTTTTCGCCCTTTGACGTTGGAGTCCACGTTCTTTAATAGTGGACTCTT
GTTCCAAACTGGAACAACACTCAACTCTATCTCGGGCTATTCTTTTGATTT
ATAAGGGATTTTGCCGATTTCGGTCTATTGGTTAAAAAATGAGCTGATTTAA
CAAAAATTTAACGCGAATTTTAACAAAATATTAACGTTTACAATTTTATGGTG
CACTCTCAGTACAATCTGCTCTGATGCCGCATAGTTAAGCCAGCCCCGAC
ACCCGCCAACACCCGCTGACGCGCCCTGACGGGCTTGTCTGCTCCCGG
CATCCGCTTACAGACAAGCTGTGACCGTCTccgggagctgcatgtgtcagaggttttca
ccgtcatcaccgaaacgcgcgagACGAAAGGGCCTCGTGATACGCCTATTTTTATAG
GTTAATGTCATGATAATAATGGTTTCTTAGACGTCAGGTGGCACTTTTCGG
GGAAATGTGCGCGGAACCCCTATTTGTTTATTTTTCTAAATACATTCAAATA
TGTATCCGCTCATGAGACAATAACCCTGATAAATGCTTCAATAATATTGAAA
AAGGAAGAGTATGAGTATTCAACATTTCCGTGTCGCCCTTATTCCCTTTTT
TGCGGCATTTTGCCTTCCTGTTTTTGCTCACCCAGAAACGCTGGTGAAAG
TAAAAGATGCTGAAGATCAGTTGGGTGCACGAGTGGGTTACATCGAACTG
GATCTCAACAGCGGTAAGATCCTTGAGAGTTTTCGCCCCGAAGAACGTTT
TCCAATGATGAGCACTTTTAAAGTTCTGCTATGTGGCGCGGTATTATCCCG
TATTGACGCCGGGCAAGAGCAACTCGGTCGCCGCATACACTATTCTCAGA
ATGACTTGGTTGAGTACTCACCAGTCACAGAAAAGCATCTTACGGATGGC
ATGACAGTAAGAGAATTATGCAGTGCTGCCATAACCATGAGTGATAACACT
GCGGCCAACTTACTTCTGACAACGATCGGAGGACCGAAGGAGCTAACCG
CTTTTTTGCACAACATGGGGGATCATGTAACTCGCCTTGATCGTTGGGAA
CCGGAGCTGAATGAAGCCATACCAAACGACGAGCGTGACACCACGATGC

CTGTAGCAATGGCAACAACGTTGCGCAAACTATTAACTGGCGAACTACTTA
CTCTAGCTTCCCGGCAACAATTAATAGACTGGATGGAGGCGGATAAAGTT
GCAGGACCACTTCTGCGCTCGGCCCTTCCGGCTGGCTGGTTTATTGCTG
ATAAATCTGGAGCCGGTGAGCGTGGAAGCCGCGGTATCATTGCAGCACT
GGGGCCAGATGGTAAGCCCTCCCGTATCGTAGTTATCTACACGACGGGG
AGTCAGGCAACTATGGATGAACGAAATAGACAGATCGCTGAGATAGGTGC
CTCACTGATTAAGCATTGGTAACTGTCAGACCAAGTTTACTCATATATACTT
TAGATTGATTTAAAACTTCATTTTTAATTTAAAAGGATCTAGGTGAAGATCCT
TTTTGATAATCTCATGACCAAAATCCCTTAACGTGAGTTTTCGTTCCACTG
AGCGTCAGACCCCGTAGAAAAGATCAAAGGATCTTCTTGAGATCCTTTTTT
TCTGCGCGTAATCTGCTGCTTGCAAACAAAAAAACCACCGCTACCAGCG
GTGGTTTGTTTGCCGGATCAAGAGCTACCAACTCTTTTTCCGAAGGTAAC
TGGCTTCAGCAGAGCGCAGATACCAAATACTGTTCTTCTAGTGTAGCCGT
AGTTAGGCCACCACTTCAAGAACTCTGTAGCACCGCCTACATACCTCGCT
CTGCTAATCCTGTTACCAGTGGCTGCTGCCAGTGGCGATAAGTCGTGTCT
TACCGGGTTGGACTCAAGACGATAGTTACCGGATAAGGCGCAGCGGTCG
GGCTGAACGGGGGGTTCGTGCACACAGCCCAGCTTGGAGCGAACGACC
TACACCGAACTGAGATACCTACAGCGTGAGCTATGAGAAAGCGCCACGCT
TCCCGAAGGGAGAAAGGCGGACAGGTATCCGGTAAGCGGCAGGGTCGG
AACAGGAGAGCGCACGAGGGAGCTTCCAGGGGGAAACGCCTGGTATCT
TTATAGTCCTGTCGGGTTTCGCCACCTCTGACTTGAGCGTCGATTTTTGT
GATGCTCGTCAGGGGGGCGGAGCCTATGGAAAAACGCCAGCAACGCGG
CCTTTTTACGGTTCCTGGCCTTTTGCTGGCCTTTTGCTCACATGT
